# Establishment of a large-scale patient-derived high-risk colorectal adenoma organoid biobank for high-throughput and high-content drug screening

**DOI:** 10.1186/s12916-023-03034-y

**Published:** 2023-09-04

**Authors:** Zhongguang Luo, Bangting Wang, Feifei Luo, Yumeng Guo, Ning Jiang, Jinsong Wei, Xin Wang, Yujen Tseng, Jian Chen, Bing Zhao, Jie Liu

**Affiliations:** 1 Department of Digestive Diseases, Huashan Hospital, Fudan University, Shanghai, 200040 China; 2 State Key Laboratory of Genetic Engineering, School of Life Sciences, Zhongshan Hospital, Fudan University, Shanghai, 200438 China; 3 Institute of Organoid Technology, Kunming Medical University, Kunming, 650500 China; 4 Institute of Biomedical Sciences and Department of Immunology, School of Basic Medical Sciences, Fudan University, Shanghai, 200032 China

**Keywords:** Patient-derived high-risk colorectal adenoma organoid (HRCA-PDO), Biobank, High throughput drug screening, Stemness, Metformin

## Abstract

**Background:**

Colorectal adenoma (CA), especially high-risk CA (HRCA), is a precancerous lesion with high prevalence and recurrence rate and accounts for about 90% incidence of sporadic colorectal cancer cases worldwide. Currently, recurrent CA can only be treated with repeated invasive polypectomies, while safe and promising pharmaceutical invention strategies are still missing due to the lack of reliable in vitro model for CA-related drug screening.

**Methods:**

We have established a large-scale patient-derived high-risk colorectal adenoma organoid (HRCA-PDO) biobank containing 37 PDO lines derived from 33 patients and then conducted a series of high-throughput and high-content HRCA drug screening.

**Results:**

We established the primary culture system with the non-WNT3a medium which highly improved the purity while maintained the viability of HRCA-PDOs. We also proved that the HRCA-PDOs replicated the histological features, cellular diversity, genetic mutations, and molecular characteristics of the primary adenomas. Especially, we identified the dysregulated stem genes including *LGR5*, *c-Myc*, and *OLFM4* as the markers of adenoma, which are well preserved in HRCA-PDOs. Based on the HRCA-PDO biobank, a customized 139 compound library was applied for drug screening. Four drugs including metformin, BMS754807, panobinostat and AT9283 were screened out as potential hits with generally consistent inhibitory efficacy on HRCA-PDOs. As a representative, metformin was discovered to hinder HRCA-PDO growth in vitro and in vivo by restricting the stemness maintenance.

**Conclusions:**

This study established a promising HRCA-PDO biobank and conducted the first high-throughput and high-content HRCA drug screening in order to shed light on the prevention of colorectal cancer.

**Supplementary Information:**

The online version contains supplementary material available at 10.1186/s12916-023-03034-y.

## Background

Colorectal carcinoma is one of the major forms of cancer worldwide with increasing morbidity and mortality [1]. Dysregulation of a series of molecular pathways, including Wnt, Ras, TGF-β, and DNA mismatch repair, drives the mucosa-to-carcinoma pathogenesis [2]. However, it is challenging to unravel the functional contribution of each carcinoma mutation due to the complex combination of genetic alterations related with microsatellite or chromosome instability. Around 85–90% of sporadic colorectal carcinomas arise from premalignant polyps, represented by colorectal adenoma (CA), especially high-risk CA (HRCA) [3, 4]. HRCA is a special stage during normal mucosa to adenocarcinoma transition with a carcinogenesis rate of about 25% [5]. Although colonoscopy is an effective way to detect and remove precancerous CA for carcinoma prevention [5], this invasive surgery could be frequent and torturous, which greatly hinders patients’ active participation, especially for those with recurrent or multiple CAs [6].

The initiation of CA is highly associated with dysregulation of several signaling pathways, including Wnt/β-catenin [7], TGF-β/SMAD [8], JAK/STAT [9], MAPK [10], DNA mismatch repair [11], and so on. Although there are several drugs currently tested in clinical trials for CA treatment including COX-2 selective inhibitor (celecoxib) [12], aspirin [13, 14], and nonsteroidal anti-inflammatory drug (ibuprofen) [14], the adverse effects such as greater cardiovascular or bleeding risks [12, 13] were not neglectable. There are also other potential drugs for adenoma intervention including metformin. Although metformin is traditionally used as the first-line pharmacologic treatment for type 2 diabetes due to its safety, efficacy, and tolerability [15], it also has the potential for polyp prevention while the underlying mechanisms remain unclear [16]. It is imperative to find safe and reliable drugs with determined mechanisms of action to target CA/HRCA for its recurrence and progression in clinical treatment [17].

The major dilemma regarding the drug discovery targeting HRCA is the lack of in vitro model for drug screening and mechanistic studies. Organoids are stem cell-originated self-organized 3D cell clusters. The organoid culture system better mimics the growth environment of cells in vivo, which could accurately maintain the cellular, molecular, and functional characteristics of original organs or tissues [18]. For tumors, patient-derived organoids (PDOs) recapitulate the heterogenetic mutations and cancer treatment responses of individuals, which hold great promise for cancer modeling and personalized medicine [18]. As organoids can be amplified and manipulated in vitro, PDOs demonstrate better clinical application prospects, especially in large-scale compound screening, as well as precisely personalized medication. In 2011, Hans Clevers team established culture systems for human normal mucosa, *APC*^min/+^ mouse intestinal adenoma and colorectal cancer organoids [19]. In 2016, Fuji Masayuki et al. further modified the cultivating conditions of human colorectal adenoma and adenocarcinoma [20]. Lately, Ganesh Karuna et al. and Ye Yao et al. have generated colorectal cancer organoid biobanks, which demonstrated the high consistency of the drug susceptibility between results in colorectal cancer PDOs and clinical outcome [21, 22]. However, to our knowledge, there is no large-scale CA/HRCA-PDO biobank available for adenoma-targeted drug screening. In order to facilitate the reliable, efficient, and precise drug screening, it is necessary to make sure of the purity, in vivo-imitability, and heterogeneity of the organoids.

Here, we explored organoid technology and successfully established a biobank comprised of 37 human HRCA-PDO lines originated from 33 patients. The HRCA-PDO culture is highly pure and generally retained the mucosa-to-carcinoma transiting signature with individual histopathology and genetic fingerprint preserved. Based on this organoid biobank, we established a high-throughput and high-content screening platform and conducted the first drug screening for HRCA to date. Among 139 drugs, we screened out top four candidate hits including metformin with potential of inhibiting HRCA-PDO growth. Metformin exerted both in vitro and in vivo adenoma-inhibition efficacy on HRCA-PDO lines and AOM/DSS-treated mice respectively, probably by downregulating expression of stemness genes. To summarize, this study established a HRCA-PDO biobank which could be promising for HRCA-targeted drug discovery.

## Methods

### Organoid culture of patient-derived normal colorectal mucosa and adenoma tissues

Human adenoma biopsy samples were obtained from patients (40–70 years old) for research purpose under the approval of the Ethical Committee of Medical Research, Huashan Hospital of Fudan University (2018-182). Informed consent had been obtained from all subjects involved.

Human colorectal adenoma samples were minced into 3 mm^3^, then washed with PBS three times. The tissue fragments were digested by collagenase solution (collagenase 0.125 mg/mL, dispase 0.5 mg/mL, FBS 1% in DMEM medium) for 50 min at 37 °C. Isolated epithelium was mixed with Matrigel (Corning) and cultured as previously described [19]. Briefly, CA organoid culture medium was the basal medium supplemented with 500 ng/mL RSPO1 (R&D system, Minneapolis, MN, USA), 100 ng/mL Noggin (R&D), 50 ng/mL EGF (Invitrogen, Carlsbad, USA), 10 nM gastrin (Sigma-Aldrich, St Louis, MO, USA), 500 nM A83-01, and 10 μM SB202190. In order to determine the optimum culture medium with selectivity for CA organoids, we compared CA medium with or without 10 ng/mL Wnt3a. Culture medium was changed every other day. Within 1 week, organoids were passaged at a 1:3 ratio with TrypLE digestion.

### Generation of AOM/DSS-induced adenoma mouse model

The C57BL/6 mice were purchased from Shanghai Research Center for Model Organism. All animal studies were performed in accordance with the relevant guidelines and under the approval of the Institutional Animal Care and Use Committee of Fudan University and carried out in compliance with the Guide for the Care and Use of Laboratory Animals published by the National Institutes of Health (8th Edition, 2011; https://www.ncbi.nlm.nih.gov/books/NBK54050/). All mice were housed in a room under controlled temperature (23 ± 1 °C) and 12-h light-dark cycles, with free access to water and standard mouse chow (LabDiet 5053; LabDiet, Purina Mills, Richmond, IN).

AOM/DSS were administrated to induce colorectal adenoma. Six-week-old male C57BL/6 J mice were injected with 10 mg/kg AOM intraperitoneally [23]. After 1 week, the mice were fed with two rounds of DSS (2.5% DSS in water for 5 days followed by a nine-day recovery period). After adenoma formation (5 weeks post AOM injection), mice were randomly divided into two groups and intraperitoneally injected with either 250 mg/kg of metformin in phosphate buffer saline (PBS) or PBS (control group) every day for 3 weeks. The mice were sacrificed at ten weeks post AOM injection for collection of colorectal tumors/adenomas to assess the number and size, as well as protein and mRNA expressions.

### Immunohistochemical staining

After fixation of 4% formalin for 1 h, organoids were washed and resuspended in 60 μL of warm (45°C) 1% agarose. After solidification and gradient dehydration, the agarose containing organoids was embedded in paraffin and underwent routine sectioning at the thickness of 5 μm. The primary adenoma tissues were fixed by 4% formalin followed by routine dehydration and sectioning. For morphological analysis, adenoma or organoid sections were prepared for hematoxylin eosin staining. For Immunohistochemical staining, sections were incubated with primary antibodies at 4 °C overnight, including anti-Ki-67 (CST, 1:200), anti-OLFM4 (CST, 1:200), and c-Myc (GeneTex, 1:200). Followed by PBS washing, secondary antibody conjugated with biotin was incubated at room temperature for 1 h, then stained with diaminobenzidine (DAB) according to manufacturer’s instructions (GBI, USA) and subsequently counterstained with hematoxylin. Images were taken by Vectra Automated Quantitative Pathology Imaging System (Perkin Elmer). The intensity of immunohistochemistry staining was scored as follows: 0, weak as light brown; 1, strong as dark brown or even near black.

### RNA sequencing

RNA was isolated from normal mucosa, adenoma and organoid using RNeasy mini kit (Qiagen) following manufacturer’s instructions. RNA from freshly tissues and organoids was converted into cDNA libraries then subjected to high-throughput sequencing. Over 45 million reads were obtained per sample on Illumina Novaseq 6000. The raw RNA-seq data were filtered using trim-galore (v3.4) by removing reads which contained adapter sequences, low-quality, and short sequences (Phred-Quality score < 20 and read length < 75bps). The high-quality reads were mapped to human reference genome (hg19) by HISAT (v0.1.6-beta) with no more than 2 mismatches, and only the uniquely mapped reads were used for estimating the expression values in gene level by read-count and FPKM. Statistical test of differentially expressed genes was performed by DEseq2 with R. Genes with absolute log2-transformed fold changes greater than 1.5 and a threshold of *p*-value < 0.05 were regarded as differentially expressed genes. Hierarchical clustering of log2-transformed FPKMs was generated by Cluster 3.0 and visualized by Java TreeView.

The transcriptomic profiles between HRCA-PDOs and CRC organoids were further compared with the RNA sequencing results we obtained above and the downloaded datasets. The microarray datasets GSE65253 were downloaded from the Gene Expression Omnibus (GEO) database (http://www.ncbi.nlm.nih.gov/geo). The expression file of GSE65253 was transformed into TPMs (transcripts per kilobase million), which was identical to the RNA sequencing results we generated from the adenoma organoids. The likelihood of batch effects from non-biological technical biases between different datasets was reduced by the “ComBat” algorithm. DEGs were determined by setting significance cutoff criteria to *p*-value < 0.05 (adjusted) and absolute fold-change > 1.5 by employing the limma R package.

### Quantitative real time-poly chain reaction (qRT-PCR)

Total RNA was extracted from organoids using the RNeasy Protect Mini kit (Qiagen) according to the manufacturer’s protocol. RNA was reverse transcribed using Goscript^TM^ reverse transcription System (Promega) according to the manufacturer’s protocol. Each PCR was carried out using SYBR Green Master mix (Vazyme) in the 7500 fast real-time PCR system (Applied Biosystems). Primers were listed in Supplementary Table 2.

### Cell cycle analysis

Organoids treated with metformin/PBS for 48 h were harvested and dissociated into single cells by TrypleE (37 °C, 20 min) digestion and mechanical blow. Cells were then fixed by adding 1 mL of 75% (v/v) ethanol and incubated at 4°C for 60 min. After incubation, cells were washed with PBS and stained with 40 ng/μL propidium iodide (in PBS, containing 5 ng/μL RNase A; Sigma-Aldrich) for 1 h at room temperature. Stained cells were washed, resuspended in 500 μL of PBS, sieved through a 40 μm cell strainer, and analyzed using a BD LSR II flow cytometer (BD Biosciences).

### Immunofluorescence staining and confocal microscopy

For whole mount immunostaining, organoids were fixed in 4% paraformaldehyde for 20 min, permeabilized with 0.1% Triton X-100 for 20 min, and blocked with 5% BSA for an hour at room temperature. Primary antibodies were diluted in blocking buffer and incubated overnight at 4 °C. The following primary antibodies (and their respective dilutions) were used: anti-cleaved-caspase3 (CST, 1:200), anti-Ki67 (CST, 1:200), anti-Cytochrome-C (Abcam, 1:200), anti-PUMA (Abcam, 1:200), anti-PCNA (Abcam, 1:200), and anti-E-cadherin (CST, 1:200). The respective secondary antibodies (Molecular Probes, Invitrogen) were diluted by 1:400 in blocking buffer and incubated at room temperature for an hour. The images of organoids were taken by confocal microscopy (OLYMPUS FV3000 Japan), and the intensity of the fluorescence was quantified by ImageJ software for the corresponding comparisons. ImageJ software was used to quantify the intensity of c-Myc staining of single cells in organoids. After analyzing the intensity of c-Myc immunofluorescence staining of single cells in organoid sections, the average c-Myc fluorescence value in all sample sections was 32.66. Therefore, we set the fluorescence value of 35 as the threshold of strong positive c-Myc and used it to calculate the proportion of strong positive c-Myc expression in each organoid.

### Mutation analysis

DNA was extracted from adenomas and the matched normal tissues and CA organoids using the TIANcombi DNA Lyse&Det PCR Kit (Cat#No: KG203, TIANGEN BIOTECH (BEIJING) CO., LTD., Beijing, China) according to the manufacturer’s protocol. To evaluate the somatic mutations in the four paired adenomas and organoids, the customized TargetSeq® DNA Genomics Capture Kit developed by iGeneTech® (iGeneTech Bioscience Co., Ltd, Beijing, China) was applied to capture targeted genomic DNA followed by post-PCR amplification, quantitative quality control of library, and targeted next-generation sequencing performed by Illumina Novaseq 6000 (Illumina, San Diego, CA, USA). The sequencing results were analyzed and compared for mutation analysis including nonsense mutation, missense mutation, frame shift mutation, and multiple hit for the paired samples.

### Compound library and high-throughput screening

A compound library of small molecular inhibitors and clinically used drugs was obtained from the Selleck. The CA organoids growing in 48-well plate were harvested and resuspended well in ice-cold Matrigel, then dispensed into 96-well plates (Corning 3299 clear black flat-bottom microplates) at 5 μL per well. Inhibitors were added to the culture medium at the final concentration of 0.1 μM, and clinical drugs were added to the culture medium at different final concentrations (Supplementary Table 3). Negative controls including corresponding solvent controls and blank controls were also applied accordingly. Calcein-AM (1 μM), propidium iodide (PI) (5 μg/mL), and Hoechst 33342 (10 μg/mL) were used to identify the live and dead cells and nucleus, respectively. After incubation for 20 min, the images were acquired using High content screening with Z-stacks for each channel.

Prior to drug treatment, CA organoids were plated uniformly and cultured for 5 days. After 3 days of treatment with the compounds at the indicated concentrations, organoids were subjected to CellTiter-Glo 3D Cell Viability Assay (Promega) for detection of luminance (lumi) according to the manufacturer’s instructions. 0.1% dimethyl sulfoxide (DMSO) and 100 μM bortezomib were set as the negative control (NC) and positive control (PC), respectively. When the ratio of the average level of cell viability in the presence of the compounds (*n* = 5) compared to the control (*n* = 5) was under 0.5, the suppressive effect was considered to be significant. Thus, for drug *x* at concentration *y*, viability (*V*) was defined as follows:

$$V_{x,y}=1-\{({\mathrm{lumi}}_{NC}-{\mathrm{lumi}}_{x,y})/({\mathrm{lumi}}_{NC}-{\mathrm{lumi}}_{PC})\}$$

The viability-concentration curve was plotted for each CA organoid and area under curve (AUC) was analyzed. Normalized AUC was calculated uniformly by dividing the AUC of each CA organoid by the maximum AUC in the same group.

### Statistical analysis

Results are presented as mean ± Standard deviation (SD). At least three sets of repeated experiments were performed, and *n*-values indicate the number of primary tissues, PDOs, or animals analyzed in each group. All calculations, curve fitting, and statistical analyses were performed with GraphPad Prism (version 8; GraphPad Software, La Jolla, CA, USA). Statistical significances between/among data sets were analyzed using Student’s *t*-test or one-way or two-way ANOVA followed by the Bonferroni post hoc test. Differences were considered significant at an error probability (*P*) of less than 0.05.

## Results

### Patient-derived high-risk colorectal adenoma organoids (HRCA-PDOs) were generated with Wnt3a-deficient medium system

**Fig. 1:**
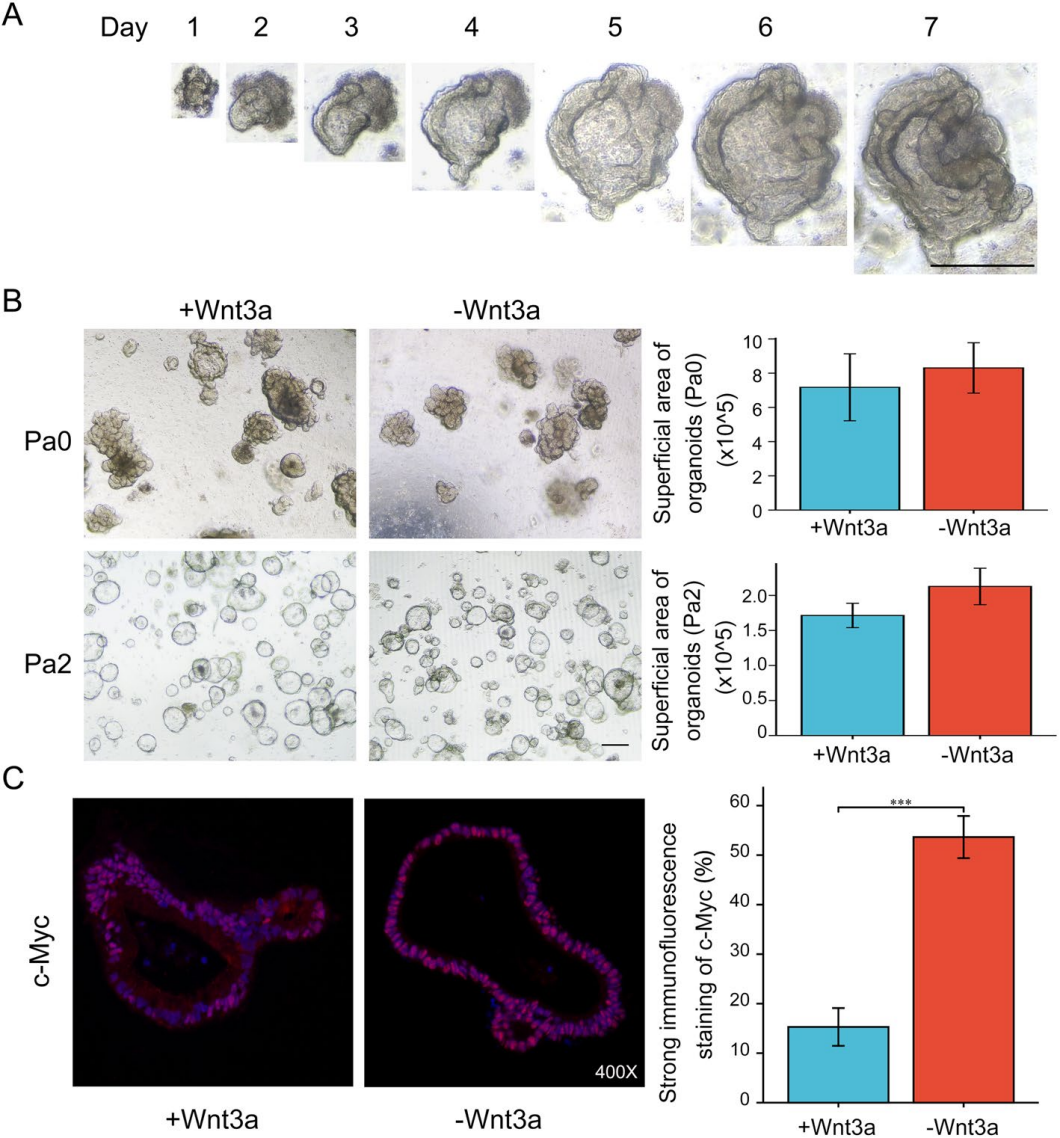
Patient-derived high-risk colorectal adenoma organoids (HRCA-PDOs) were generated with Wnt3a-deficient medium system. **A** Bright-field images showing 7-day culture of CA organoids. Scale bar, 100 μm. **B**, **C** The patient-derived HRCA samples were digested and divided into two parts for organoid culture with the medium with (+Wnt3a) or without Wnt3a (−Wnt3a). The representative pictures of Passage 0 (Pa0) and Passage 2 (Pa2) were demonstrated (**B**, left panel). The superficial area of organoids in more than ten random captures of each group were calculated for analysis (**B**, right panel, *n* = 45). The PDO cultures from Pa2 were obtained for immunofluorescent staining of c-Myc. The representative pictures were displayed. The intensity of red fluorescence was quantified for each single cell and the threshold as strong positive c-Myc staining was set as 35. The ratio of strong positive c-Myc staining for both groups (*n* = 6). Scale bar: 200 μm in **B**, magnification: × 400 in **C**. All data were presented as mean ± SD and subjected to Student’s *t*-test: *** indicates *P* < 0.001 as −Wnt3a versus +Wnt3a

In order to discover potential drugs targeting high-risk colorectal adenoma (HRCA), we explored generating in vitro patient-derived HRCA organoid (HRCA-PDO) models that would be eligible for drug screening. The primary adenomas which were pathologically scored and diagnosed as HRCA by two independent pathologists were collected from patients by endoscopic polypectomy and instantly transferred to the sterile laboratory for organoid culture. The primary adenomas were cleaned, sanitized, and then dissociated into cell clusters by enzyme digestion and lastly embedded in Matrigel for 3D culture (Figure S1). The culture protocol was specially established based on the publication by Sato et al. (2011) [19]. As the initiation of colorectal adenoma and cancer is highly correlated with Wnt hyperactivation while Wnt3a is essential for the maintenance of normal epithelium [24], we were enlightened by the culture methods by van der Wetering et al. who enriched CRC organoids by withdrawing Wnt3a in the culture media [25]. Therefore, we attempted to apply Wnt3a-deficient human colon organoid culture condition (R-spondin1, Noggin, EGF and small molecule inhibitors) to selectively expand HRCA-PDOs [19]. The HRCA-PDOs could be generated within 7 days and maintained for months with unattenuated expanding capacity (Fig. 1A). Meanwhile, the HRCA-PDOs could be frozen to create a master cell bank and the cell survival rate was typically larger than 80% upon thawing. During the primary culture (Passage 0, Pa0), we generally cultured the cell clusters rather than digesting the adenoma tissue into single cells in order to improve the success rate. Organoids were digested into single cells for further expansion and/or purification from Pa0 to Passage 1 (Pa1) and so on, which could lead to the different morphologies of HRCA-PDO in Pa0 and other passages. We have compared the viability of the organoid cultures in media with or without Wnt3a for both Pa0 and Passage 2 (Pa2). The results indicated that deficiency of Wnt3a in culture medium did not affect general organoid growth (Fig. 1B). During passage, we observed significant organoid death in Wnt3a-deficient culture condition. In Pa2, the purity of HRCA-PDO with Wnt3a-deficient culture condition is significantly higher than organoids in culture medium with Wnt3a reflected by distinct expression of c-Myc. By quantifying the intensity of immunofluorescent staining of c-Myc, the results demonstrated that the ratio of cells with strong c-Myc expression in Wnt3a-deficient culture condition is about 52%, which is significantly higher than that in culture medium with Wnt3a which is around 15% (Fig. 1C). Meanwhile, the results of immunohistochemical staining demonstrated that the ratio of strong staining of c-Myc of organoids reached almost 100% in Wnt3a-deficient culture medium versus about 58% in culture medium with Wnt3a (Figure S2). The high purity of HRCA-PDO cultures guaranteed the specificity during drug screening.

### HRCA-PDOs retained the histopathological features, mutational fingerprint and molecular characteristics of primary tissues

**Fig. 2:**
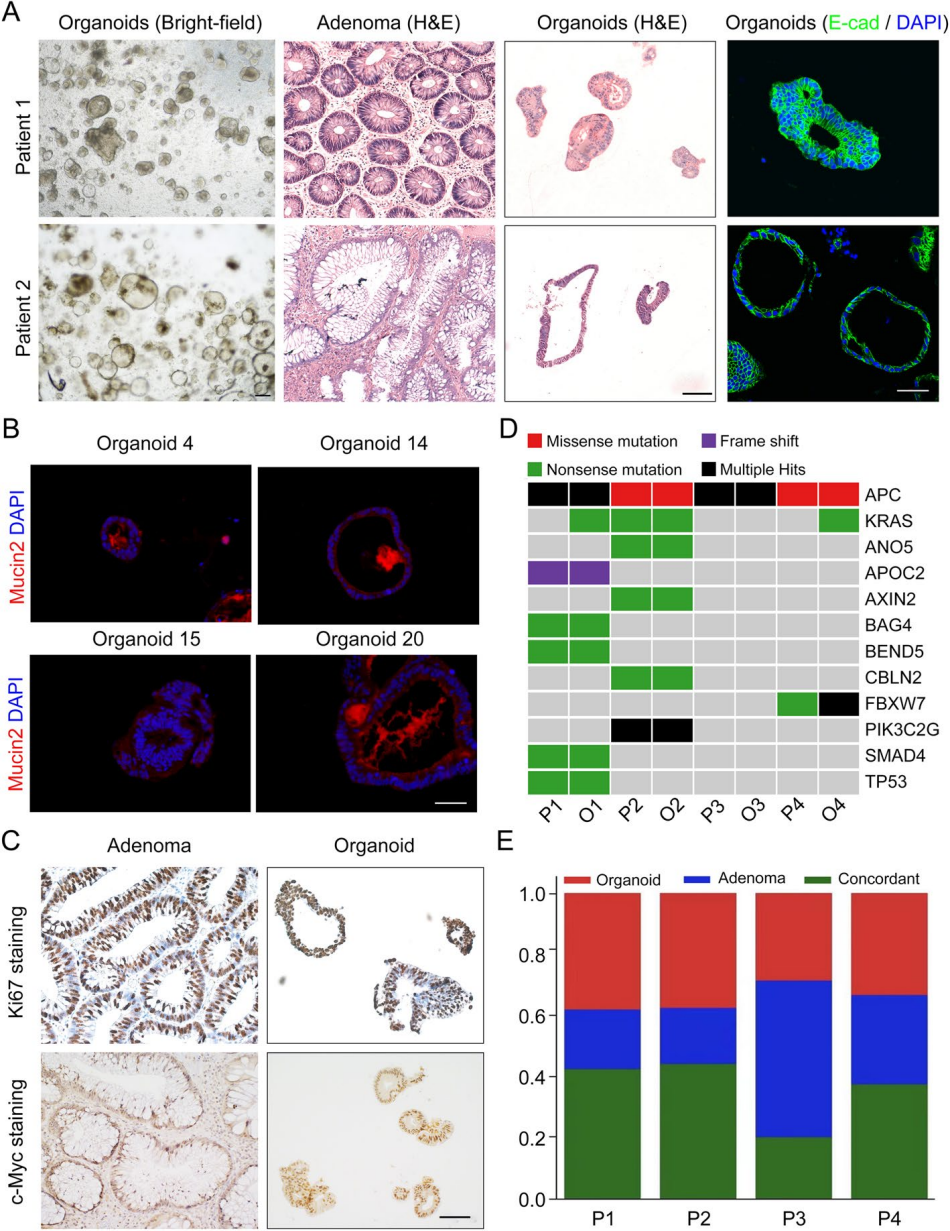
HRCA-PDOs retained the histopathological features, mutational fingerprint and molecular characteristics of primary tissues. **A** H&E staining, bright-field images, and E-cadherin (E-Cad) immunofluorescence staining on primary adenomas and/or organoids from patients 1 and 2. Scale bar, 50 μm. **B** Immunofluorescent staining of mucin2 on HRCA-PDOs from patients 4, 14, 15, and 20 labeled by organoids 4, 14, 15, and 20. Scale bar, 50 μm. **C** Immunohistochemical staining of Ki67 and c-Myc on primary adenomas and HRCA-PDOs. Scale bar, 100 μm. **D** The comparison of mutation landscapes of adenomas from four patients P1–4 (P1, P2, P4, and P5 in the Table S1) and the corresponding HRCA-PDOs 1–4 (O1–O4) were displayed. The type of genetic alteration (noted by color code) was displayed for the commonly mutated genes. **E** Concordances of somatic mutations were analyzed for paired adenomas and HRCA-PDOs. Bar graph represents the proportion of coding alterations that are concordant between the primary adenoma and the corresponding organoid culture and those that are found only in organoid or primary adenoma specimen

We then sought to determine whether these HRCA-PDOs could highly mimic the primary adenoma in various aspects. In regard with the histopathological features, HRCA-PDOs presented individual-specific morphologies, ranging from multiple-layered solid structures to single-layered cystic structures, which generally preserved the organization pattern of the corresponding primary tissues (Fig. 2A). As is shown in Fig. 2B, the representative immunofluorescence staining results demonstrated that mucin2 is commonly expressed in HRCA-PDOs derived from different patients, which indicated that HRAC-PDOs still retained the ability to produce mucin. Subsequent analyses of proliferative and pathological markers by immunohistochemical staining revealed that HRCA-PDO and primary adenoma exhibited similar expression patterns of Ki67 and c-Myc (Fig. 2C and Figure S3).

To determine whether HRCA-PDO also preserved the mutational fingerprint of primary adenomas, we isolated genomic DNA from four pairs of primary adenomas and the corresponding organoids including Patient 1-4 (P1-4)/HRCA-PDO 1-4 (O1-O4) for next generation sequencing to determine the somatic mutations. Patients 1–4 indicated P1, P2, P4, and P5 in Table S1 and annotated as P1–4 in Fig. 2D, E and Figure S4. Comparative analyses of somatic mutations indicated that the common colorectal cancer driver mutations, including *APC*, *Smad4*, and *TP53*, were well represented in culture (Fig. 2D). Notably, *APC* mutation was observed in all cases, which confirmed Wnt activation was the major driving factor of primary adenoma (Fig. 2D). Interestingly, some mutations like KRAS and DNAH3 were detected in organoids but not in primary adenoma, and the multiple hit of FBXW7 was observed in O4, but only missense mutation was detected in P4 (Fig. 2D and Figure S4). When we looked into the global view of the concordance of somatic mutations between corresponding primary adenoma and organoids, we discovered that they shared ~40% mutations, and generally there are more organoid-only than adenoma-only mutations (Fig. 2E). The inconformity could be due to the different ratios of adenoma cells in organoid and corresponding primary tissue biopsy. The organoids are the selectively purified adenoma parts of the primary tissues, while the primary tissues also contain many normal epithelial cells. Therefore, the relative content of certain mutations (like KRAS in P4/O4, DNAH3 in P1/O1, as well as missense mutation of FBXW7 in P4 but multiple hit in O4), could be too low to reach the detection threshold in primary tissues but could be detected in organoids. Also, there could be several mutations specific for normal epithelial cells that are grouped as adenoma-only mutations.

**Fig. 3:**
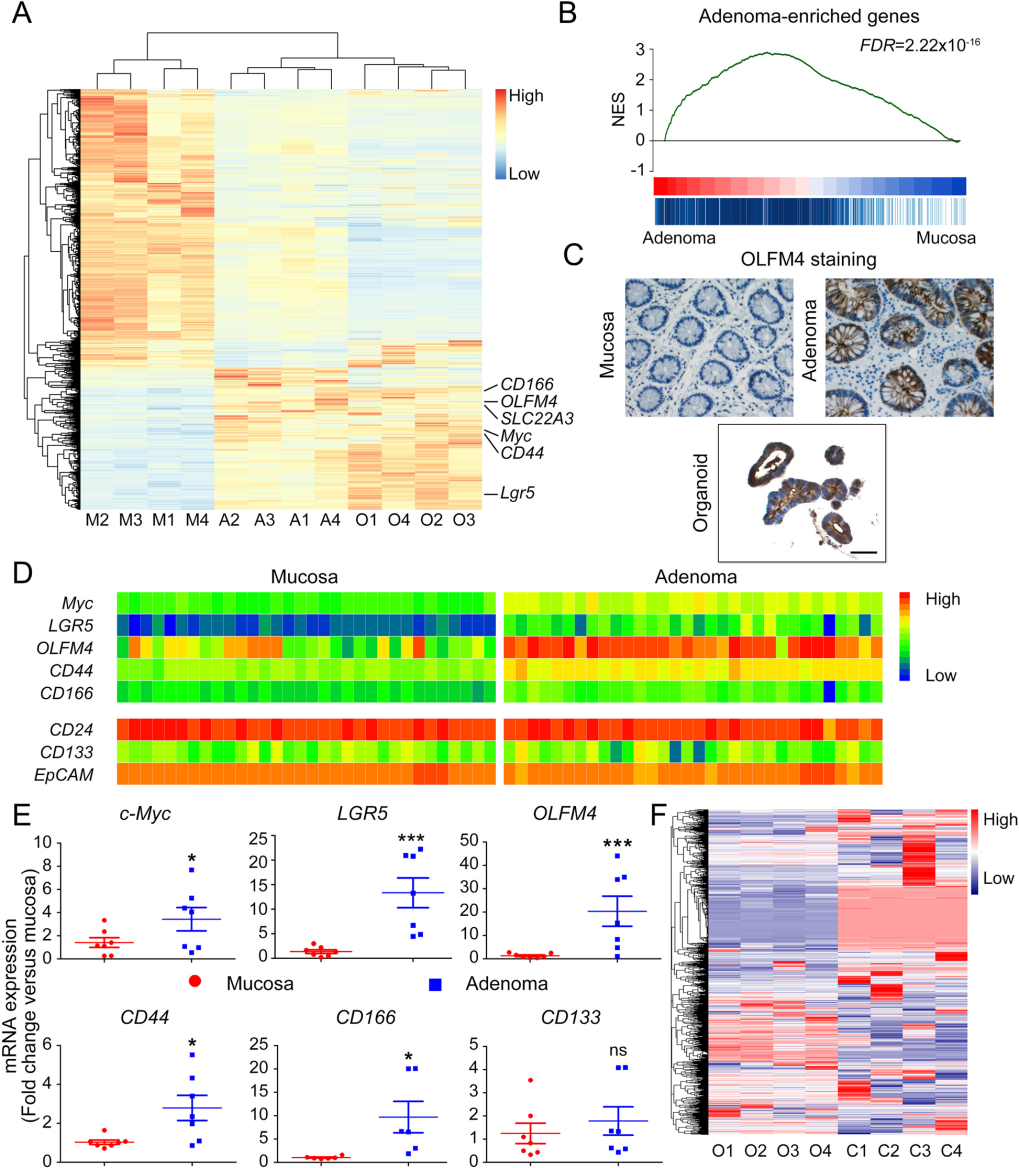
The molecular characteristics of colorectal adenoma are preserved in organoid cultures. **A** Clustered heatmap of log2-transformed RPKMs showed the 3511 differentially expressed genes in adenomas, CA organoids, and normal mucosae originated from four patients (P1, P2, P4, P5). Stem genes were marked. M, mucosa; A, adenoma; O, organoid. **B** Gene set enrichment analysis (GSEA) indicated a gene list containing the upregulated genes in adenoma compared with normal mucosa. *NES*, normalized enrichment score. *FDR*, false discovery rate. **C** Immunohistochemical staining displayed the expression of OLFM4 in CA organoid and corresponding primary adenoma and normal mucosa. A representative result of three independent experiments was shown. Scale bar, 100 μm. **D** Clustered heatmap of log2-transformed RPKMs showed the expression of indicated stem genes between adenoma and normal mucosa. **E** Adenoma and normal mucosa were harvested to verify expression of *c-Myc*, *LGR5*, *OLFM4*, *CD44*, *CD166*– and *CD133* using qRT-PCR. All data were presented as mean ± SD (*n* = 7) and subjected to Student’s *t*-test in **E**. *** indicates *P* < 0.001 versus mucosa. * indicates *P* < 0.05. ns indicates no significant difference. **F** Clustered heatmap of normalized RPKMs showed the 2689 differentially expressed genes in CA organoids originated from four patients (P1, P2, P4, P5) and four representative colorectal adenocarcinoma organoids labeled as P17.2, P19b.1, P20.11, and P31.12 from GSE65253. O, CA organoids; C, colorectal adenocarcinoma organoids

Although the molecular signatures of CRC have been extensively studied, the gene expression pattern of adenoma is less characterized [26]. In this study, we subjected primary adenomas, paired normal mucosa tissues, and corresponding CA organoids to RNA-sequencing to investigate their transcriptomic signatures. We addressed 3511 differentially expressed genes (DEGs) by comparing primary adenomas to paired normal mucosa tissues. Remarkably, the expression pattern of these DEGs in CA organoids was highly similar to the primary adenomas and significantly distinct from the normal mucosa (Fig. 3A). Gene set enrichment analysis (GSEA) further confirmed that CA organoids globally preserved the molecular characteristics of primary adenoma (Fig. 3B).

Interestingly, we noticed that when compared to normal mucosa, both primary adenoma and CA organoids demonstrated significantly higher expression of several colon stem cell signature genes [27, 28] including *OLFM4*, *LGR5*, *c-Myc*, and *CD166* (Fig. 3A). Meanwhile, the immunohistochemical staining results confirmed that OLFM4 expression was dramatically upregulated in primary CA and corresponding PDO culture as compared to mucosa (Fig. 3C).

To determine whether these genes act as universal CA markers, we analyzed the RNA-sequencing data of adenoma and paired mucosa of 32 patients in the GEO database (GSE8671). Gene expression heatmap revealed significant enrichment of *LGR5*, *OLFM4*, *c-Myc*, *CD44*, and *CD166* in adenoma (Fig. 3D), which is consistent with the results of Fig. 3A and further validated by qRT-PCR results tested with the paired samples of seven patients (Fig. 3E). Of note, some well-established carcinoma markers, including *CD24*, *CD133*, and *EpCAM*, were not upregulated in adenoma as compared to mucosa (Fig. 3D and E). These data suggested that adenoma stayed in a mucosa-to-carcinoma transiting stage.

Based on the gene expression profiles in Fig. 3A, we also performed the Gene Ontology (GO) and KEGG pathway analyses between mucosa versus adenoma, and organoid versus adenoma (Figure S5). The results showed that compared to normal mucosa, functions including leukocyte migration, leukocyte chemotaxis, and collagen-containing extracellular matrix were significantly downregulated in adenoma. On the other hand, gland development, cell cycle, regulation of fibroblast proliferation, and the p53 signaling pathways were remarkably upregulated. These results are consistent with the decrease in immune cell infiltration and deposition of normal extracellular matrix as well as an increase in gland proliferation and activation of the P53 pathway during the development of adenomas (Figure S5A, B). Furthermore, we observed the significantly downregulated pathways including the microvilli structure and IgA secretion of intestinal immune cells in adenoma as compared to mucosa (Figure S5C, D). Overall, our GO and KEGG analysis provided insights into the mechanisms involved in the pathogenesis of HRCA.

Meanwhile, we compared the transcriptomic profiles between adenoma and the corresponding organoids. Due to the distinct growth condition of adenoma epithelial cells in vivo and in vitro, transcriptomic gene expression profiles in organoids are different from that in primary adenomas. Sufficient activators of EGF and Wnt pathways in culture medium resulted in functional enrichment of mitotic nuclear division, nuclear division chromosome segregation, and cell cycle in organoid transcriptome, which is in accord with the significantly higher growth rate in vitro than that in vivo. Moreover, adenoma tissue contains immune cells, fibroblasts, and connective tissue, leading to the enrichment of chemokines and extracellular matrix in adenomas compared to organoids (Figure S5E, F).

To further verify the uniqueness of the HRCA-PDOs that we generated, we normalized and compared the RNA-sequencing results of colorectal adenocarcinoma organoids originated from the GEO database (GSE65253) [25] to the mucosa, adenoma, and organoids in our study. The heatmap results indicated that the global gene expression patterns of adenocarcinoma organoids are obviously distinct from adenoma organoids (Fig. 3F and Figure S6A). The gene expression of CA markers including *c-Myc*, *LGR5*, *OLFM4*, *CD44*, *CD166*, and CD133 were analyzed separately (Figure S6B, C). The expression of c-Myc is significantly higher in adenocarcinoma organoids than in adenoma organoids while the expression of the *LGR5* and *OLFM4* in adenocarcinoma organoids is extremely low. Meanwhile, the expressions of *CD44*, *CD166*, and *CD133* are comparable between adenocarcinoma and adenoma organoids. Furthermore, we analyzed the DEGs and conducted GO analyses (Figure S6D, E). The results revealed a set of 194 DEGs (117 downregulated and 77 upregulated, *P*_*adj*_ < 0.05) in adenocarcinoma organoids versus adenoma organoids (Figure S6D). Based on the DEGs, we performed the GO analysis to determine the significantly changed molecular functions (MF), cellular component (CC) as well as biological process (BP) (Figure S6E).

To summarize, these results demonstrated that HRCA-PDOs recapitulated the histopathological features and mutational fingerprints, as well as molecular characteristics of the primary adenoma.

### HRCA-PDO-based high-throughput/content screening platform has been established for colorectal adenoma drug discovery

In order to guarantee sample heterogeneity for drug screening, we attempted 47 HRCA organoids derivation from 40 individual patients and successfully generated a HRCA-PDO biobank comprising of 37 lines from 33 patients. All clinical samples were diagnosed as HRCA by two pathologists independently. The overall success rate is 78.7% (37/47) (Table S1).

**Fig. 4:**
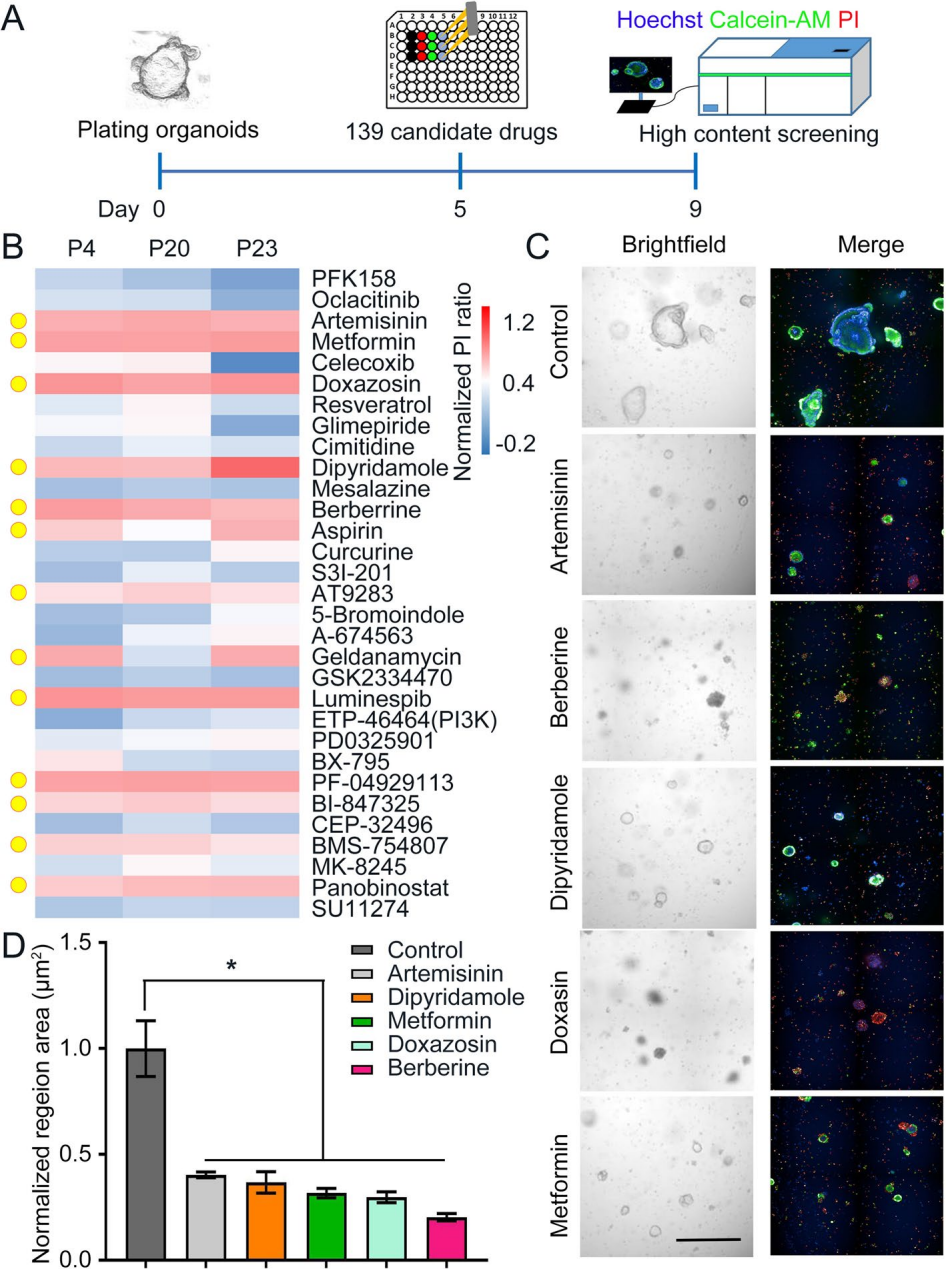
HRCA-PDO-based high-throughput/content screening platform has been established for colorectal adenoma drug discovery. **A** Workflow of high-throughput/content drug screening. HRCA-PDOs from the established biobank were passaged and plated in 96-well plates for 5-day culture, then treated with candidate drugs for four days before staining with PI (red), Hoechst (blue) and Calcein-AM (green), followed by high-content imaging. **B** Heatmap analysis of the normalized PI/Hoechst ratio in organoids after drug treatment among three HRCA-PDO lines derived from three different patients. Colors range from blue (low mortality) to red (high mortality). P, patient. Thirteen top hits were highlighted with yellow circles. **C** Bright and fluorescent field imaging illustrated suppressive effect of indicated drugs (artemisinin, berberine, dipyridamole, metformin, and doxazosin) compared with control on HRCA-PDOs. Scale bar, 200 μm. **D** The normalized surface area of organoids in control and drug-treated groups were calculated and compared. All data were presented as dot plot with mean ± SD (*n* = 4) and subjected to one-way ANOVA followed by the Bonferroni post hoc test. in D. * indicates *P* < 0.05 versus control group

Based on this biobank, we developed a high-throughput and high content HRCA drug screening platform. Three HRCA-PDO cultures (P4 (tubular adenoma), P20 (villous tubular adenoma), and P23 (serrated adenoma)) were passaged and plated in droplet in 96-well plates (the detailed information of patients was listed in Table S1). We incorporated a number of controls into the assay design. After 5-day routine culture, the organoids were treated with drug compounds for 4 days followed by assessing cell viability with Calcein-AM and propidium iodide (PI) staining and high content assay detection (Fig. 4A). When the ratio of PI positive staining after the treatment of the compounds versus Hoechst was larger than 40% as normalized to the control (0.1% DMSO), the suppressive effect was considered to be significant (Fig. 4B).

A customized 139 compound library was assembled for screening (Figure S7, Table S3). Nineteen compounds are FDA-approved CA-related drugs (Figure S7). The rest 120 drugs are previously or currently being investigated in clinical trials with no significant reported side effects, targeting specific signaling pathways that are crucial for CA formation, including Wnt/β-catenin, TGF-β/SMAD, JAK/STAT, MAPK, and DNA mismatch repair.

Presented by sensitivity heatmap, the primary screening results showed that most compounds exhibited no suppressing effects and demonstrated inconsistent efficacy in different patient-derived CA organoid lines (Figure S7A). Among those 139 drugs, we successfully screened out top 13 hits that consistently suppressed CA organoids growth (Fig. 4B, highlighted with yellow circles). Specifically, the 13 compounds could be classified into: JAK/STAT inhibitor (AT9283), HDAC inhibitor (panobinostat), HSP90 inhibitors (geldanamycin, luminespib, and PF-04929113), protein tyrosine kinase inhibitor (BMS-754807), metabolic agents (metformin and dipyridamole), anti-microbiology agents (berberine and artemisinin), MAPK inhibitor (BI-847325), neuronal signaling inhibitor (doxazosin mesylate), and cyclooxygenase inhibitor (aspirin). Apart from PI detection (dead cell, red), the high content assay also provided simultaneous imaging of Calcein-AM (live cell, green) (Fig. 4C) and organoid area (Fig. 4D), which further confirmed the screening results.

**Fig. 5:**
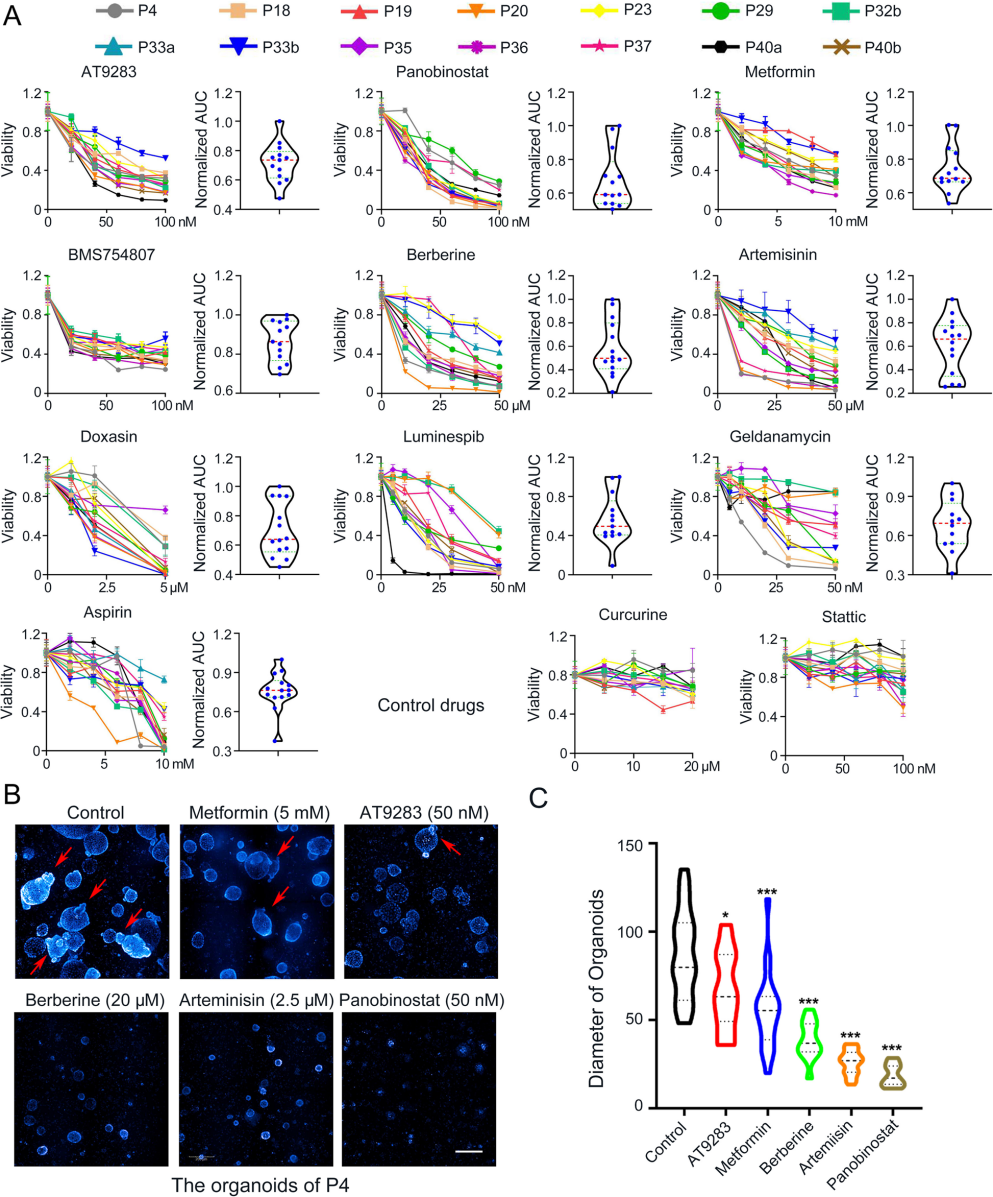
Further drug screening suggested metformin, BMS754807, panobinostat and AT9283 as four top hits with common suppressive efficacy on HRCA-PDO growth. **A** Chemosensitivities of 12 compounds (including ten potential hits and two control drugs) to 14 HRCA-PDO lines were evaluated in the form of dose-response curves. AUC was calculated from the raw dose–response curves, normalized and displayed as violin plot; the red dashed line and green dashed lines represent the mean and upper and lower quartiles, respectively. All data were presented as mean ± SD (*n* = 3). **B** Z-stacks screening demonstrated the effects of the tested drugs on the size and budding of organoids (P4). White scale bar, 200 μm. The red arrows indicate buddings. **C** The diameters of the organoids with different treatments were analyzed by Image J for comparisons. All data were presented as mean ± SD (*n* = 20-34) and subjected to one-way ANOVA followed by the Bonferroni post hoc test. * indicates *P* < 0.05 and *** indicates *P* < 0.001 versus control group

To further confirm that the top hits could extensively exert significant suppressive effects on HRCA-PDO growth and compare the efficacy, we assessed the post-treatment cell viability with a wider range of 14 HRCA-PDO lines derived from 12 patients established in the present study (Table S1). The drugs we tested comprised of ten potential hits including AT9283, panobinostat, metformin, BMS-754807, berberine, artemisinin, doxazosin mesylate, luminespib, geldanamycin, and aspirin. Meanwhile, we took Curcurine and Stattic as two negative controls as they are marketed drugs with no obvious side effects and demonstrated no suppressing effects on HRCA-PDOs in the primary screening (Fig. 5A and Figure S7A). Displayed as dose-response curves and area under curve (AUC), the 14 HRCA-PDO lines showed diverse spectrums of dose-dependent response across these tested drugs (Fig. 5A and Figure S8). The results demonstrated that metformin, BMS754807, panobinostat, and AT9283 displayed generally consistent dose-dependent suppressive effects on all 14 HRCA-PDO lines (Fig. 5A). In accordance with cell viability assay, Z-stack scanning results also demonstrated that these drugs significantly reduced the volume of organoids and inhibited budding with optimal concentrations (Fig. 5B and C).

Altogether, based on the HRCA-PDO biobank, we developed a high-throughput and high-content drug screening platform and discovered metformin, BMS754807, panobinostat, and AT9283 as the four top hits with generally common suppressive efficacy on HRCA-PDO growth.

### Metformin inhibited HRCA growth by restricting stemness maintenance in vitro and in vivo

As metformin is an FDA-approved drug with widely accepted safety profiles even in long-term use, we chose to further investigate into metformin on its potential efficacy and underlying mechanisms regarding CA inhibition from the top four hits (metformin, BMS754807, panobinostat, and AT9283) [15].

**Fig. 6:**
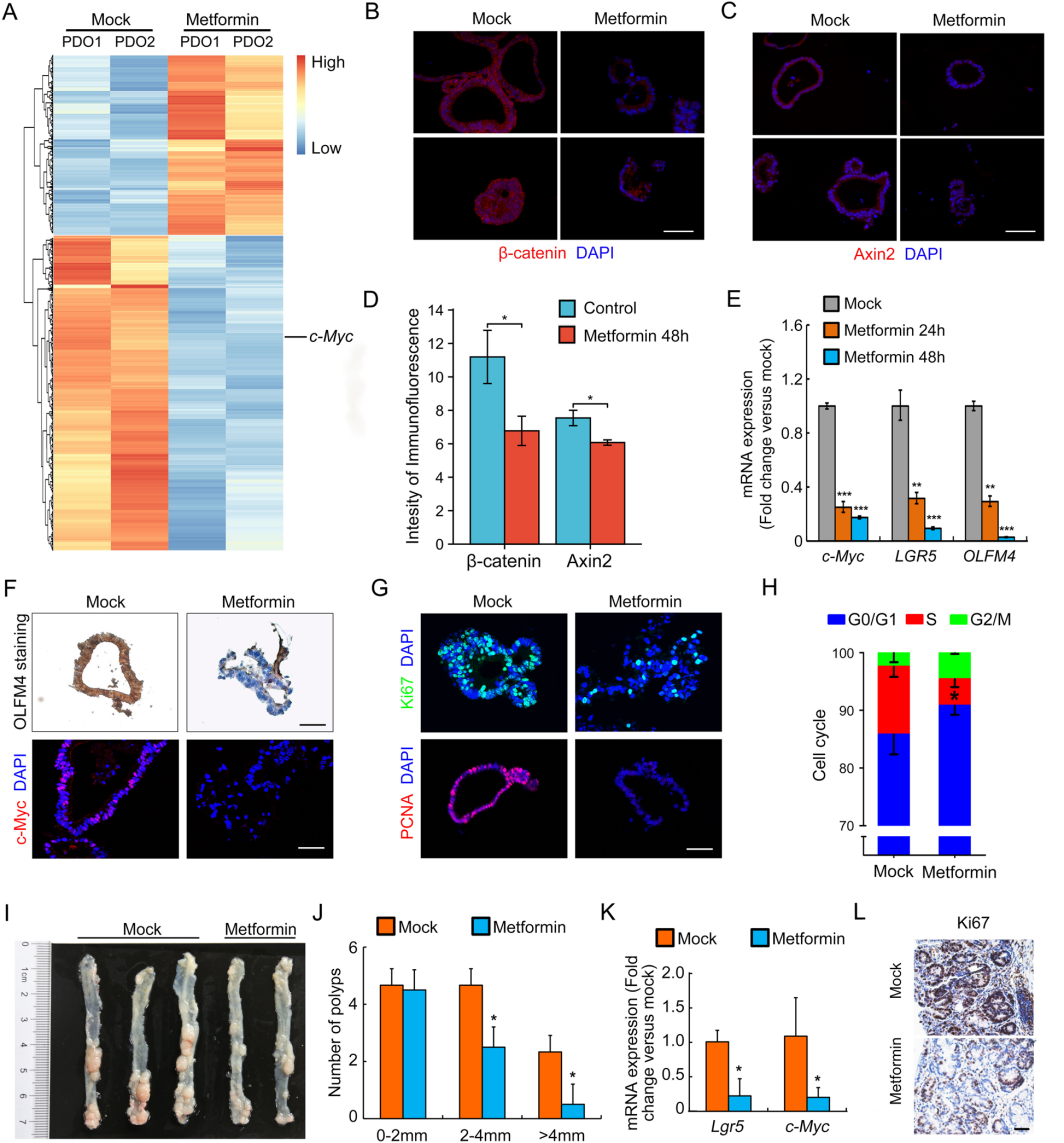
Metformin inhibited HRCA growth by restricting stemness maintenance in vitro and in vivo. **A** Clustered heatmap of log2-transformed RPKMs showed the differentially expressed genes (DEGs) after metformin treatment (5 mM, 24 h). The organoids treated with metformin were cultured from P4 and P8. c-Myc was marked. **B**, **C** Immunofluorescent staining of β-catenin and Axin2 on organoids in metformin-treated (48h) and mock groups. Scale bar, 50 μm. (right panel). **D** Quantification of fluorescence intensity in organoids was performed and analyzed for β-catenin and Axin2. **E** After metformin treatment for 24 and 48 h, HRCA-PDOs were harvested to perform qRT-PCR for evaluation of the mRNA expression of LGR5, OLFM4 and c-Myc. **F** Expression of OLFM4 and c-Myc in HRCA-PDOs after metformin treatment for 48 h were detected by immunohistochemical staining and immunofluorescent staining, respectively. Scale bar, 50 μm. (right panel). **G** HRCA-PDOs were harvested to examine the expression of proliferation markers including Ki67 and PCNA by immunofluorescence staining after metformin treatment for 48 h. A representative result of three independent experiments was shown. Scale bar, 50 μm. **H** FACs analysis revealed an obvious cell cycle change of adenoma cells after metformin treatment (5 mM, 48 h). FACs, fluorescence-activated cell sorting. **I**, **J** Bright-field image showed the colorectal tumor formation in colon between mock and metformin-treated groups (left panel) and quantification of colorectal tumors in mice (*n* = 5 in control group and *n* = 4 in metformin-treated group) were counted and statistically analyzed (right panel). **K**–**L** qRT-PCR examination of expression of *Lgr5* and *c-Myc* mRNA levels and immunohistochemical staining of proliferative marker Ki67 in colorectal tumors between mock and metformin-treated groups. All data were presented as mean ± SD (*n* = 4-5) and subjected to one-way ANOVA by the Bonferroni post hoc test in **E**, Two-way ANOVA followed by the Bonferroni post hoc test in **H** and **J** and Student’s t-test in **D** and **F**. * indicates *P* < 0.05, ** indicates *P* < 0.01, and *** indicates *P* < 0.001 versus mock group

To elaborate the mechanism of how metformin inhibited PDO growth, we examined the principal transcriptomic changes of HRCA-PDOs upon metformin treatment for 24 h. RNA-sequencing results revealed a set of 962 DEGs (613 downregulated and 349 upregulated, *P* < 0.05) in metformin-treated HRCA-PDOs (Fig. 6A). Based on DEGs, we performed the GO analysis to determine the significantly changed MF, cellular CC, and BP between metformin-treated and control HRCA-PDOs (Figure S9). As stemness gene *c-Myc* was observed to be significantly downregulated in metformin-treated PDOs (Fig. 6A), we further confirmed the decreased expression of stemness genes *LGR5*, *OLFM4*, and *c-Myc* by qRT-PCR after metformin treatment (Fig. 6E, left), even before CA organoids decay in morphology (Figure S10). Besides, β-catenin and Wnt target gene Axin2 ware also significantly downregulated after metformin treatment for 48 h (Fig. 6B, D). Consistently, immunohistological and immunofluorescent staining further supported that metformin treatment for 48 h efficiently ablated the expression of OLFM4 and c-Myc, respectively (Fig. 6F). These results suggested that metformin treatment abolished the stem cell-like identity of adenoma cells in HRCA-PDOs. As stem genes is highly correlated with cell proliferation, we further unveiled that metformin-treated HRCA-PDOs displayed dramatically decreased expression of fast proliferation makers Ki67 and PCNA (Fig. 6G). Moreover, fluorescence-activated cell sorting (FACs) analysis revealed that metformin induced an obvious cell cycle arrest of adenoma cells (Fig. 6H). The significantly elevated expression of PUMA and cytochrome C and increased cleavage of caspase 3 indicated that the adenoma cells in HRCA-PDOs underwent apoptosis following metformin treatment (Figure S11).

Apart from stemness maintenance, treatment of metformin on CA organoids lead to intensive transcriptional suppression of genes involved in “cell cycle,” “apoptosis,” “glycolysis,” and “p53 signaling pathway” (Figure S12A, B). qRT-PCR further verified that the expression of glucose transporters *GLUT1* and *GLUT3* as well as glycolytic enzymes *LDHA*, *PKM2*, *PGK1*, and *PGM1* was significantly downregulated upon metformin treatment (Figure S12C, D).

Consistent with our study, several clinical reports indicated that metformin could significantly reduce the incidence and recurrence rate of polyps in diabetic patients [16]. Here, we would like to evaluate the therapeutic effect of metformin on CA in vivo. We generated the classical adenoma-to-cancer mouse model by administrating AOM/DSS on 6-week-old male C57BL/6J mice [29]. AOM injection followed by two rounds of DSS feeding led to formation of abundant adenomas (5 weeks post AOM injection), which then developed into colorectal tumors (10 weeks post AOM injection) (data not shown). Daily administration of metformin for 3 weeks enabled the mice to recovered from colitis and slightly gained body weight (Figure S13), indicating the safety of metformin from gastrointestinal side effects. Metformin treatment efficiently attenuated the CA development, represented by dramatically decreased number of sized colorectal tumors/adenomas (Fig. 6I, J).

We then examined the molecular changes in AOM/DSS-induced colorectal tumors/adenomas following metformin therapy. Consistent with the effects on HRCA-PDOs, metformin treatment led to significantly decreased mRNA expression of adenoma stemness markers *Lgr5* and *c-Myc* (Fig. 6K, left) and fast proliferation marker Ki67 (Fig. 6L, right), confirming that metformin prevented CA development through restricting the stemness maintenance of AOM/DSS-induced adenoma.

## Discussion

Although there are several publications that mentioned the culture method of CA organoids [19] or the establishment of CRC organoid biobank [20, 21, 25], there has not been any systematic and comprehensive studies focused on the CA organoids, let alone the establishment of large-scale patient-derived CA organoid biobank that could be available for drug screening. Therefore, in order to explore the potential pharmaceutical treatment of HRCA, we developed a promising HRCA-PDO culture method and established a biobank comprised of 37 lines derived from 33 patients in a Chinese cohort. Based on this HRCA-PDO biobank, we conducted the first high-throughput and high-content drug screening and screened out 13 potential hits that could effectively inhibit HRCA growth or development. Especially, we addressed metformin as an effective HRCA-targeted drug probably through restricting the stemness maintenance of HRCA both in vitro and in vivo.

We applied non-Wnt3a culture condition which significantly enhanced the purity of HRCA-PDO to meet the criteria for drug screening. Meanwhile, the probability to detect the accurate genetic mutations is higher with purified organoid cultures than primary adenomas (Figure S4), which could benefit patient diagnosis. Moreover, by generally retaining the histological, transcriptomic, and genomic landscape of primary adenoma, HRCA-PDOs provided a novel platform for adenoma research, including biomarker discovery, drug screening, and mechanistic study.

Based on the current knowledge of CA formation and clinical treatment, we designed a drug library for a proof-of-concept drug screening, including inhibitors of specific signaling pathways crucial for CA formation [8, 30–32] (Wnt/β-catenin, TGF-β/SMAD, JAK/STAT, and MAPK) and clinical drugs (metformin, berberine, and doxazosin). Using high-content screening assay, metformin and its 12 peers were screened out as the potential anti-HRCA hits. We noticed that the efficacy of treatment by luminespib, geldanamycin, berberine, aspirin, and artemisinin varied a lot among different HRCA-PDO lines, indicating the heterogeneity among different patients, highlighting the merits of HRCA-PDO applications for personalized medicine. Interestingly, autologous organoids derived from different positions in colon showed distinct responses to drug treatment. For instance, P40a was sensitive to luminespib, while P40b was sensitive to geldanamycin. This revealed various heterogeneity of mutation and sensitivity of separate adenomas even in the same patient. Interestingly, those inhibitors targeting Wnt/β-catenin and TGF-β/SMAD were proved to be ineffective on HRCA-PDOs (Figure S6A), indicating that mere perturbation of Wnt or TGF-β signaling may not be effective for HRCA patients. By contrast, drugs with broad spectrum, such as metformin and panobinostat, displayed excellent efficacy in the polit screening and intensive testing by biobank (Figs. 4 and 5). In addition, the selection of appropriate positive and negative controls is critical for the promising design of drug screening assays. Inclusion of multi-dimensional negative controls should be seriously paid more attention to improve the future study of organoid-based drug screening.

Stem cells are responsible for normal tissue renewal or the regeneration after damage. Cancer stem cells play an important role in tumor initiation, proliferation, metastasis, and recurrence [1]. HRCA showed remarkably higher expression levels of intestinal stem cell markers (LGR5, OLFM4) and cancer stem cell markers (c-Myc, CD166) (Fig. 3). Lgr5+ stem cells can form gradually proliferating adenomas on abnormal Wnt pathway activation, indicating that Lgr5+ cells were the cells-of-origin of colorectal tumors [33]. OLFM4 was closely associated with digestive diseases, whose dysregulation had been detected in gastrointestinal malignancies, including gastric cancer, colorectal cancer, pancreatic cancer, and gallbladder cancer [34]. With the hyperactivation of Wnt/β-catenin pathway, the downstream oncogene c-Myc was overexpressed, which promoted the occurrence and development of colorectal tumors [35]. CD166 was expressed high in colon cancer, demonstrated as a cell surface marker for identification of colorectal cancer stem cells [36]. Upregulated stem cell markers may drive the initiation and progression of HRCA, which served as promising diagnostic and prognostic biomarkers and can be used for developing targeted therapies for HRCA. In our study, we further compared the gene expression profiles between HRCA organoids and adenocarcinoma organoids (Fig. 3F and Figure S6), which indicated our HRCA-PDO biobank is distinct from the previously reported CRC organoid biobank. Especially, LGR5 and OLFM4 could be the potential biomarkers for the diagnosis of HRCA and prediction of HRCA progression to CRC.

Metformin is lipophilic biguanide as the first-line pharmacologic treatment for type 2 diabetes (T2D) due to its safety, efficacy, and tolerability [15]. In recent years, metformin has been widely tested in clinical practice to exert its therapeutical effects including anti-tumor [37, 38], anti-inflammation [39], and anti-aging [40] as well as regulating gut microbiota [41], displaying its great application value and prospects. Lately, the correlation between metformin and incidence of HRCA and CRC has been investigated in clinical trials. Metformin therapy is associated with a significantly decrease in the risk of incidence and recurrence of CA in T2D patients [42, 43]. Moreover, metformin usage significantly reduced CA as well as CRC incidence and prognosis in non-diabetic patients [16, 44, 45]. However, there has been no preclinical study on the HRCA-related chemoprevention effects of metformin to mechanistically support the clinical results. According to the drug screening results based on the HRCA-PDO biobank we established, metformin demonstrated consistently inhibitory effects on the tested HRCA-PDO lines by restricting the maintenance of stemness in HRCA. Other potential mechanisms regarding the benefits of metformin on HRCA will be investigated further in our future studies.

## Conclusions

Collectively, by generating a large-scale HRCA-PDO biobank, we identified stemness signature genes that can be used as biomarkers for the diagnosis and prognosis of high-risk adenomas. The high-throughput and high-content drug screening with HRCA-PDO biobank enabled us to screen out the potential chemotherapeutical agents targeting HRCA especially metformin, an FDA-proved drug with satisfactory clinical safety, compliance, and cost-effectiveness.

### Supplementary Information

**Additional file 1: Fig. S1.** Culture and analysis workflow of HRCA-PDOs. **Fig. S2.** The HRCA-PDOs were purified in Wnt3a-deficient culture condition. **Fig. S3.** Characterization of HRCA-PDO biobank. **Fig. S4.** Mutation landscapes of corresponding primary adenoma and organoids. **Fig. S5.** GO and KEGG analyses based on DEGs. **Fig. S6.** Comparison of gene expression profiles between colorectal adenoma organoids and adenocarcinoma organoids. **Fig. S7.** Primary high-throughput screening based on HRCA-PDO biobank. **Fig. S8.** Dose-dependent effects of drugs on morphology of HRCA-PDOs. **Fig. S9.** GO analysis of the DEGs between metformin-treated and control HRCA-PDOs. **Fig. S10.** Morphology decay of HRCA-PDOs after metformin treatment. **Fig. S11.** Metformin treatment promoted apoptosis in HRCA-PDOs. **Fig. S12.** Metformin regulated the glycolysis of colorectal adenoma. **Fig. S13.** Body weight changes in mice during metformin intervention. **Table S1.** Biobank list of HRCA-PDOs. **Table S2.** Primers for RT-qPCR. **Table S3.** Concentration of drugs in compound library.

**Additional file 2. **Detailed list of somatic mutations in primary high-risk colorectal adenomas and corresponding organoids.

## Data Availability

All data associated with this study were present in the paper or the additional files. All datasets or information generated in this study are available upon reasonable request from the corresponding authors.

## Abbreviations

LGR5: Leucine-rich repeat-containing G-protein coupled receptor 5
OLFM4: Olfactomedin 4
CD166: Activated leukocyte cell adhesion molecule
HDAC: Histone deacetylase
JAK/STAT: Janus kinase/signal transducer and activator of transcription
Wnt: Wingless-type MMTV integration site family
Ras: Resistance to audiogenic seizures
TGF-β: Transforming growth factor-β
AOM: Azoxymethane
DSS: Dextran sodium sulfate
EGF: Epidermal growth factor
APC: Adenomatous polyposis coli
Smad4: Drosophila mothers against decapentaplegic
TP53: Tumor protein p53
EpCAM: Epithelial cell adhesion molecule
MAPK: Mitogen-activated protein kinase
PCNA: Proliferating cell nuclear antigen
PUMA: p53 upregulated modulator of apoptosis
