# Supplementary material for: Establishment of a large-scale patient-derived high-risk colorectal adenoma organoid biobank for high-throughput and high-content drug screening
Source: BMC Med. 2023 Sep 4;21:336. doi: 10.1186/s12916-023-03034-y (PMC10478412; doi:10.1186/s12916-023-03034-y)
Supplement: Supplementary file 1 — Additional file 1: Fig. S1. Culture and analysis workflow of HRCA-PDOs. Fig. S2. The HRCA-PDOs were purified in Wnt3a-deficient culture condition. Fig. S3. Characterization of HRCA-PDO biobank. Fig. S4. Mutation landscapes of corresponding primary adenoma and organoids. Fig. S5. GO and KEGG analyses based on DEGs. Fig. S6. Comparison of gene expression profiles between colorectal adenoma organoids and adenocarcinoma organoids. Fig. S7. Primary high-throughput screening based on HRCA-PDO biobank. Fig. S8. Dose-dependent effects of drugs on morphology of HRCA-PDOs. Fig. S9. GO analysis of the DEGs between metformin-treated and control HRCA-PDOs. Fig. S10. Morphology decay of HRCA-PDOs after metformin treatment. Fig. S11. Metformin treatment promoted apoptosis in HRCA-PDOs. Fig. S12. Metformin regulated the glycolysis of colorectal adenoma. Fig. S13. Body weight changes in mice during metformin intervention. Table S1. Biobank list of HRCA-PDOs. Table S2. Primers for RT-qPCR. Table S3. Concentration of drugs in compound library. [file 12916_2023_3034_MOESM1_ESM.pdf]

Supplementary materials

Establishment of a large-scale Patient-derived High-Risk Colorectal Adenoma Organoid biobank for high-throughput and high-content drug screening

Zhongguang Luo[1,5], Bangting Wang[1,5], Feifei Luo[1,5], Yumeng Guo[1,5], Ning Jiang[2], Jinsong Wei[2], Xin Wang[2], Yujen Tseng[1], Jian Chen[1], Bing Zhao[2,4*], and Jie Liu[1,3*]

[1]Department of Digestive Diseases, Huashan Hospital, Fudan University, Shanghai 200040, China;

[2]State Key Laboratory of Genetic Engineering, School of Life Sciences, Zhongshan Hospital, Fudan University, Shanghai 200438, China;

[3]Institute of Biomedical Sciences and Department of Immunology, School of Basic Medical Sciences, Fudan University, Shanghai, 200032, China;

[4]Institute of Organoid Technology, Kunming Medical University, Kunming 650500, China.

[5]These authors contributed equally to this work.

*Correspondence: bingzhao@fudan.edu.cn (B.Z.), jieliu@fudan.edu.cn (J.L.)

Supplemental Figures

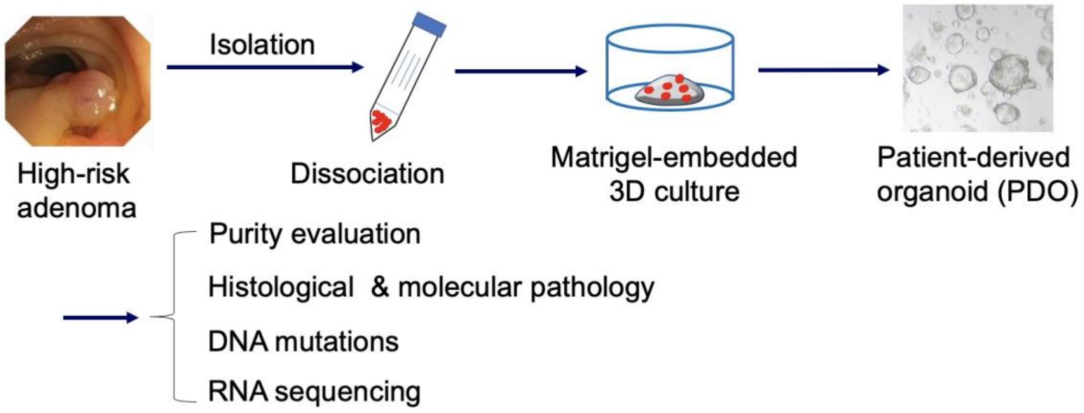

Figure S1. Workflow of culture of patient-derived high-risk colorectal adenoma organoids HRCA-PDOs.

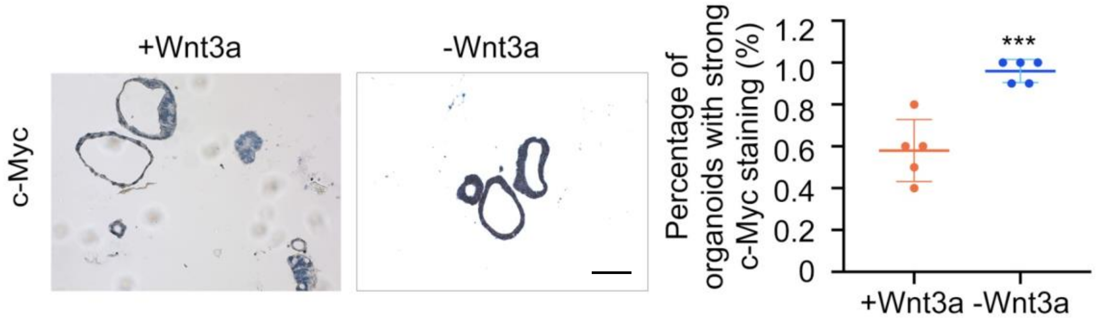

Figure S2. The HRCA-PDOs were purified in Wnt3a-deficient culture condition. The patient-derived HRCA samples were digested and divided into two parts for organoid culture with the medium with (+Wnt3a) or without Wnt3a (-Wnt3a). The PDO cultures from Pa2 were obtained for immunohistochemical staining of c-Myc. The representative pictures were displayed. For weak c-Myc staining as light brown, we score it as 0 and for strong c-Myc staining as dark brown or even near black, we score it as 1. Five sets of experiments were performed and the percentage of organoids with strong c-Myc staining .was calculated for analysis (C, n=5). Scale bar: 100 μm. All data were presented as mean ± SD and subjected to Student's *t*-test: *** indicates $P < 0.001$ as -Wnt3a versus +Wnt3a.

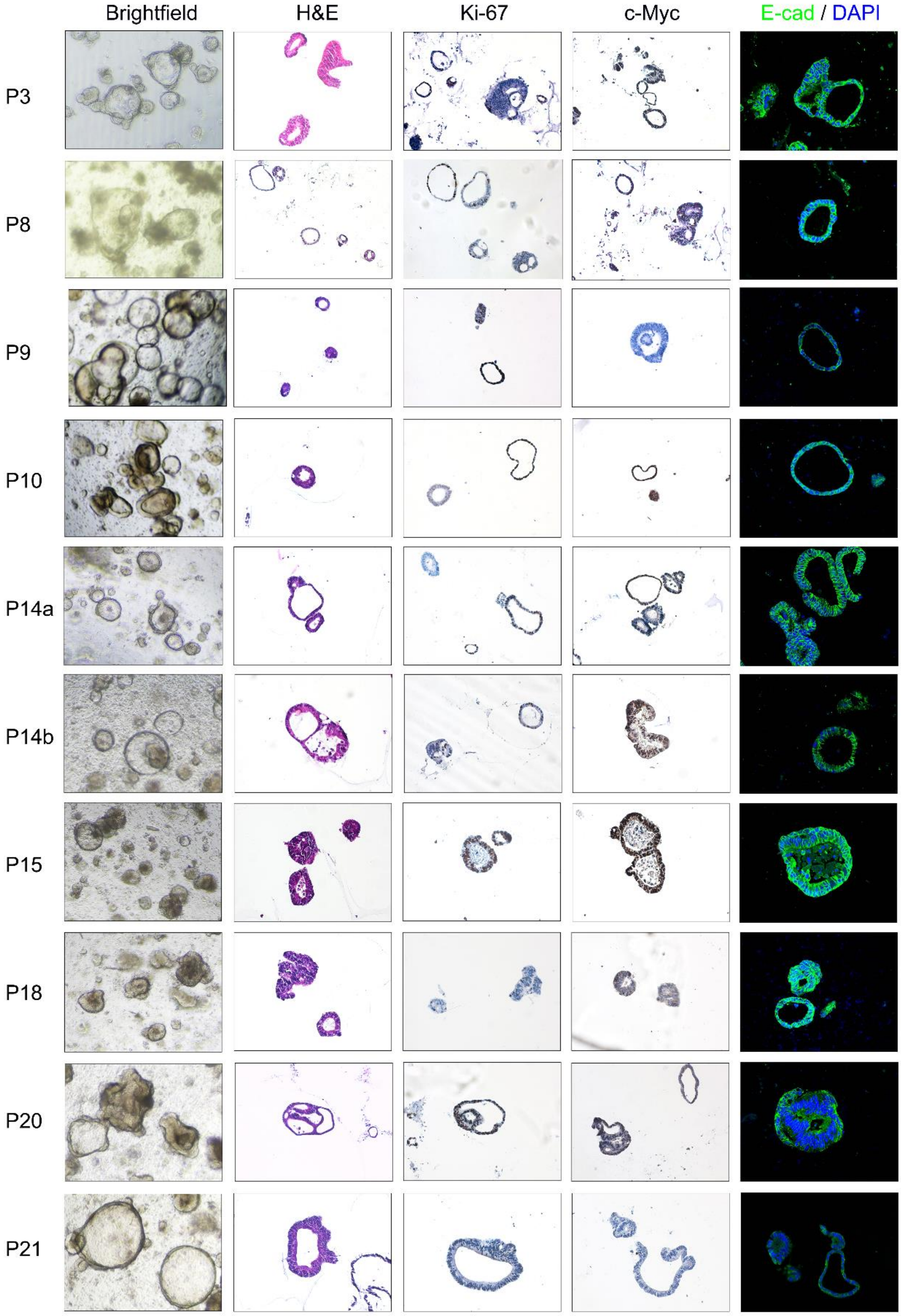
Brightfield
H&E
Ki-67
c-Myc
E-cad / DAPI
P3
P8
P9
P10
P14a
P14b
P15
P18
P20
P21

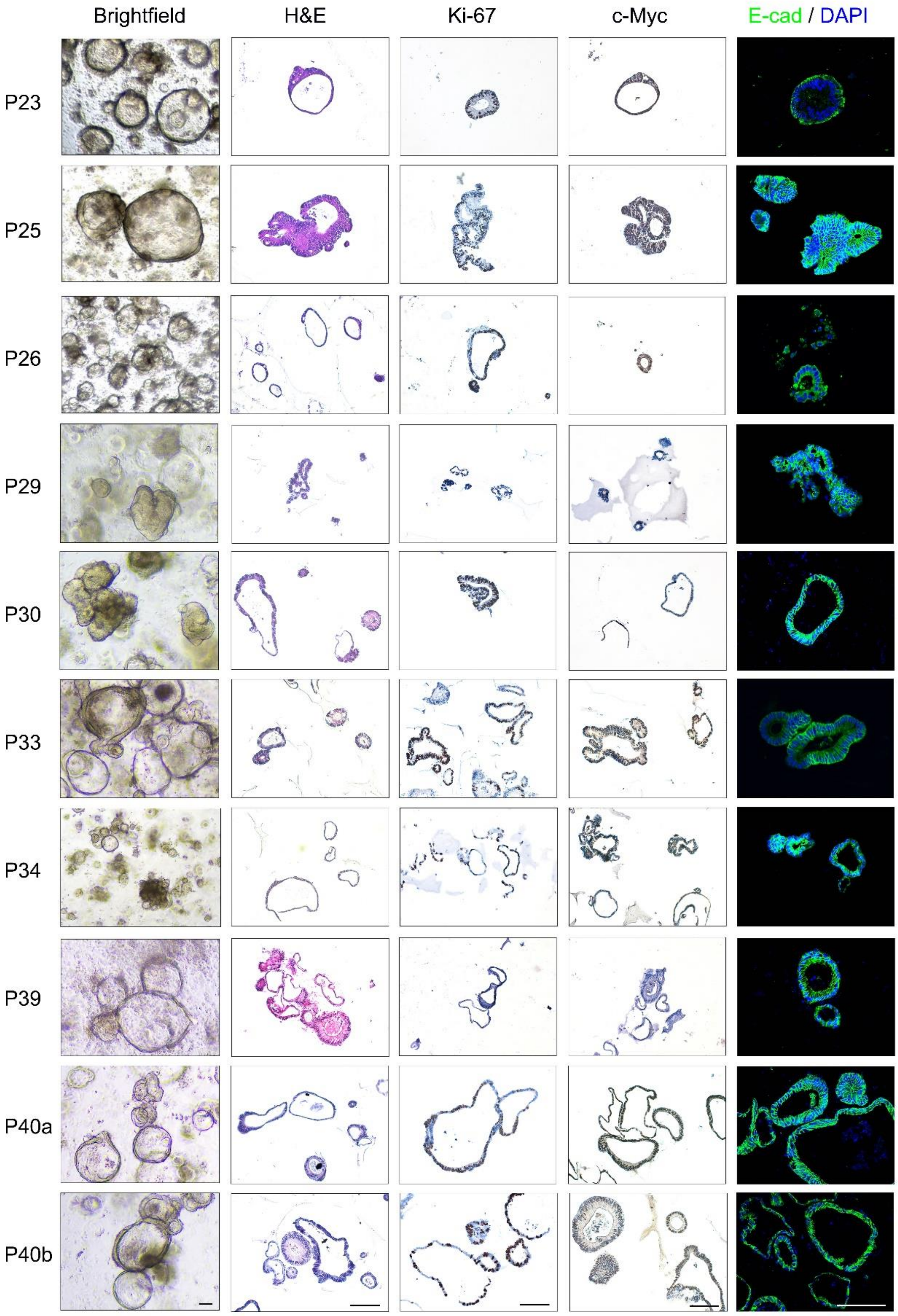

Figure S3. Characterization of HRCA-PDO biobank derived from different patients. Organoid arrays showed the bright-field, H&E, Ki-67, c-Myc, and E-cad staining in 20 HRCA-PDO lines. Black and white scale bars, 50 μm.

A

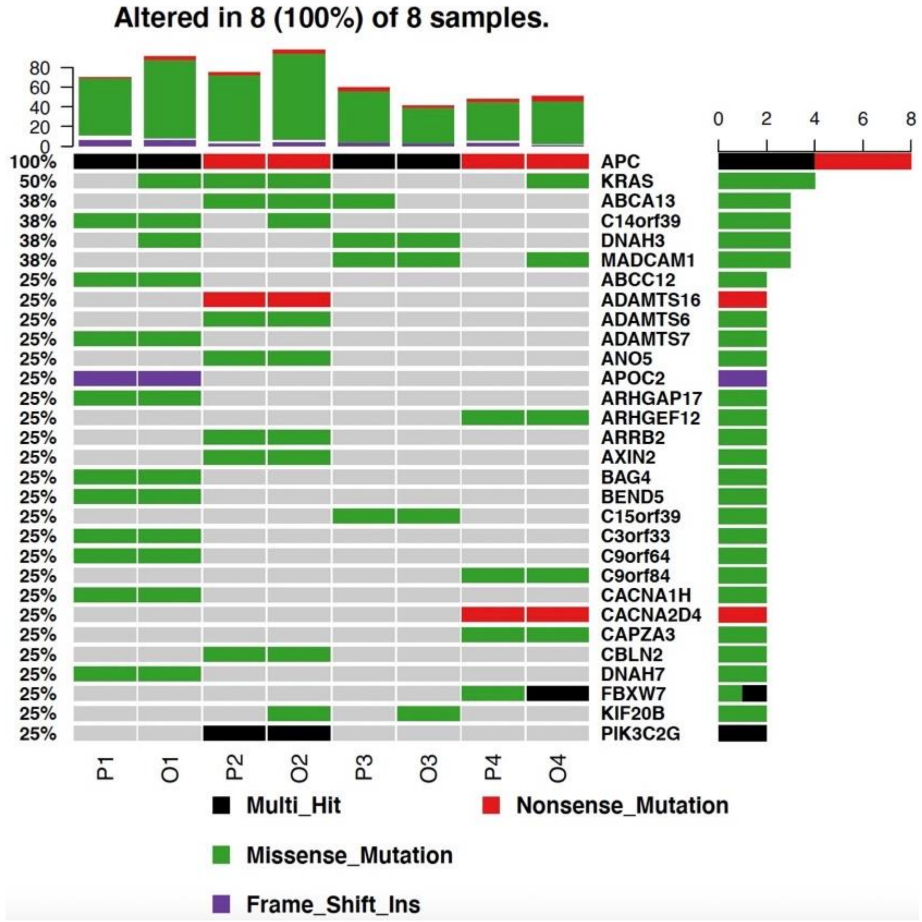

Altered in 8 (100%) of 8 samples.
APC
KRAS
ABCA13
C14orf39
DNAH3
MADCAM1
ABCC12
ADAMTS16
ADAMTS6
ADAMTS7
ANO5
APOC2
ARHGAP17
ARHGEF12
ARRB2
AXIN2
BAG4
BEND5
C15orf39
C3orf33
C9orf64
C9orf84
CACNA1H
CACNA2D4
CAPZA3
CBLN2
DNAH7
FBXW7
KIF20B
PIK3C2G
P1
O1
P2
O2
P3
O3
P4
O4
Multi_Hit
Nonsense_Mutation
Missense_Mutation
Frame_Shift_Ins

B

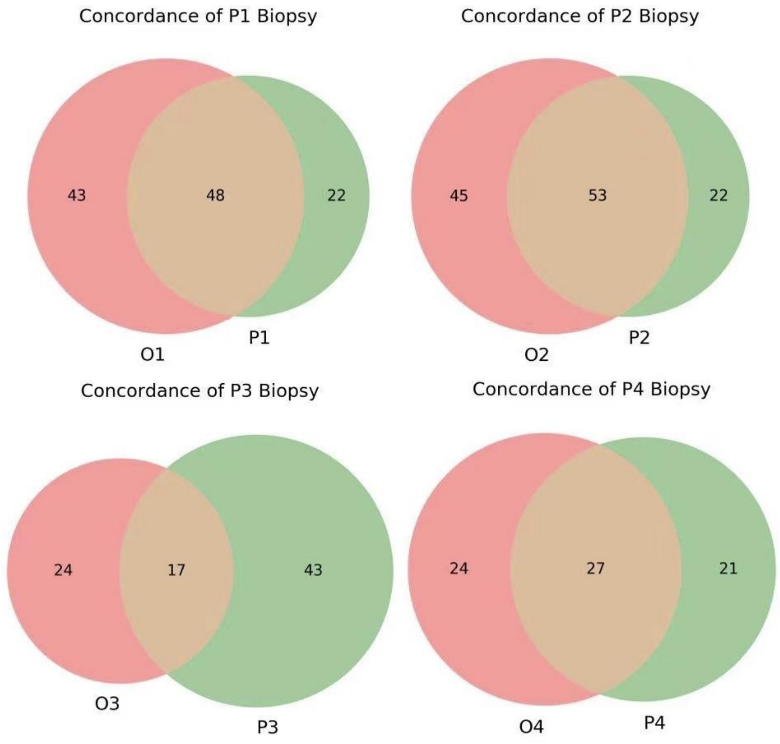

Concordance of P1 Biopsy
43
48
22
O1
P1
Concordance of P2 Biopsy
45
53
22
O2
P2
Concordance of P3 Biopsy
24
17
43
O3
P3
Concordance of P4 Biopsy
24
27
21
O4
P4

Figure S4. The mutation landscapes of adenomas from four patients were displayed comparing to the corresponding HRCA-PDOs. A. The types of genetic alteration (noted by color code) were displayed for the commonly mutated genes. B. The Venn diagrams indicated the number of organoid only, adenoma biopsy only and concordant mutated genes for P(O)1-4.

A

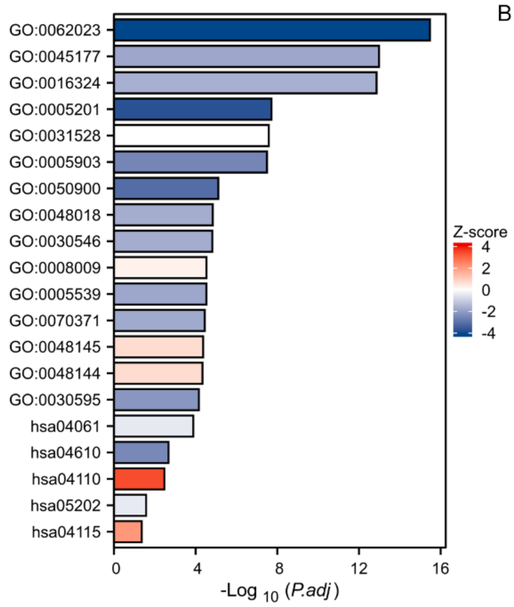

B

ONTOLOGY	ID	Description	p.adjust	zscore
BP	GO:0050900	leukocyte migration	7.74815E-06	-3.13049517
BP	GO:0048732	gland development	7.74815E-06	0.288675135
BP	GO:0048145	regulation of fibroblast proliferation	4.29438E-05	0.727606875
BP	GO:0048144	fibroblast proliferation	4.62584E-05	0.727606875
BP	GO:0030595	leukocyte chemotaxis	6.95385E-05	-2.19089023
CC	GO:0062023	collagen-containing extracellular matrix	3.34543E-16	-4.34121571
CC	GO:0045177	apical part of cell	1.04169E-13	-1.80739223
CC	GO:0016324	apical plasma membrane	1.37503E-13	-1.63299316
CC	GO:0031528	microvillus membrane	2.61649E-08	0
CC	GO:0005903	brush border	3.2015E-08	-2.5584086
MF	GO:0005201	extracellular matrix structural constituent	1.92747E-08	-3.77171134
MF	GO:0048018	receptor ligand activity	1.45434E-05	-1.69705627
MF	GO:0030546	signaling receptor activator activity	1.52452E-05	-1.69705627
MF	GO:0008009	chemokine activity	2.95301E-05	0.277350098
MF	GO:0005539	glycosaminoglycan binding	2.97288E-05	-1.82574186
KEGG	hsa04061	Viral protein interaction with cytokine and cytokine receptor	0.000130239	-0.4472136
KEGG	hsa04610	Complement and coagulation cascades	0.00213088	-2.5
KEGG	hsa04110	Cell cycle	0.00340318	3.441236008
KEGG	hsa05202	Transcriptional misregulation in cancer	0.0265519	-0.42640143
KEGG	hsa04115	p53 signaling pathway	0.043658599	2.110579412

C

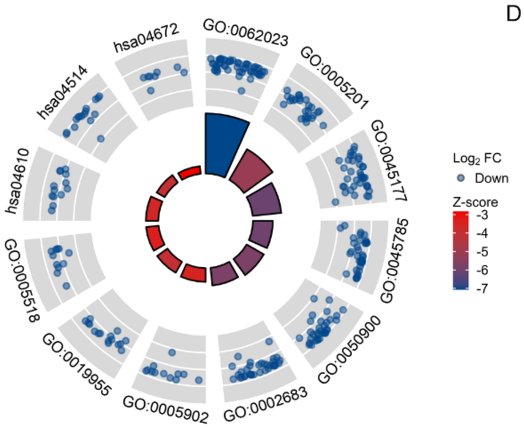

D

ONTOLOGY	ID	Description	p.adjust	zscore
BP	GO:0045785	positive regulation of cell adhesion	3.61492E-06	-6
BP	GO:0050900	leukocyte migration	3.61492E-06	-5.744562647
BP	GO:0002683	negative regulation of immune system process	1.30691E-05	-5.744562647
CC	GO:0062023	collagen-containing extracellular matrix	1.02016E-16	-7.071067812
CC	GO:0045177	apical part of cell	1.70207E-08	-6.08276253
CC	GO:0005902	microvillus	0.000138334	-3.464101615
MF	GO:0005201	extracellular matrix structural constituent	2.87789E-10	-5.099019514
MF	GO:0019955	cytokine binding	0.000273683	-4
MF	GO:0005518	collagen binding	0.0002875	-3.31662479
KEGG	hsa04610	Complement and coagulation cascades	0.000302301	-3.605551275
KEGG	hsa04514	Cell adhesion molecules	0.002133318	-4
KEGG	hsa04672	Intestinal immune network for IgA production	0.005601088	-2.828427125

E

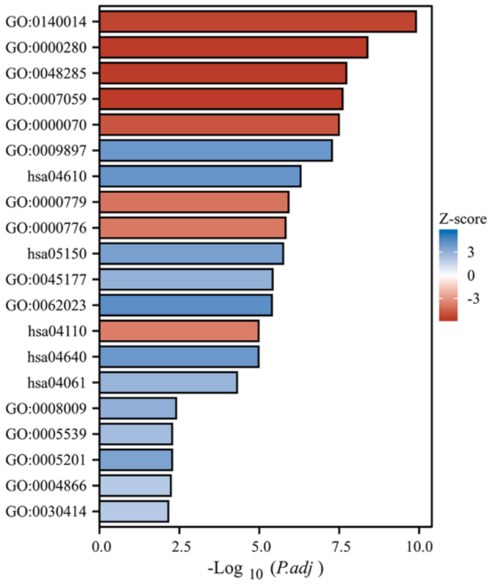

F

ONTOLOGY	ID	Description	p.adjust	zscore
BP	GO:0140014	mitotic nuclear division	1.25546E-10	-5.333333333
BP	GO:0000280	nuclear division	4.13882E-09	-5.863527299
BP	GO:0048285	organelle fission	1.89636E-08	-5.642447102
BP	GO:0007059	chromosome segregation	2.48869E-08	-5.916079783
BP	GO:0000070	mitotic sister chromatid segregation	3.22721E-08	-4.898979486
CC	GO:0009897	external side of plasma membrane	5.33971E-08	4.003203845
CC	GO:0000779	condensed chromosome, centromeric region	1.21504E-06	-4.024922359
CC	GO:0000776	kinetochore	1.51075E-06	-3.900067476
CC	GO:0045177	apical part of cell	3.83094E-06	2.959320151
CC	GO:0062023	collagen-containing extracellular matrix	4.04958E-06	4.351941399
MF	GO:0008009	chemokine activity	0.004002726	3
MF	GO:0005201	extracellular matrix structural constituent	0.005336834	3.5
MF	GO:0005539	glycosaminoglycan binding	0.005336834	2.523573073
MF	GO:0004866	endopeptidase inhibitor activity	0.005855025	2
MF	GO:0030414	peptidase inhibitor activity	0.007032072	2
KEGG	hsa04610	Complement and coagulation cascades	5.10618E-07	4.123105626
KEGG	hsa05150	Staphylococcus aureus infection	1.79296E-06	3.638034376
KEGG	hsa04640	Hematopoietic cell lineage	1.05422E-05	4
KEGG	hsa04110	Cell cycle	1.05422E-05	-3.771236166
KEGG	hsa04061	Viral protein interaction with cytokine and cytokine receptor	4.96893E-05	2.840187787

Figure S5. GO and KEGG analysis based on DEGs. (A-B) Function enriched analysis for DEGs between adenoma and mucosa. (C-D) GO and KEGG analysis based on downregulated DEGs between adenoma and mucosa. (D-E) Function enriched analysis for DEGs between adenoma and CA organoid.

A

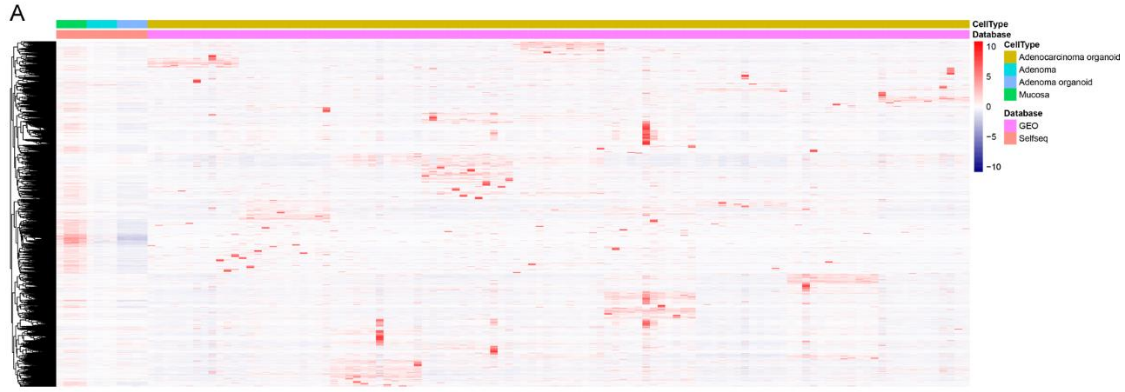

B

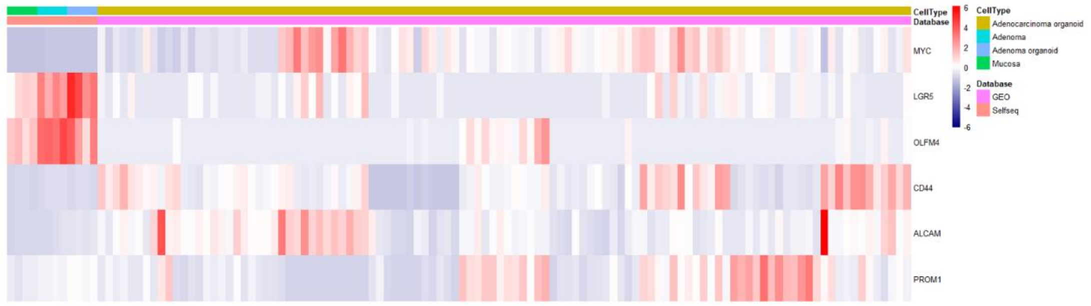

C

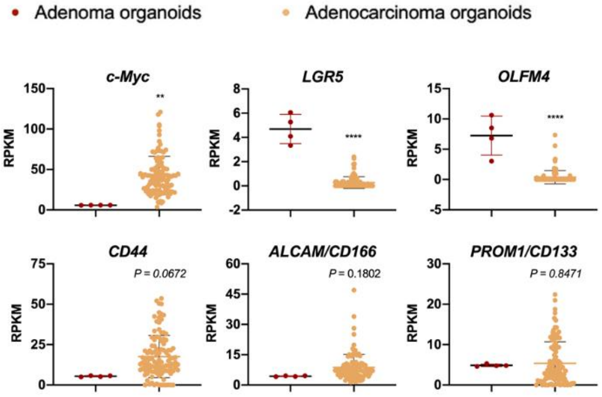

D

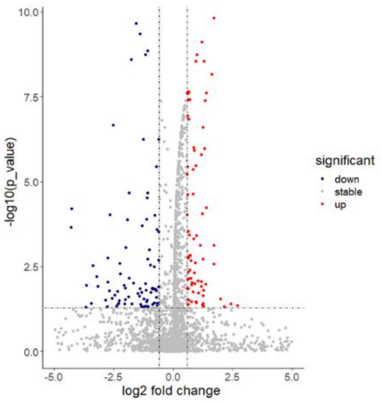

E

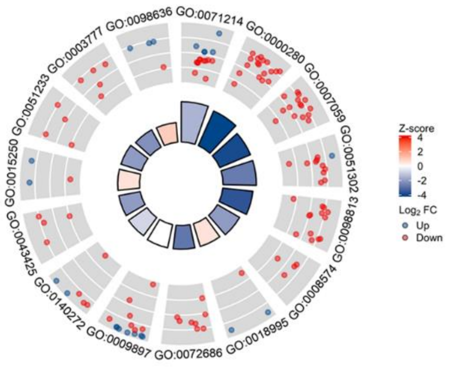

F

ONTOLOGY	ID	Description	p.adjust	zscore
BP	GO:0071214	cellular response to abiotic stimulus	0.000299292	-1.5
BP	GO:0000280	nuclear division	0.000523993	-4.24264069
BP	GO:0007059	chromosome segregation	0.001563114	-3.87298335
BP	GO:0051302	regulation of cell division	0.001563114	-2.7136021
BP	GO:0098813	nuclear chromosome segregation	0.003244695	-3.60555128
CC	GO:0018995	host cellular component	0.014931945	0.577350269
CC	GO:0072686	mitotic spindle	0.014931945	-2.82842712
CC	GO:0009897	external side of plasma membrane	0.014931945	0
CC	GO:0051233	spindle midzone	0.024239128	-2
CC	GO:0098636	protein complex involved in cell adhesion	0.040050635	1
MF	GO:0008574	plus-end-directed microtubule motor activity	0.009667418	-2
MF	GO:0140272	exogenous protein binding	0.020042056	-0.81649658
MF	GO:0043425	bHLH transcription factor binding	0.023301734	-2
MF	GO:0015250	water channel activity	0.023301734	0.577350269
MF	GO:0003777	microtubule motor activity	0.037708941	-2.23606798

Figure S6. Comparison of gene expression profiles between colorectal adenoma organoids and adenocarcinoma organoids. (A) Clustered heatmap of RPKMs of 2689 genes expressed in mucosa, adenoma, adenoma organoids (RNA-seq results as Figure 3A) and adenocarcinoma organoids (data from GSE65253) after normalization. (B) Clustered heatmap of RPKMs of *c-Myc*, *LGR5*, *OLFM4*, *CD44*, *CD166 (ALCAM)* and *CD133 (PROM1)* expressed in four groups. (C) The results of RPKMs of *c-Myc*, *LGR5*, *OLFM4*, *CD44*, *CD166 (ALCAM)* and *CD133 (PROM1)* in adenoma organoids and adenocarcinoma organoids were plotted for comparison. All data were presented as mean ± SD (n = 4 in adenoma organoid group, n=108 in adenocarcinoma organoid group) and subjected to Student's *t*-test. ** indicates P < 0. 01. **** indicates P < 0. 0001. (D) The volcano plot showed the 194 DEGs (117 downregulated and 77 upregulated, $P_{adj} < 0.05$) in adenocarcinoma organoids versus adenoma organoids. (E-F) GO analysis based on DEGs.

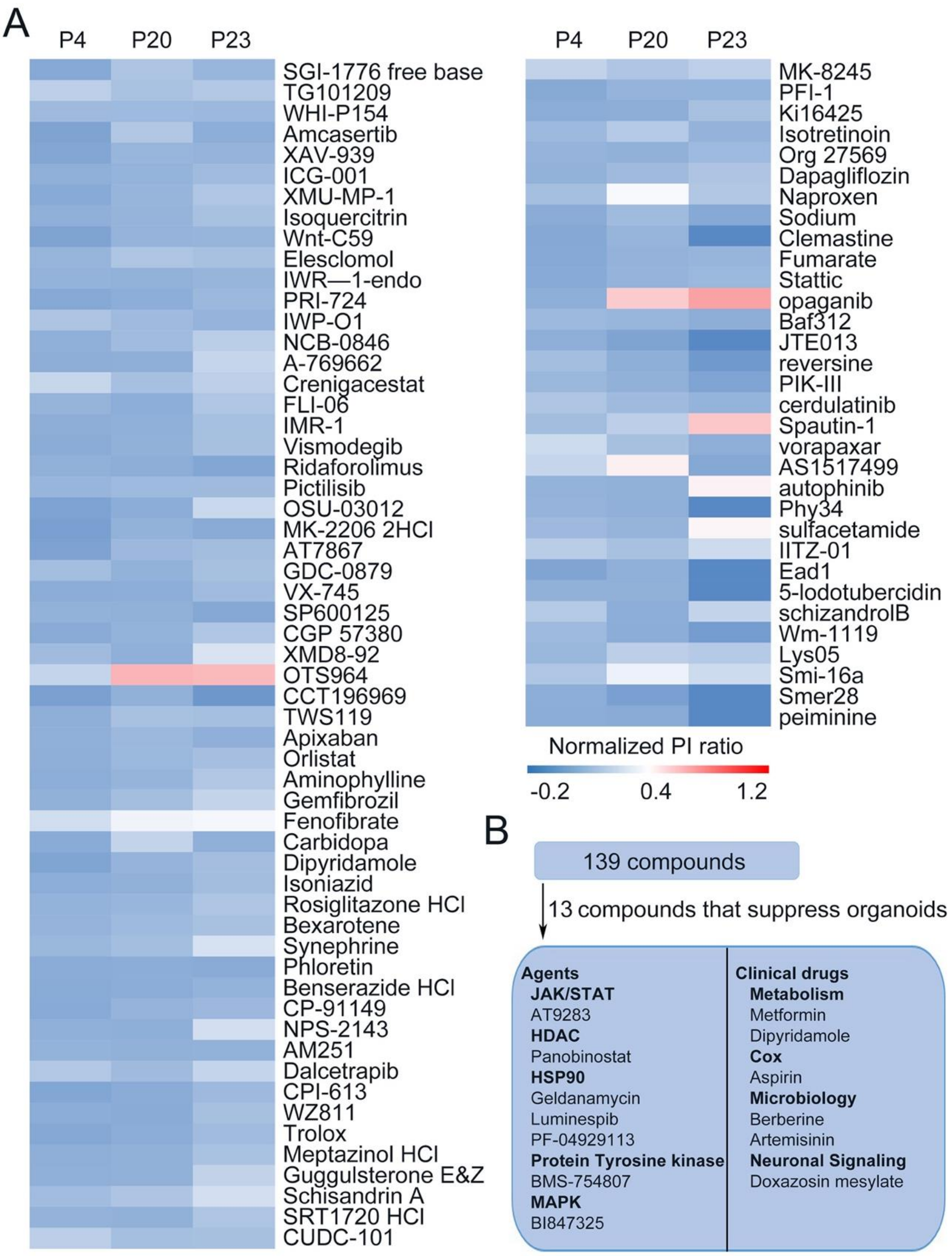

Figure S7. High-throughput screening of a compound library with the human HRCA-PDOs. (A) Heatmap showed normalized PI ratio (PI/Hoechst) in 3 CA organoid lines (P4, P20 and P23) after treatment of 108 compounds. Colors range from blue (low mortality) to

red (high mortality). P, patient. (B) Drug screening in CA organoids using a compound library. From a library of 139 compounds (108 compounds plus 31 compounds in Figure 3B), we successfully screened out 12 compounds that were able to significantly suppress organoid growth.

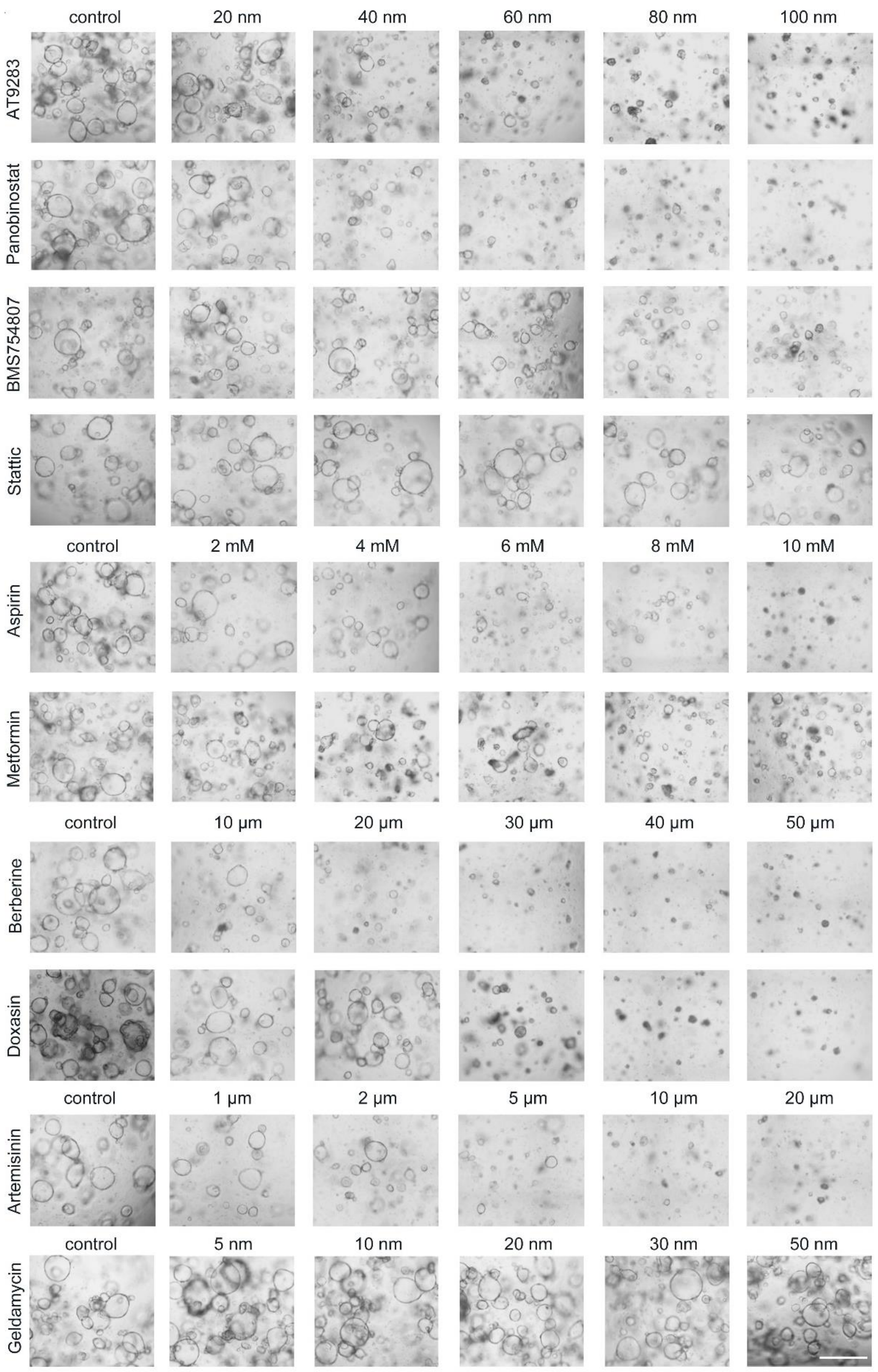
control
20 nm
40 nm
60 nm
80 nm
100 nm
AT9283
Panobinostat
BMS754807
Stattic
control
2 mM
4 mM
6 mM
8 mM
10 mM
Aspirin
Metformin
control
10 μm
20 μm
30 μm
40 μm
50 μm
Berberine
Doxasin
control
1 μm
2 μm
5 μm
10 μm
20 μm
Artemisinin
control
5 nm
10 nm
20 nm
30 nm
50 nm
Geldamycin

Figure S8. Compounds affect the morphology of HRCA organoids in a dose-dependent manner. Compared with Stattic and Geldamycin, those candidate hits including AT9283, Panobinostat, BMS754807, Aspirin, Metformin, Doxazosin, Berberine and Artemisinin inhibited the surface area and the transparency of organoids in a dose-dependent manner. Especially, AT9283 (40 nM), Panobinostat (40 nM), Metformin (6 mM) and Berberine (10 μM) significantly reduced the surface area and transmittance of organoids at indicated concentrations. White scale bar, 500 μm.

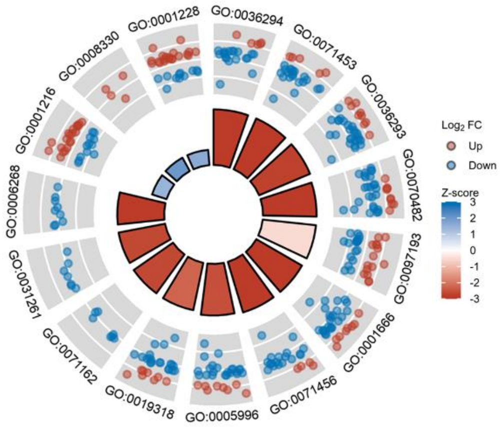

ONTOLOGY	ID	Description	p.adjust
BP	GO:0036294	cellular response to decreased oxygen levels	5.17E-07
BP	GO:0071453	cellular response to oxygen levels	5.17E-07
BP	GO:0036293	response to decreased oxygen levels	5.41E-07
BP	GO:0070482	response to oxygen levels	9.01E-07
BP	GO:0097193	intrinsic apoptotic signaling pathway	9.05E-07
BP	GO:0001666	response to hypoxia	1.18E-06
BP	GO:0071456	cellular response to hypoxia	1.18E-06
BP	GO:0005996	monosaccharide metabolic process	1.73E-06
BP	GO:0019318	hexose metabolic process	4.48E-06
BP	GO:0006268	DNA unwinding involved in DNA replication	6.83E-06
CC	GO:0071162	CMG complex	5.11E-06
CC	GO:0031261	DNA replication preinitiation complex	5.96E-06
MF	GO:0001216	DNA-binding transcription activator activity	0.098790377
MF	GO:0008330	protein tyrosine/threonine phosphatase activity	0.098790377
MF	GO:0001228	DNA-binding transcription activator activity, RNA polymerase II-specific	0.098790377

Figure S9. Gene Ontology analysis for the DEGs between metformin-treated and control HRCA-PDOs.

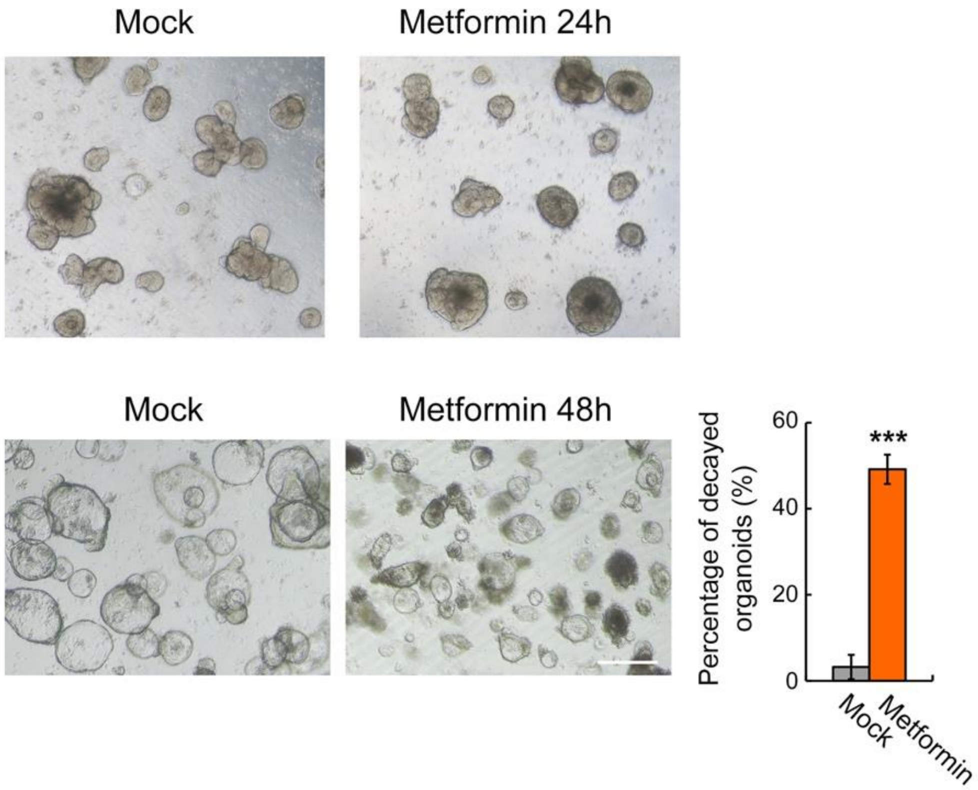

Figure S10. Metformin treatment led to a substantial decay of HRCA-PDOs after 48 hours. After metformin (5 mM) treatment for 24 hours, the bright-field images showed no obvious change on the morphology of HRCA-PDOs (upper panel), but indicated that metformin led to a substantial decay of HRCA-PDOs after 48 h treatment (lower panel). Scale bar, 100 μm. All data were presented as mean±SD (n = 6) and subjected to Student's *t*-test. *** indicates $P < 0.001$.

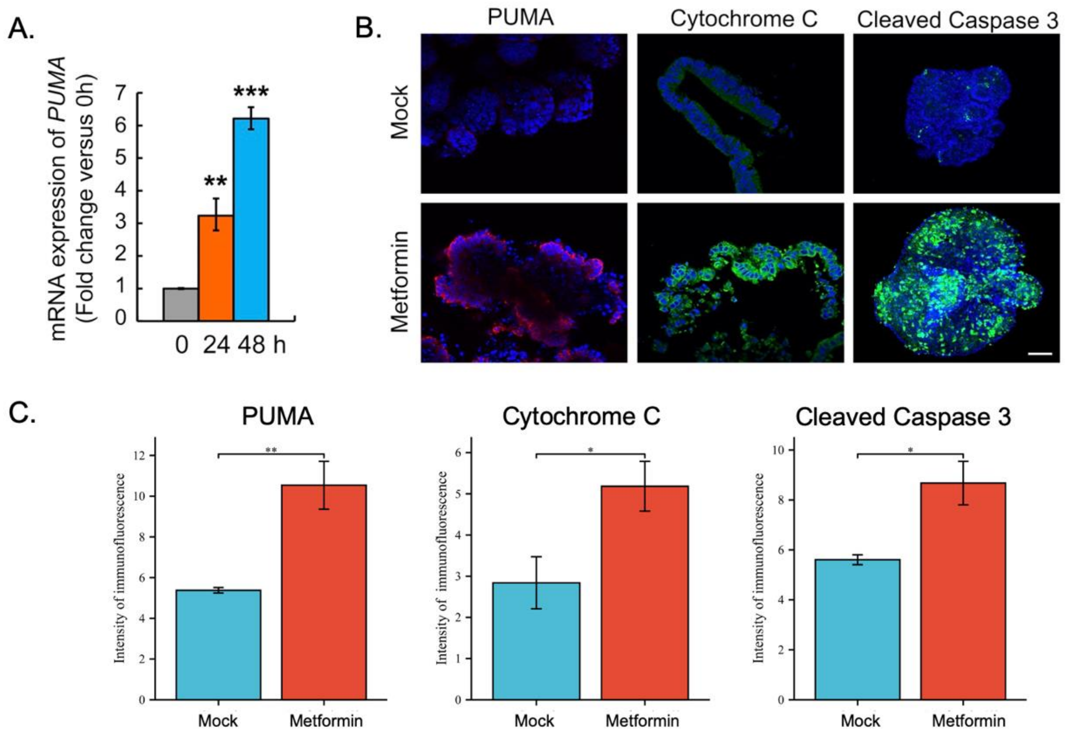

Figure S11. Metformin treatment promoted apoptosis in HRCA-PDOs. After the metformin treatment for 48 h, HRCA-PDOs were harvested to examine the expression of *PUMA* by qRT-PCR (A), as well as stained for PUMA (red), cytochrome-C (green), cleaved caspase-3 (green) and DAPI (blue), then subject to confocal cross-sectioning (B). The intensity of immunostaining was quantified by ImageJ software. A representative result of three independent experiments was shown. Scale bar, 50 μm. All data were presented as mean±S.D (n = 4-6, A; n=12, C) and subjected to One-way ANOVA followed by the Bonferroni *post hoc* test in A, and subjected to Student *t*-test in C. * indicates $P < 0.05$, ** indicates $P < 0.01$ and *** indicates $P < 0.001$ versus mock group.

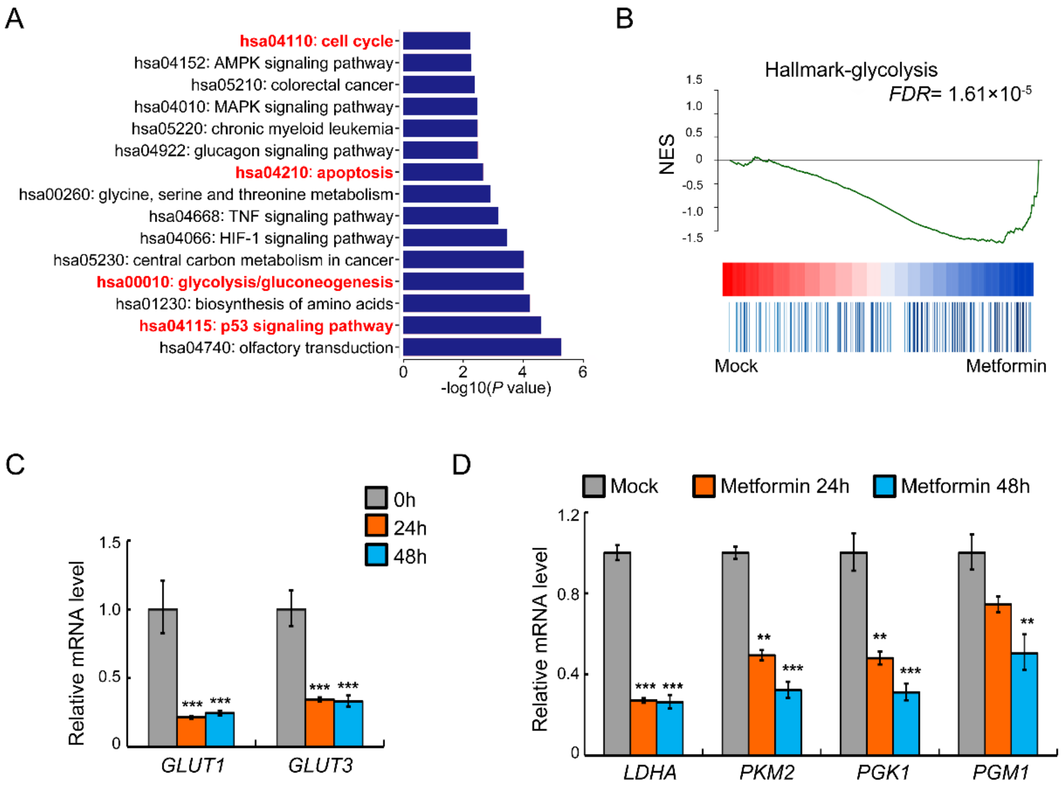

Figure S12. Metformin regulated the glycolysis of colorectal adenoma. (A) GSEA indicated significant enrichment of DEGs involved in “p53 signaling pathway”, “apoptosis” and “cell cycle” (red). (B) GSEA illustrated intensively down-regulated genes involved in glycolysis among metformin treatment group. (FDR=1.61×10-5). (C) CA organoids were harvested to examine the expression of *GLUT1/3* using qRT-PCR after metformin (5 mM, 24 h, 48 h) treatment. (D) CA organoids were harvested to examine the expression of *LDHA*, *PKM2*, *PGK1*, *PGM1* using qRT-PCR. All data were presented as mean ± SD (n = 4-5) and subjected to One-way ANOVA by the Bonferroni post hoc test *** indicates p<0.001. ** indicates $p < 0.01$. * indicates $p < 0.05$.

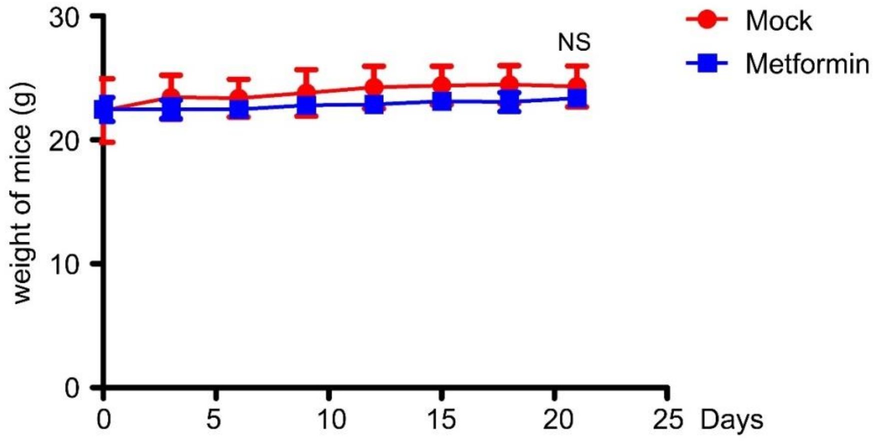

Figure S13. Body weight changes in mice during metformin intervention. The weight of mice in the control (PBS, n = 5) and treatment (metformin, n = 4) group was measured every 3 days for 3 weeks. All data were presented as mean±S.D and Student-t test was performed at each time point. NS indicates no significant difference.

Supplemental Tables

Table S1. The biobank of Patients-derived HRCA organoids (HRCA-PDOs).

Case	Sex	Age	Size(mm)	Location	Pathology	Single/Multiple lesions	Risk	Organoid culture
P1	female	72	10*15	sigmoid colon	tubular adenoma	multiple	high	yes
P2	female	53	10*15	sigmoid colon	tubular adenoma	multiple	high	yes
P3	man	57	10*10	sigmoid colon	tubular adenoma	single	high	no
P4	female	66	12*10	sigmoid colon	tubular adenoma	single	high	yes
P5	female	73	15*15	sigmoid colon	villous adenoma	single	high	yes
P6	man	61	20*15	descending colon	Villous tubular adenoma	multiple	high	yes
P7	man	72	10*8	rectum	tubular adenoma	single	low	yes
P8	man	70	10*8	rectum	tubular adenoma	single	low	no
P9	female	65	12*8	sigmoid colon	tubular adenoma	multiple	high	yes
P10	man	68	10*8	sigmoid colon	tubular adenoma	multiple	high	yes
P11	female	67	10*8	transverse colon	tubular adenoma	multiple	high	yes
P12	man	67	10*15	ascending colon	tubular adenoma	multiple	high	yes
P13	man	69	18*10	sigmoid colon	Villous tubular adenoma with low-grade neoplasia	multiple	high	no
P14	man	79	20*25	sigmoid colon	Villous tubular adenoma with high-grade neoplasia (P14a)	multiple	high	yes
			10*12	transverse colon	tubular adenoma (P14b)			yes
			8*10	ascending colon	tubular adenoma (P14c)			yes
P15	man	44	15*15	descending colon	Villous tubular adenoma	single	high	yes
P16	man	55	30*30	descending colon	Villous tubular adenoma with high-grade neoplasia	single	high	yes
P17	female	68	8*6	ascending colon	tubular adenoma	single	low	yes
P18	female	62	10*10	sigmoid colon	tubular adenoma	multiple	high	yes
P19	female	62	8*8	hepatic flexure	tubular adenoma	multiple	high	yes
P20	man	52	6*8	sigmoid colon	Villous tubular adenoma	multiple	high	yes
P21	man	62	8*10	transverse colon	tubular adenoma	multiple	high	yes
P22	female	71	18*15	rectum	tubular adenoma	multiple	high	no
P23	man	58	8*10	hepatic flexure	Serrated adenoma	single	high	yes
P24	female	64	12*15	hepatic flexure	tubular adenoma	multiple	high	yes
P25	female	36	10*8	sigmoid colon	tubular adenoma	single	low	yes
P26	female	70	15*15	ascending colon	tubular adenoma	multiple	high	yes
P27	man	47	10*15	sigmoid colon	tubular adenoma	multiple	high	no
P28	man	66	15*20	sigmoid colon	tubular adenoma	multiple	high	yes
			10*8	transverse colon	tubular adenoma			no
P29	man	53	12*15	sigmoid colon	tubular adenoma	single	high	yes
P30	female	77	10*12	sigmoid colon	tubular adenoma	single	high	yes
P31	female	78	12*15	sigmoid colon	Sessile serrated adenoma	multiple	high	yes
P32	man	66	12*15	sigmoid colon	tubular adenoma (P32a)	multiple	high	yes
			12*15	ascending colon	tubular adenoma (P32b)			yes
P33	man	58	20*25	descending colon	tubular adenoma (P33a)	multiple	high	yes
			20*25	sigmoid colon	tubular adenoma (P33b)			yes
P34	man	63	10*10	cecum	tubular adenoma	single	high	no
P35	female	68	12*10	transverse colon	tubular adenoma	single	high	yes

P36	female	63	12*15	sigmoid colon	tubular adenoma	single	high	yes
P37	man	62	12*15	rectum	tubular adenoma	single	high	yes
P38	female	59	20*20	hepatic flexure	tubular adenoma	single	high	no
P39	man	58	8*10	descending colon	tubular adenoma	multiple	high	yes
			15*18	sigmoid colon	tubular adenoma			no
P40	man	61	10*10	sigmoid colon	tubular adenoma (P40a)	multiple	high	yes
			10*10	sigmoid colon	tubular adenoma (P40b)			yes

Table S2. Primers for RT-qPCR.

	Forward (5'-3')	Reverse (5'-3')
h-CD44	CTGCCGCTTTGCAGGTGTA	CATTGTGGGCAAGGTGCTATT
h-c-Myc	GTCAAGAGGCGAACACACAAC	TTGGACGGACAGGATGTATGC
h-CD166	ACTTGACGTACCTCAGAATCTCA	CATCGTCGTACTGCACACTTT
h-CD44	CTGCCGCTTTGCAGGTGTA	CATTGTGGGCAAGGTGCTATT
h-CD133	AGTCGGAAACTGGCAGATAGC	GGTAGTGTTGTACTGGGCCAAT
h-LGR5	GAGTTACGTCTTGCGGGAAAC	TGGGTACGTGTCTTAGCTGATTA
h-OLFM4	ACTGTCCGAATTGACATCATGG	TTCTGAGCTTCCACCAAAACTC
h-PUMA	GACCTCAACGCACAGTACGAG	AGGAGTCCCATGATGAGATTGT
h-PGK1	GAACAAGGTTAAAGCCGAGCC	GTGGCAGATTGACTCCTACCA
h-PGM1	CCAAACCGACTGAAGATCCGT	CATGTTTCGATCCCCATCTCC
h-GLUT1	TCTGGCATCAACGCTGTCTTC	CGATACCGGAGCCAATGGT
h-GLUT3	GTGACCTTGCAACTTCATGTC	AAATGGGACCCTGCCTTACTG
h-PKM2	ATGTCGAAGCCCCATAGTGAA	TGGGTGGTGAATCAATGTCCA
h-LDHA	TTGACCTACGTGGCTTGGAAG	GGTAACGGAATCGGGCTGAAT
h-OCT1	ACGGTGGCGATCATGTACC	CCCATTCTTTTGAGCGATGTGG
h-OCT2	CATCGTCACCGAGTTTAACCTG	AGCCGATACTCATAGAGCCAAT
h-SLC22A3	CTTCCGCTTCCTGCAAGGT	AAGTAGGCAATTCCAGGGAGA
h-SLC29A4	GCTTTCACGGATACTACATTGGA	ATGTCAAACACGATGGAGGTC
h-NOX1	TTGTTTGGTTAGGGCTGAATGT	GCCAATGTTGACCCAAGGATTTT
h-Mate1	TTCATAAGCTCCGTGTTCTGTG	AGTGACATTGATAACCGCGATT
h-Mate2	CAGAGGCTTTGGGACTGAGAT	GTCACATGCCGAAGACAAACC
m-C-Myc	TAGTGCTGCATGAGGAGACA	CATCAATTTCTTCCTCATCTTC
m-Nox1	GGTTGGGGCTGAACATTTTTC	TCGACACACAGGAATCAGGAT
m-Lgr5	CCTACTCGAAGACTTACCCAGT	GCATTGGGGTGAATGATAGCA
m-Olfm4	CAGCCACTTTCCAATTTCACTG	GCTGGACATACTCCTTCACCTTA

h, human; m, mouse.

Table S3. Concentration of drugs in compound library.

Product Name	Concentration
S3I-201	100 nM
AT9283	100 nM
SGI-1776 free base	100 nM
TG101209	100 nM
WHI-P154 (JAK/STAT)	100 nM
Amcasertib (BBI503)	100 nM
XAV-939	100 nM
ICG-001	100 nM
XMU-MP-1	100 nM
Isoquercitrin	100 nM
Wnt-C59 (C59)	100 nM
Elesclomol (STA-4783)	100 nM
IWR—1-endo	100 nM
PRI-724	100 nM
IWP-O1	100 nM
NCB-0846	100 nM
A-769662	100 nM
Crenigacestat (LY3039478)	100 nM
FLI-06	100 nM
IMR-1	100 nM
Vismodegib (GDC-0449)	100 nM
5-Bromoindole	100 nM
A-674563	100 nM
Geldanamycin	100 nM
Ridaforolimus (Deforolimus, MK-8669)	100 nM
Pictilisib (GDC-09410)	100 nM
OSU-03012 (AR-12)	100 nM
MK-2206 2HCl	100 nM
AT7867	100 nM
GSK2334470	100 nM
Luminespib (AUY-922, NVP-AUY922)	100 nM
ETP-46464 (PI3K)	100 nM
PD0325901	100 nM
BX-795	100 nM
PF-04929113 (SNX-5422)	100 nM
GDC-0879	100 nM
VX-745	100 nM
SP600125	100 nM
CGP 57380	100 nM
XMD8-92	100 nM
OTS964	100 nM

CCT196969	100 nM
BI-847325	100 nM
CEP-32496	100 nM
TWS119	100 nM
MK-8353 (SCH900353)	100 nM
Apixaban	100 nM
Orlistat	100 nM
Aminophylline	100 nM
Gemfibrozil	100 nM
Fenofibrate	100 nM
Carbidopa	100 nM
Dipyridamole	100 nM
Isoniazid	100 nM
Rosiglitazone HCl	100 nM
Bexarotene	100 nM
Synephrine	100 nM
Phloretin	100 nM
Benserazide HCl	100 nM
CP-91149	100 nM
NPS-2143	100 nM
AM251	100 nM
Dalcetrapib (JTT-705, RO4607381)	100 nM
CPI-613	100 nM
WZ811	100 nM
Trolox	100 nM
Meptazinol HCl	100 nM
Guggulsterone E&Z	100 nM
Schisandrin A	100 nM
Panobinostat (LBH589)	100 nM
SU11274	100 nM
SRT1720 HCl	100 nM
CUDC-101	100 nM
BMS-754807	100 nM
MK-8245	100 nM
PFI-1(PF-6405761)	100 nM
Ki16425	100 nM
Isotretinoin	100 nM
Org 27569	100 nM
Dapagliflozin	100 nM
Naproxen Sodium	100 nM
Clemastine Fumarate	100 nM
Stattic	100 nM
opaganib	100 nM
Baf312	100 nM

JTE013	100 nM
reversine	100 nM
PIK-III	100 nM
cerdulatinib	100 nM
Spautin-1	100 nM
vorapaxar	100 nM
AS1517499	100 nM
autophinib	100 nM
Phy34	100 nM
sulfacetamide	100 nM
IITZ-01	100 nM
Ead1	100 nM
5-Iodotubercidin	100 nM
schizandrolB	100 nM
Wm-1119	100 nM
Lys05	100 nM
Smi-16a	100 nM
Smer28	100 nM
peiminine	100 nM
PF-04965842	100 nM
OroxylinA	100 nM
PFK158	100 nM
Oclacitinib	100 nM
artemisinin	50 μM
metformin	10 mM
Celecoxib	50 μM
doxazosin	50 μM
resveratrol	50 μM
glimepiride	50 μM
cimitidine	50 μM
dipyridamole	50 μM
isoniazid	50 μM
probucol	50 μM
rosiglitazone	50 μM
acemetacin	50 μM
Vc	50 μM
disulfiram	20 μM
mesalazine	20 μM
berberine	50 μM
aspirin	10 mM
curcurine	20 μM
Terazosin	100 nM
Compoud401	100 nM
Indole-3-carbinol	100 nM

Magnolol	100 nM
evodiamine	100 nM
Ml130	100 nM
Imd0354	100 nM
pdca	100 nM
curcumenol	100 nM
diethylmaleate	100 nM
Qnz	100 nM
Gsk583	100 nM
engeletin	100 nM
