# Supplementary material for: Establishment of a large-scale patient-derived high-risk colorectal adenoma organoid biobank for high-throughput and high-content drug screening
Source: BMC Med. 2023 Sep 4;21:336. doi: 10.1186/s12916-023-03034-y (PMC10478412; doi:10.1186/s12916-023-03034-y)
Supplement: Supplementary file 2 — Additional file 2. Detailed list of somatic mutations in primary high-risk colorectal adenomas and corresponding organoids. [file 12916_2023_3034_MOESM2_ESM.pdf]

Hugo_Symbol	Tumor_Sample_Barcod	Chromosome	Start_Position	End_Position	Variant_Classification	Variant_Type	Reference_Allele	Tumor_Seq_Allele1	Tumor_Seq_Allele2	Protein_Change	i_TumorVAF_WU	i_transcript_name
KRAS	O1	12	25380276	25380276	Missense_Mutation	SNP	T	T	C	KRAS:NM_033360:exon3:c.182A>G:p.Q61R	21.82	NM_033360
C14orf37	O1	14	58471800	58471800	Missense_Mutation	SNP	C	C	T	C14orf37:NM_001001872:exon7:c.2222G>A:p.R741Q	19.05	NM_001001872
DNAH3	O1	16	20996749	20996749	Missense_Mutation	SNP	C	C	T	DNAH3:NM_017539:exon48:c.7315G>A:p.G2439R	24.73	NM_017539
ABCC12	O1	16	48149472	48149472	Missense_Mutation	SNP	G	G	A	ABCC12:NM_033226:exon13:c.1843C>T:p.R615C	10.27	NM_033226
GJC1	O1	17	42882687	42882687	Missense_Mutation	SNP	G	G	A	GJC1:NM_005497:exon3:c.499C>T:p.R167W	13.78	NM_005497
TP53	O1	17	7578203	7578203	Missense_Mutation	SNP	C	C	T	TP53:NM_000546:exon6:c.646G>A:p.V216M	10.75	NM_000546
SMAD4	O1	18	48593466	48593466	Missense_Mutation	SNP	C	C	T	SMAD4:NM_005359:exon10:c.1217C>T:p.A406V	12.31	NM_005359
HAUS5	O1	19	36109580	36109580	Missense_Mutation	SNP	G	G	A	HAUS5:NM_015302:exon12:c.995G>A:p.R332H	18.03	NM_015302
NLRP5	O1	19	56565093	56565093	Missense_Mutation	SNP	C	C	T	NLRP5:NM_153447:exon13:c.3218C>T:p.T1073M	9.65	NM_153447
PCBP1	O1	2	70315174	70315174	Missense_Mutation	SNP	T	T	A	PCBP1:NM_006196:exon1:c.299T>A:p.L100Q	25	NM_006196
PRR23C	O1	3	138762733	138762733	Missense_Mutation	SNP	G	G	A	PRR23C:NM_001134657:exon1:c.730C>T:p.R244C	12.16	NM_001134657
APC	O1	5	112175273	112175273	Nonsense_Mutation	SNP	C	C	T	APC:NM_000038:exon16:c.3982C>T:p.Q1328*	43.3	NM_000038
CFB	O1	6	31915244	31915244	Missense_Mutation	SNP	C	C	T	CFB:NM_001710:exon4:c.604C>T:p.R202W	47.42	NM_001710
CLASRP	O1	19	45567607	45567609	In_Frame_Del	DEL	CTC	CTC	-	CLASRP:NM_007056:exon13:c.1149_1151delCTC:p.S384del	12.5	NM_007056
ITIH2	O1	10	7773844	7773844	Missense_Mutation	SNP	C	C	T	ITIH2:NM_002216:exon13:c.1532C>T:p.T511M	12.68	NM_002216
MICAL2	O1	11	12281344	12281344	Missense_Mutation	SNP	G	G	T	MICAL2:NM_014632:exon26:c.3234G>T:p.E1078D	35.71	NM_014632
FAM118B	O1	11	126110795	126110795	Nonsense_Mutation	SNP	G	G	A	FAM118B:NM_024556:exon4:c.195G>A:p.W65*	22.47	NM_024556
PLEKHA7	O1	11	16848121	16848121	Nonsense_Mutation	SNP	C	C	A	PLEKHA7:NM_001329630:exon10:c.889G>T:p.E297*	24.72	NM_001329630
IGSF22	O1	11	18730952	18730952	Missense_Mutation	SNP	G	G	A	IGSF22:NM_173588:exon18:c.2980C>T:p.R994C	27.78	NM_173588
CCDC179	O1	11	22881002	22881002	Missense_Mutation	SNP	C	C	T	CCDC179:NM_001195637:exon2:c.86G>A:p.R29Q	35	NM_001195637
ANO3	O1	11	26547183	26547183	Frame_Shift_Ins	DEL	A	A	-	ANO3:NM_001313726:exon8:c.882delA:p.K294fs	36.96	NM_001313726
LRRC4C	O1	11	40137645	40137646	Frame_Shift_Ins	INS	-	-	T	LRRC4C:NM_020929:exon5:c.197dupA:p.N66fs	32.95	NM_020929
ANP32E	O1	1	150199042	150199042	Missense_Mutation	SNP	C	C	A	ANP32E:NM_030920:exon5:c.579G>T:p.E193D	17.78	NM_030920
MS4A8	O1	11	60482518	60482518	Missense_Mutation	SNP	G	G	A	MS4A8:NM_031457:exon6:c.559G>A:p.V187M	14.81	NM_031457
PACS1	O1	11	66001356	66001356	Missense_Mutation	SNP	G	G	T	PACS1:NM_018026:exon16:c.1939G>T:p.A647S	34.15	NM_018026
INPPL1	O1	11	71944155	71944155	Missense_Mutation	SNP	A	A	G	INPPL1:NM_001567:exon17:c.1988A>G:p.Y663C	24.05	NM_001567
KLRC4	O1	12	10560957	10560957	Missense_Mutation	SNP	T	T	C	KLRC4:NM_013431:exon3:c.311A>G:p.N104S	44	NM_013431
CCDC91	O1	12	28605426	28605426	Missense_Mutation	SNP	G	G	A	CCDC91:NM_018318:exon10:c.940G>A:p.V314M	53.13	NM_018318
RYR2	O1	1	237774113	237774113	Missense_Mutation	SNP	G	G	A	RYR2:NM_001035:exon36:c.4735G>A:p.V1579M	29.29	NM_001035
KRT1	O1	12	53069243	53069243	Missense_Mutation	SNP	T	T	C	KRT1:NM_006121:exon9:c.1669A>G:p.S557G	20.18	NM_006121
PTPRQ	O1	12	80838175	80838175	Missense_Mutation	SNP	C	C	T	PTPRQ:NM_001145026:exon1:c.50C>T:p.T17I	28.81	NM_001145026
ZMYM6	O1	1	35453270	35453273	Frame_Shift_Ins	DEL	AAGA	AAGA	-	ZMYM6:NM_007167:exon16:c.3410_3413delTCTT:p.F1137fs	12.63	NM_007167
PCDH17	O1	13	58208888	58208888	Missense_Mutation	SNP	G	G	T	PCDH17:NM_001040429:exon1:c.2208G>T:p.E736D	17.82	NM_001040429
TMCO2	O1	1	40713748	40713749	Frame_Shift_Ins	INS	-	-	T	TMCO2:NM_001008740:exon1:c.89dupT:p.L30fs	23.81	NM_001008740
OR4N2	O1	14	20296298	20296298	Missense_Mutation	SNP	G	G	A	OR4N2:NM_001004723:exon2:c.691G>A:p.E231K	41.18	NM_001004723
C14orf39	O1	14	60903757	60903757	Missense_Mutation	SNP	G	G	A	C14orf39:NM_174978:exon18:c.1570C>T:p.L524F	45.28	NM_174978
C14orf39	O1	14	60932752	60932752	Missense_Mutation	SNP	G	G	A	C14orf39:NM_174978:exon11:c.917C>T:p.A306V	51.61	NM_174978
NEK9	O1	14	75563914	75563914	Missense_Mutation	SNP	C	C	T	NEK9:NM_001329237:exon17:c.2098G>A:p.E700K	36.21	NM_001329237
SNW1	O1	14	78189588	78189588	Missense_Mutation	SNP	G	G	A	SNW1:NM_001318844:exon11:c.1066C>T:p.R356W	11.11	NM_001318844
BEND5	O1	1	49224895	49224895	Missense_Mutation	SNP	C	C	T	BEND5:NM_024603:exon3:c.422G>A:p.R141Q	10.66	NM_024603
ADAMTS7	O1	15	79058622	79058622	Missense_Mutation	SNP	G	G	A	ADAMTS7:NM_014272:exon19:c.3631C>T:p.R1211W	10.04	NM_014272
KIF7	O1	15	90173714	90173714	Missense_Mutation	SNP	G	G	A	KIF7:NM_198525:exon16:c.3122C>T:p.T1041M	41.27	NM_198525
CACNA1H	O1	16	1259312	1259312	Missense_Mutation	SNP	G	G	A	CACNA1H:NM_021098:exon17:c.3644G>A:p.R1215H	45.87	NM_021098
PDILT	O1	16	20410533	20410533	Missense_Mutation	SNP	G	G	T	PDILT:NM_174924:exon2:c.90C>A:p.S30R	10.48	NM_174924
ARHGAP17	O1	16	24942383	24942383	Missense_Mutation	SNP	C	C	A	ARHGAP17:NM_001006634:exon19:c.2237G>T:p.G746V	11.02	NM_001006634
SRRM2	O1	16	2812492	2812492	Missense_Mutation	SNP	C	C	T	SRRM2:NM_016333:exon11:c.1963C>T:p.R655C	24.46	NM_016333
COG8	O1	16	69370591	69370591	Missense_Mutation	SNP	C	C	A	COG8:NM_032382:exon2:c.402G>T:p.E134D	40.85	NM_032382
KRTAP4-5	O1	17	39305769	39305769	Missense_Mutation	SNP	G	G	C	KRTAP4-5:NM_033188:exon1:c.251C>G:p.T84S	31.58	NM_033188
PIEZO2	O1	18	10704526	10704526	Missense_Mutation	SNP	C	C	T	PIEZO2:NM_022068:exon38:c.5785G>A:p.V1929M	20.43	NM_022068
ZNF440	O1	19	11943022	11943022	Missense_Mutation	SNP	T	T	C	ZNF440:NM_152357:exon4:c.1031T>C:p.I344T	60	NM_152357
IL12RB1	O1	19	18188352	18188352	Missense_Mutation	SNP	G	G	A	IL12RB1:NM_005535:exon5:c.523C>T:p.R175W	52.73	NM_005535
ZNF682	O1	19	20116980	20116980	Missense_Mutation	SNP	T	T	C	ZNF682:NM_033196:exon4:c.1331A>G:p.K444R	40.82	NM_033196
ZNF235	O1	19	44792897	44792897	Missense_Mutation	SNP	T	T	C	ZNF235:NM_004234:exon5:c.691A>G:p.K231E	11.81	NM_004234
APOC2	O1	19	45452463	45452470	Frame_Shift_Ins	DEL	TTTTTACT	TTTTTACT	-	APOC2:NM_000483:exon4:c.263_270delTTTTTACT:p.I88fs	15.65	NM_000483
SHANK1	O1	19	51219930	51219930	Missense_Mutation	SNP	G	G	A	SHANK1:NM_016148:exon1:c.247C>T:p.H83Y	19.74	NM_016148
RALGAPA2	O1	20	20571844	20571844	Missense_Mutation	SNP	G	G	A	RALGAPA2:NM_020343:exon17:c.2318C>T:p.P773L	16.98	NM_020343
IL1R2	O1	2	102642698	102642698	Missense_Mutation	SNP	G	G	A	IL1R2:NM_004633:exon8:c.1013G>A:p.R338H	28.65	NM_004633
PCNT	O1	21	47831841	47831841	Missense_Mutation	SNP	G	G	A	PCNT:NM_006031:exon28:c.5854G>A:p.A1952T	37.5	NM_006031
DNAH7	O1	2	196851900	196851900	Missense_Mutation	SNP	C	C	T	DNAH7:NM_018897:exon14:c.1644G>A:p.M548I	56	NM_018897
DNAH7	O1	2	196851911	196851911	Missense_Mutation	SNP	G	G	A	DNAH7:NM_018897:exon14:c.1633C>T:p.R545C	53.19	NM_018897
FAM117B	O1	2	203589694	203589694	Missense_Mutation	SNP	C	C	T	FAM117B:NM_173511:exon3:c.808C>T:p.R270C	28.13	NM_173511
ICA1L	O1	2	203684596	203684596	Missense_Mutation	SNP	C	C	T	ICA1L:NM_138468:exon6:c.386G>A:p.R129H	22.73	NM_138468
SPAG16	O1	2	214149289	214149289	Missense_Mutation	SNP	G	G	T	SPAG16:NM_024532:exon1:c.82G>T:p.D28Y	41.18	NM_024532
DMC1	O1	22	38934351	38934351	Missense_Mutation	SNP	G	G	A	DMC1:NM_007068:exon11:c.724C>T:p.R242W	31.53	NM_007068
VPS54	O1	2	64199317	64199317	Missense_Mutation	SNP	G	G	A	VPS54:NM_016516:exon4:c.440C>T:p.T147I	53.85	NM_016516
GCFC2	O1	2	75923413	75923413	Missense_Mutation	SNP	T	T	C	GCFC2:NM_003203:exon5:c.746A>G:p.N249S	49.02	NM_003203
DPPA2	O1	3	109031443	109031443	Missense_Mutation	SNP	C	C	T	DPPA2:NM_138815:exon3:c.130G>A:p.V44I	20	NM_138815
PLA1A	O1	3	119338415	119338415	Missense_Mutation	SNP	G	G	T	PLA1A:NM_015900:exon8:c.928G>T:p.V310L	11.69	NM_015900
NPHP3	O1	3	132402305	132402305	Missense_Mutation	SNP	C	C	G	NPHP3:NM_153240:exon25:c.3634G>C:p.G1212R	35.9	NM_153240
C3orf33	O1	3	155520356	155520356	Missense_Mutation	SNP	C	C	T	C3orf33:NM_001308229:exon2:c.139G>A:p.A47T	48.65	NM_001308229
ZNF385D	O1	3	21465505	21465505	Missense_Mutation	SNP	G	G	T	ZNF385D:NM_024697:exon7:c.904C>A:p.P302T	18.37	NM_024697
ROBO1	O1	3	78710404	78710404	Missense_Mutation	SNP	T	T	A	ROBO1:NM_002941:exon16:c.2096A>T:p.Q699L	28.57	NM_002941
ANK2	O1	4	114276243	114276243	Missense_Mutation	SNP	G	G	A	ANK2:NM_001148:exon38:c.6469G>A:p.E2157K	27.46	NM_001148
ADAM29	O1	4	175899075	175899075	Missense_Mutation	COMPLEX	CA	CA	TG	ADAM29:NM_014269:exon5:c.2399_2400delCAinsTG:p.T800M	24.21	NM_014269
ADAM29	O1	4	175899088	175899088	Missense_Mutation	SNP	G	G	T	ADAM29:NM_014269:exon5:c.2412G>T:p.R804S	28.42	NM_014269
ADAM29	O1	4	175899091	175899091	Missense_Mutation	SNP	A	A	T	ADAM29:NM_014269:exon5:c.2415A>T:p.Q805H	24.47	NM_014269
ADAM29	O1	4	175899102	175899102	Missense_Mutation	SNP	T	T	C	ADAM29:NM_014269:exon5:c.2426T>C:p.M809T	26.19	NM_014269
RASL11B	O1	4	53731859	53731859	Missense_Mutation	SNP	G	G	A	RASL11B:NM_023940:exon4:c.634G>A:p.E212K	29.69	NM_023940
CSN1S1	O1	4	70807771	70807771	Missense_Mutation	SNP	C	C	T	CSN1S1:NM_001890:exon13:c.350C>T:p.A117V	38.64	NM_001890
APC	O1	5	112174885	112174886	Frame_Shift_Ins	DEL	AA	AA	-	APC:NM_000038:exon16:c.3595_3596delAA:p.K1199fs	44.51	NM_000038

HNRNPA0	O1	5	137089172	137089180	In_Frame_Del	DEL	CGGCCGCCC	CGGCCGCCC	-	HNRNPA0:NM_006805:exon1:c.576_584delGGGCGGCCG:p.G193_R	24.24	NM_006805
SYNE1	O1	6	152644715	152644715	Missense_Mutation	SNP	C	C	T	SYNE1:NM_182961:exon82:c.15815G>A:p.R5272Q	12.82	NM_182961
KLF14	O1	7	130418281	130418281	Missense_Mutation	SNP	G	G	A	KLF14:NM_138693:exon1:c.580C>T:p.R194C	10.99	NM_138693
EPHB6	O1	7	142562154	142562154	Missense_Mutation	SNP	G	G	A	EPHB6:NM_004445:exon4:c.596G>A:p.R199Q	15.15	NM_004445
ELFN1	O1	7	1784840	1784840	Missense_Mutation	SNP	G	G	A	ELFN1:NM_001128636:exon2:c.608G>A:p.R203H	31.47	NM_001128636
BAG4	O1	8	38066607	38066607	Missense_Mutation	SNP	G	G	A	BAG4:NM_004874:exon4:c.710G>A:p.R237Q	10.58	NM_004874
VCPIP1	O1	8	67547410	67547410	Missense_Mutation	SNP	G	G	C	VCPIP1:NM_025054:exon3:c.2995C>G:p.P999A	10.67	NM_025054
OTUD6B	O1	8	92097062	92097062	Missense_Mutation	SNP	G	G	A	OTUD6B:NM_016023:exon7:c.938G>A:p.R313Q	44.59	NM_016023
FAM78A	O1	9	134136367	134136367	Missense_Mutation	SNP	G	G	A	FAM78A:NM_033387:exon2:c.694C>T:p.R232W	29.63	NM_033387
IFNA6	O1	9	21350544	21350544	Missense_Mutation	SNP	G	G	T	IFNA6:NM_021002:exon1:c.343C>A:p.Q115K	11.61	NM_021002
C9orf64	O1	9	86559744	86559744	Missense_Mutation	SNP	T	T	C	C9orf64:NM_032307:exon3:c.758A>G:p.K253R	45.61	NM_032307
KRAS	O2	12	25398284	25398284	Missense_Mutation	SNP	C	C	G	KRAS:NM_033360:exon2:c.35G>C:p.G12A	33.8	NM_033360
SLC2A14	O2	12	7982501	7982501	Missense_Mutation	SNP	C	C	T	SLC2A14:NM_001286237:exon4:c.488G>A:p.R163H	26.19	NM_001286237
CLSPN	O2	1	36217056	36217056	Missense_Mutation	SNP	C	C	T	CLSPN:NM_022111:exon10:c.1823G>A:p.R608Q	26.92	NM_022111
SCEL	O2	13	78133971	78133971	Missense_Mutation	SNP	G	G	A	SCEL:NM_144777:exon4:c.194G>A:p.R65Q	50.94	NM_144777
MYH6	O2	14	23870001	23870001	Missense_Mutation	SNP	G	G	A	MYH6:NM_002471:exon13:c.1327C>T:p.R443C	5.65	NM_002471
KCNB1	O2	20	47991025	47991025	Missense_Mutation	SNP	C	C	T	KCNB1:NM_004975:exon2:c.1072G>A:p.D358N	7.45	NM_004975
CHGB	O2	20	5903841	5903841	Missense_Mutation	SNP	G	G	A	CHGB:NM_001819:exon4:c.1051G>A:p.V351I	25	NM_001819
CAVIN2	O2	2	192711323	192711323	Missense_Mutation	SNP	G	G	A	CAVIN2:NM_004657:exon1:c.329C>T:p.T110M	12.12	NM_004657
PCBP1	O2	2	70315174	70315174	Missense_Mutation	SNP	T	T	A	PCBP1:NM_006196:exon1:c.299T>A:p.L100Q	9.71	NM_006196
KLHL6	O2	3	183226065	183226065	Missense_Mutation	SNP	C	C	T	KLHL6:NM_130446:exon3:c.691G>A:p.E231K	30.69	NM_130446
PPIP5K2	O2	5	102494919	102494919	Missense_Mutation	SNP	G	G	A	PPIP5K2:NM_001281471:exon17:c.1807G>A:p.G603S	25.49	NM_001281471
APC	O2	5	112175216	112175216	Nonsense_Mutation	SNP	G	G	T	APC:NM_000038:exon16:c.3925G>T:p.E1309*	90.59	NM_000038
ADAMTS16	O2	5	5262847	5262847	Nonsense_Mutation	SNP	C	C	T	ADAMTS16:NM_139056:exon18:c.2740C>T:p.R914*	35.59	NM_139056
ABCA13	O2	7	48314760	48314760	Missense_Mutation	SNP	C	C	T	ABCA13:NM_152701:exon17:c.5497C>T:p.R1833W	36.36	NM_152701
PAPPA	O2	9	118950362	118950362	Missense_Mutation	SNP	G	G	A	PAPPA:NM_002581:exon2:c.1345G>A:p.V449M	33.53	NM_002581
CA9	O2	9	35674204	35674204	Missense_Mutation	SNP	T	T	C	CA9:NM_001216:exon1:c.248T>C:p.L83P	12.68	NM_001216
GPR37L1	O2	1	202097525	202097527	In_Frame_Del	DEL	CTG	CTG	-	GPR37L1:NM_004767:exon2:c.1305_1307delCTG:p.C436del	10	NM_004767
OR5K4	O2	3	98073592	98073592	Frame_Shift_Ins	DEL	A	A	-	OR5K4:NM_001005517:exon1:c.901delA:p.I301fs	49.28	NM_001005517
ANKRD30A	O2	10	37488689	37488689	Missense_Mutation	SNP	G	G	C	ANKRD30A:NM_052997:exon30:c.2583G>C:p.K861N	54.55	NM_052997
KIF20B	O2	10	91487649	91487649	Missense_Mutation	SNP	A	A	T	KIF20B:NM_001284259:exon17:c.2267A>T:p.N756I	41.67	NM_001284259
KIF20B	O2	10	91488979	91488979	Missense_Mutation	SNP	A	A	T	KIF20B:NM_001284259:exon18:c.2366A>T:p.H789L	47.62	NM_001284259
PLCE1	O2	10	95995696	95995696	Missense_Mutation	SNP	G	G	T	PLCE1:NM_016341:exon7:c.2239G>T:p.D747Y	33.33	NM_016341
CWF19L2	O2	11	107286943	107286943	Missense_Mutation	SNP	C	C	T	CWF19L2:NM_152434:exon10:c.1609G>A:p.G537R	35.48	NM_152434
ANO5	O2	11	22239826	22239826	Missense_Mutation	SNP	G	G	A	ANO5:NM_213599:exon4:c.173G>A:p.R58Q	47.22	NM_213599
PSMD13	O2	11	251559	251559	Missense_Mutation	SNP	G	G	C	PSMD13:NM_175932:exon9:c.857G>C:p.R286P	40	NM_175932
FLG	O2	1	152280736	152280736	Missense_Mutation	SNP	T	T	C	FLG:NM_002016:exon3:c.6626A>G:p.H2209R	25	NM_002016
OR4A16	O2	11	55111584	55111584	Missense_Mutation	SNP	A	A	T	OR4A16:NM_001005274:exon1:c.908A>T:p.K303M	44.26	NM_001005274
UBQLN3	O2	11	5529465	5529465	Missense_Mutation	SNP	G	G	T	UBQLN3:NM_017481:exon2:c.1324C>A:p.P442T	36.14	NM_017481
SPTA1	O2	1	158584091	158584091	Missense_Mutation	SNP	A	A	G	SPTA1:NM_003126:exon49:c.6794T>C:p.I2265T	35.71	NM_003126
PRG4	O2	1	186273994	186273994	Missense_Mutation	SNP	C	C	T	PRG4:NM_005807:exon6:c.538C>T:p.R180W	51.11	NM_005807
CFH	O2	1	196695937	196695937	Missense_Mutation	SNP	G	G	T	CFH:NM_000186:exon14:c.2103G>T:p.W701C	12.35	NM_000186
TMCC2	O2	1	205241235	205241235	Missense_Mutation	SNP	G	G	A	TMCC2:NM_014858:exon5:c.2113G>A:p.V705M	24.29	NM_014858
SRRM4	O2	12	119563214	119563214	Missense_Mutation	SNP	C	C	T	SRRM4:NM_194286:exon7:c.544C>T:p.R182C	16.13	NM_194286
PIK3C2G	O2	12	18435399	18435401	In_Frame_Del	DEL	CCC	CCC	-	PIK3C2G:NM_001288772:exon2:c.385_387delCCC:p.P129del	36.9	NM_001288772
PIK3C2G	O2	12	18435452	18435452	Missense_Mutation	SNP	C	C	T	PIK3C2G:NM_001288772:exon2:c.437C>T:p.P146L	35.62	NM_001288772
SOX5	O2	12	23716305	23716305	Missense_Mutation	SNP	C	C	T	SOX5:NM_006940:exon11:c.1375G>A:p.A459T	26.4	NM_006940
MRPS35	O2	12	27867727	27867727	Missense_Mutation	SNP	G	G	A	MRPS35:NM_021821:exon2:c.127G>A:p.G43R	40.58	NM_021821
MBD6	O2	12	57918525	57918525	Missense_Mutation	SNP	T	T	C	MBD6:NM_052897:exon4:c.136T>C:p.S46P	36.92	NM_052897
CD163L1	O2	12	7548793	7548793	Missense_Mutation	SNP	C	C	T	CD163L1:NM_001297650:exon8:c.1978G>A:p.V660I	36.8	NM_001297650
WNK1	O2	12	970483	970483	Missense_Mutation	SNP	A	A	G	WNK1:NM_213655:exon7:c.1925A>G:p.Y642C	34.62	NM_213655
PROSER1	O2	13	39600487	39600489	In_Frame_Del	DEL	ATC	ATC	-	PROSER1:NM_025138:exon6:c.405_407delGAT:p.M135del	10.99	NM_025138
SETDB2	O2	13	50057097	50057097	Missense_Mutation	SNP	G	G	A	SETDB2:NM_031915:exon10:c.1417G>A:p.V473M	59.32	NM_031915
KLF12	O2	13	74339129	74339129	Missense_Mutation	SNP	G	G	A	KLF12:NM_007249:exon6:c.815C>T:p.P272L	28.77	NM_007249
CLN5	O2	13	77566090	77566090	Missense_Mutation	SNP	C	C	T	CLN5:NM_006493:exon1:c.4C>T:p.R2C	42.86	NM_006493
OXGR1	O2	13	97639622	97639622	Missense_Mutation	SNP	C	C	T	OXGR1:NM_080818:exon4:c.392G>A:p.R131H	52.76	NM_080818
PLD4	O2	14	105398401	105398401	Missense_Mutation	SNP	G	G	A	PLD4:NM_001308174:exon9:c.1132G>A:p.V378M	46.15	NM_001308174
C14orf39	O2	14	60932724	60932724	Missense_Mutation	SNP	G	G	T	C14orf39:NM_174978:exon11:c.945C>A:p.D315E	46.15	NM_174978
PHGR1	O2	15	40648396	40648396	Missense_Mutation	COMPLEX	TGG	TGG	CCA	PHGR1:NM_001145643:exon3:c.141_143delTGGinsCCA:p.G48H	21.05	NM_001145643
KNL1	O2	15	40920871	40920872	Frame_Shift_Ins	INS	-	-	G	KNL1:NM_170589:exon13:c.5659dupG:p.E1887fs	24.11	NM_170589
ADAMTSL3	O2	15	84611460	84611460	Nonsense_Mutation	SNP	C	C	T	ADAMTSL3:NM_207517:exon18:c.2230C>T:p.R744*	38.89	NM_207517
SCNN1B	O2	16	23391982	23391982	Missense_Mutation	SNP	G	G	C	SCNN1B:NM_000336:exon13:c.1783G>C:p.A595P	22.58	NM_000336
WLS	O2	1	68614286	68614286	Missense_Mutation	SNP	C	C	T	WLS:NM_001002292:exon7:c.1024G>A:p.V342I	29.03	NM_001002292
MYO15A	O2	17	18024806	18024806	Missense_Mutation	SNP	G	G	A	MYO15A:NM_016239:exon2:c.2692G>A:p.G898R	28.26	NM_016239
UTP6	O2	17	30222002	30222002	Missense_Mutation	SNP	T	T	C	UTP6:NM_018428:exon3:c.206A>G:p.Q69R	62.5	NM_018428
ARRB2	O2	17	4619845	4619845	Missense_Mutation	SNP	G	G	A	ARRB2:NM_001257328:exon5:c.299G>A:p.R100H	31.88	NM_001257328
AXIN2	O2	17	63554666	63554666	Missense_Mutation	SNP	G	G	A	AXIN2:NM_004655:exon2:c.73C>T:p.P25S	64.29	NM_004655
ABCA6	O2	17	67125840	67125840	Missense_Mutation	SNP	C	C	T	ABCA6:NM_080284:exon7:c.844G>A:p.V282I	22.92	NM_080284
CTC1	O2	17	8134748	8134748	Frame_Shift_Ins	DEL	A	A	-	CTC1:NM_025099:exon15:c.2515delT:p.S839fs	21.05	NM_025099
COL24A1	O2	1	86512536	86512536	Missense_Mutation	SNP	C	C	T	COL24A1:NM_152890:exon12:c.1922G>A:p.R641H	40.74	NM_152890
CBLN2	O2	18	70209101	70209101	Missense_Mutation	SNP	G	G	A	CBLN2:NM_182511:exon3:c.295C>T:p.R99W	32.1	NM_182511
SALL3	O2	18	76754818	76754818	Missense_Mutation	SNP	G	G	A	SALL3:NM_171999:exon2:c.2827G>A:p.A943T	12.77	NM_171999
ZNF728	O2	19	23159486	23159486	Missense_Mutation	SNP	T	T	C	ZNF728:NM_001267716:exon4:c.653A>G:p.Y218C	60	NM_001267716
TMEM147	O2	19	36038036	36038036	Missense_Mutation	SNP	G	G	A	TMEM147:NM_032635:exon6:c.445G>A:p.A149T	27.12	NM_032635
NLRP2	O2	19	55494753	55494753	Missense_Mutation	SNP	G	G	A	NLRP2:NM_017852:exon6:c.1687G>A:p.E563K	31.71	NM_017852
ZSCAN5B	O2	19	56703269	56703269	Missense_Mutation	SNP	G	G	A	ZSCAN5B:NM_001080456:exon2:c.538C>T:p.R180C	31.94	NM_001080456
RAD21L1	O2	20	1214814	1214814	Missense_Mutation	SNP	A	A	C	RAD21L1:NM_001136566:exon5:c.454A>C:p.I152L	39.29	NM_001136566
ROCK2	O2	2	11359120	11359120	Missense_Mutation	SNP	G	G	T	ROCK2:NM_004850:exon10:c.1292C>A:p.T431N	50	NM_004850
LIPI	O2	21	15524921	15524921	Missense_Mutation	SNP	C	C	T	LIPI:NM_198996:exon8:c.1154G>A:p.G385E	39.53	NM_198996
RBM11	O2	21	15596772	15596772	Missense_Mutation	SNP	T	T	G	RBM11:NM_001320602:exon4:c.346T>G:p.L116V	50	NM_001320602
NMI	O2	2	152139416	152139416	Missense_Mutation	SNP	G	G	A	NMI:NM_004688:exon2:c.47C>T:p.S16L	47.27	NM_004688
VRK2	O2	2	58316814	58316814	Missense_Mutation	SNP	A	A	G	VRK2:NM_006296:exon7:c.499A>G:p.I167V	46.15	NM_006296

ANKRD36	O2	2	97877292	97877292	Missense_Mutation	SNP	C	C	T	ANKRD36:NM_001164315:exon57:c.3377C>T:p.P1126L	57.14	NM_001164315
TIMP4	O2	3	12198364	12198364	Missense_Mutation	SNP	C	C	T	TIMP4:NM_003256:exon3:c.308G>A:p.G103D	39	NM_003256
ZBBX	O2	3	167051769	167051769	Missense_Mutation	SNP	T	T	C	ZBBX:NM_001199201:exon10:c.533A>G:p.K178R	58.82	NM_001199201
OR5H15	O2	3	97888438	97888438	Missense_Mutation	SNP	G	G	C	OR5H15:NM_001005515:exon1:c.895G>C:p.V299L	40.91	NM_001005515
OR5H6	O2	3	97983711	97983711	Missense_Mutation	SNP	T	T	C	OR5H6:NM_001005479:exon1:c.583T>C:p.C195R	58.33	NM_001005479
CCDC110	O2	4	186380243	186380243	Missense_Mutation	SNP	A	A	C	CCDC110:NM_152775:exon6:c.1498T>G:p.Y500D	44.58	NM_152775
KLKB1	O2	4	187178473	187178473	Missense_Mutation	SNP	G	G	A	KLKB1:NM_000892:exon14:c.1679G>A:p.R560Q	42.37	NM_000892
CNGA1	O2	4	47945286	47945286	Nonsense_Mutation	SNP	C	C	A	CNGA1:NM_001142564:exon7:c.568G>T:p.E190*	11.76	NM_001142564
FRAS1	O2	4	79367890	79367890	Missense_Mutation	SNP	G	G	A	FRAS1:NM_025074:exon43:c.5866G>A:p.D1956N	31.03	NM_025074
STPG2	O2	4	99027184	99027184	Missense_Mutation	SNP	T	T	C	STPG2:NM_174952:exon5:c.532A>G:p.I178V	36.67	NM_174952
SPINK5	O2	5	147499645	147499645	Missense_Mutation	SNP	G	G	A	SPINK5:NM_006846:exon25:c.2387G>A:p.R796Q	30.3	NM_006846
NIPBL	O2	5	37020625	37020625	Missense_Mutation	SNP	T	T	C	NIPBL:NM_133433:exon26:c.5075T>C:p.M1692T	23.53	NM_133433
ADAMTS6	O2	5	64756126	64756126	Missense_Mutation	SNP	C	C	T	ADAMTS6:NM_197941:exon4:c.502G>A:p.E168K	30.26	NM_197941
POC5	O2	5	75001582	75001582	Missense_Mutation	SNP	A	A	G	POC5:NM_001099271:exon4:c.254T>C:p.I85T	38.64	NM_001099271
TRDN	O2	6	123696766	123696766	Missense_Mutation	SNP	G	G	T	TRDN:NM_006073:exon19:c.1257C>A:p.D419E	39.29	NM_006073
RSPH3	O2	6	159399348	159399348	Missense_Mutation	SNP	A	A	G	RSPH3:NM_031924:exon7:c.1316T>C:p.M439T	54.84	NM_031924
MUC22	O2	6	30996839	30996839	Missense_Mutation	SNP	G	G	A	MUC22:NM_001322469:exon3:c.3640G>A:p.E1214K	15.19	NM_001322469
IMPG1	O2	6	76631823	76631823	Missense_Mutation	SNP	C	C	T	IMPG1:NM_001563:exon17:c.2377G>A:p.D793N	45.45	NM_001563
MICALL2	O2	7	1478611	1478611	Missense_Mutation	SNP	G	G	A	MICALL2:NM_182924:exon10:c.1987C>T:p.R663C	18.6	NM_182924
PKD1L1	O2	7	47917119	47917119	Missense_Mutation	SNP	C	C	T	PKD1L1:NM_138295:exon22:c.3631G>A:p.V1211I	24	NM_138295
ZNF735	O2	7	63680098	63680098	Missense_Mutation	COMPLEX	CC	CC	TA	ZNF735:NM_001159524:exon4:c.669_670delCCinsTA:p.H224N	69.23	NM_001159524
GUSB	O2	7	65441045	65441045	Missense_Mutation	SNP	G	G	A	GUSB:NM_000181:exon5:c.869C>T:p.P290L	26.89	NM_000181
ABCB4	O2	7	87074281	87074282	Frame_Shift_Ins	INS	-	-	A	ABCB4:NM_018849:exon10:c.1015dupT:p.S339fs	10.29	NM_018849
PUF60	O2	8	144899294	144899294	Missense_Mutation	SNP	G	G	C	PUF60:NM_078480:exon11:c.1166C>G:p.P389R	42.86	NM_078480
FOCAD	O2	9	20819889	20819889	Missense_Mutation	SNP	G	G	T	FOCAD:NM_017794:exon14:c.1550G>T:p.G517V	35.85	NM_017794
RORB	O2	9	77257699	77257699	Missense_Mutation	SNP	A	A	G	RORB:NM_006914:exon4:c.605A>G:p.Q202R	31.39	NM_006914
ZNF75D	O2	X	134421241	134421241	Missense_Mutation	SNP	C	C	T	ZNF75D:NM_007131:exon7:c.1361G>A:p.C454Y	22.81	NM_007131
WSCD2	O3	12	108603974	108603974	Missense_Mutation	SNP	G	G	A	WSCD2:NM_014653:exon4:c.574G>A:p.E192K	13.33	NM_014653
OBSCN	O3	1	228444454	228444454	Missense_Mutation	SNP	C	C	T	OBSCN:NM_001271223:exon16:c.4688C>T:p.T1563M	49.02	NM_001271223
WDR59	O3	16	74955991	74955991	Missense_Mutation	SNP	T	T	C	WDR59:NM_030581:exon10:c.740A>G:p.N247S	7.69	NM_030581
APC	O3	5	112173917	112173917	Nonsense_Mutation	SNP	C	C	T	APC:NM_000038:exon16:c.2626C>T:p.R876*	39.16	NM_000038
APC	O3	5	112175618	112175618	Frame_Shift_Ins	DEL	C	C	-	APC:NM_000038:exon16:c.4328delC:p.P1443fs	33.33	NM_000038
PITRM1	O3	10	3208567	3208568	Frame_Shift_Ins	INS	-	-	GCACGCTAGGGAAGAGAGAGGAATG	PITRM1:NM_001242307:exon4:c.271_272insCATTCCTCTCTCTTCCCTAGCGTGC:p.Q91fs	27.27	NM_001242307
FAM208B	O3	10	5804509	5804509	Missense_Mutation	SNP	C	C	T	FAM208B:NM_017782:exon20:c.7189C>T:p.P2397S	39.47	NM_017782
KIF20B	O3	10	91488979	91488979	Missense_Mutation	SNP	A	A	T	KIF20B:NM_001284259:exon18:c.2366A>T:p.H789L	42.86	NM_001284259
CEP164	O3	11	117262952	117262952	Missense_Mutation	SNP	C	C	T	CEP164:NM_014956:exon18:c.2294C>T:p.T765M	30.77	NM_014956
IGSF3	O3	1	117122288	117122288	Missense_Mutation	SNP	G	G	C	IGSF3:NM_001542:exon10:c.3120C>G:p.D1040E	22.73	NM_001542
LMNA	O3	1	156108328	156108328	Missense_Mutation	SNP	C	C	T	LMNA:NM_170707:exon11:c.1748C>T:p.S583L	32.26	NM_170707
DGAT2	O3	11	75508371	75508371	Missense_Mutation	SNP	G	G	A	DGAT2:NM_032564:exon6:c.803G>A:p.R268H	35	NM_032564
IKBKE	O3	1	206669465	206669465	Missense_Mutation	SNP	C	C	T	IKBKE:NM_014002:exon22:c.2138C>T:p.P713L	50	NM_014002
DPF3	O3	14	73137968	73137968	Missense_Mutation	SNP	G	G	A	DPF3:NM_001280544:exon9:c.1115C>T:p.T372M	11.81	NM_001280544
C15orf39	O3	15	75499490	75499490	Missense_Mutation	SNP	C	C	G	C15orf39:NM_015492:exon2:c.1101C>G:p.C367W	43.21	NM_015492
DNAH3	O3	16	20990740	20990740	Missense_Mutation	SNP	G	G	A	DNAH3:NM_017539:exon50:c.7988C>T:p.T2663M	38.98	NM_017539
CDH5	O3	16	66436606	66436606	Missense_Mutation	SNP	C	C	T	CDH5:NM_001795:exon12:c.1889C>T:p.A630V	11.7	NM_001795
MYH2	O3	17	10424632	10424632	Missense_Mutation	SNP	G	G	A	MYH2:NM_017534:exon40:c.5791C>T:p.R1931W	22.22	NM_017534
COX10	O3	17	14110412	14110412	Missense_Mutation	SNP	G	G	A	COX10:NM_001303:exon7:c.1214G>A:p.R405H	26.58	NM_001303
UNC45B	O3	17	33495289	33495289	Missense_Mutation	SNP	G	G	A	UNC45B:NM_173167:exon9:c.1361G>A:p.R454H	43.14	NM_173167
COG1	O3	17	71197890	71197890	Missense_Mutation	SNP	C	C	A	COG1:NM_018714:exon7:c.1924C>A:p.P642T	31.67	NM_018714
ATP8B3	O3	19	1785539	1785539	Missense_Mutation	SNP	C	C	T	ATP8B3:NM_138813:exon26:c.3322G>A:p.A1108T	24.18	NM_138813
SPTBN4	O3	19	41018705	41018705	Missense_Mutation	SNP	C	C	T	SPTBN4:NM_020971:exon14:c.2009C>T:p.A670V	66.67	NM_020971
MADCAM1	O3	19	501701	501701	Missense_Mutation	SNP	G	G	A	MADCAM1:NM_130760:exon4:c.700G>A:p.D234N	44.74	NM_130760
MADCAM1	O3	19	501725	501725	Missense_Mutation	SNP	G	G	A	MADCAM1:NM_130760:exon4:c.724G>A:p.D242N	54.55	NM_130760
TSKS	O3	19	50249923	50249940	In_Frame_Del	DEL	CCTCCGGCTTCTGCTTCT	CCTCCGGCTTCTGCTTCT	-	TSKS:NM_021733:exon6:c.779_796delAGAAGCAGAAGCCGGAGG:p.E260_E265del	15.63	NM_021733
SPTLC3	O3	20	13145453	13145453	Missense_Mutation	SNP	G	G	A	SPTLC3:NM_018327:exon12:c.1598G>A:p.R533Q	22.37	NM_018327
NCOA6	O3	20	33338227	33338227	Missense_Mutation	SNP	T	T	C	NCOA6:NM_014071:exon9:c.1771A>G:p.M591V	11.9	NM_014071
RTEL1	O3	20	62298844	62298844	Missense_Mutation	SNP	C	C	T	RTEL1:NM_001283009:exon8:c.637C>T:p.R213W	13.04	NM_001283009
GLI2	O3	2	121726395	121726395	Missense_Mutation	SNP	G	G	A	GLI2:NM_005270:exon5:c.749G>A:p.R250H	26.6	NM_005270
SAP130	O3	2	128773988	128773988	Missense_Mutation	SNP	G	G	A	SAP130:NM_001145928:exon4:c.560C>T:p.P187L	36.49	NM_001145928
URB1	O3	21	33694176	33694176	Missense_Mutation	SNP	G	G	A	URB1:NM_014825:exon34:c.5419C>T:p.R1807W	42.25	NM_014825
P2RX6	O3	22	21377288	21377288	Nonsense_Mutation	SNP	G	G	A	P2RX6:NM_005446:exon5:c.521G>A:p.W174*	49.21	NM_005446
TMEM247	O3	2	46707851	46707851	Missense_Mutation	SNP	G	G	A	TMEM247:NM_001145051:exon2:c.425G>A:p.R142Q	55.26	NM_001145051
CCDC80	O3	3	112358175	112358175	Missense_Mutation	SNP	G	G	A	CCDC80:NM_199511:exon2:c.578C>T:p.A193V	43.82	NM_199511
WNT5A	O3	3	55504410	55504410	Missense_Mutation	SNP	C	C	T	WNT5A:NM_003392:exon5:c.853G>A:p.V285I	46.15	NM_003392
C4orf47	O3	4	186361716	186361716	Missense_Mutation	SNP	G	G	A	C4orf47:NM_001114357:exon5:c.557G>A:p.R186H	17.65	NM_001114357
VARS	O3	6	31762844	31762844	Missense_Mutation	SNP	G	G	T	VARS:NM_006295:exon2:c.151C>A:p.P51T	12	NM_006295
IRF4	O3	6	395933	395933	Missense_Mutation	SNP	C	C	A	IRF4:NM_002460:exon4:c.490C>A:p.Q164K	30	NM_002460
RBMXL3	O3	X	114425196	114425196	Missense_Mutation	SNP	G	G	A	RBMXL3:NM_001145346:exon1:c.1192G>A:p.D398N	44.33	NM_001145346
TENM1	O3	X	123518158	123518158	Missense_Mutation	SNP	C	C	T	TENM1:NM_001163278:exon30:c.6623G>A:p.R2208H	12.9	NM_001163278
EGR2	O4	10	64574154	64574154	Missense_Mutation	SNP	C	C	T	EGR2:NM_000399:exon2:c.244G>A:p.V82I	21.58	NM_000399
AMOTL1	O4	11	94583333	94583333	Missense_Mutation	SNP	G	G	A	AMOTL1:NM_130847:exon7:c.1703G>A:p.R568Q	23.81	NM_130847
STAB2	O4	12	104105290	104105290	Missense_Mutation	SNP	G	G	A	STAB2:NM_017564:exon40:c.4330G>A:p.A1444T	45.35	NM_017564
KRAS	O4	12	25398285	25398285	Missense_Mutation	SNP	C	C	A	KRAS:NM_033360:exon2:c.34G>T:p.G12C	13.64	NM_033360
FBXW7	O4	4	153245447	153245447	Missense_Mutation	SNP	A	A	G	FBXW7:NM_033632:exon11:c.1744T>C:p.S582P	19.35	NM_033632
APC	O4	5	112173917	112173917	Nonsense_Mutation	SNP	C	C	T	APC:NM_000038:exon16:c.2626C>T:p.R876*	38.3	NM_000038
APC	O4	5	112175639	112175639	Nonsense_Mutation	SNP	C	C	T	APC:NM_000038:exon16:c.4348C>T:p.R1450*	60	NM_000038
INA	O4	10	105037815	105037815	Missense_Mutation	SNP	G	G	A	INA:NM_032727:exon1:c.847G>A:p.A283T	14.94	NM_032727
SFRP5	O4	10	99531229	99531229	Missense_Mutation	SNP	A	A	G	SFRP5:NM_003015:exon1:c.362T>C:p.I121T	25	NM_003015
CNTN5	O4	11	100061886	100061886	Missense_Mutation	SNP	C	C	T	CNTN5:NM_014361:exon14:c.1609C>T:p.R537W	41.56	NM_014361
KDELC2	O4	11	108350085	108350085	Missense_Mutation	SNP	A	A	C	KDELC2:NM_153705:exon6:c.1236T>G:p.H412Q	35.71	NM_153705
ARHGEF12	O4	11	120351125	120351125	Missense_Mutation	SNP	A	A	G	ARHGEF12:NM_015313:exon38:c.4223A>G:p.N1408S	34.38	NM_015313
INTS11	O4	1	1256383	1256383	Missense_Mutation	SNP	T	T	C	INTS11:NM_001256456:exon4:c.137A>G:p.N46S	11.32	NM_001256456

IVL	O4	1	152883680	152883680	Missense_Mutation	SNP	A	A	T	IVL:NM_005547:exon2:c.1407A>T:p.Q469H	56.6	NM_005547
OR51B2	O4	11	5345158	5345158	Missense_Mutation	SNP	T	T	C	OR51B2:NM_033180:exon1:c.370A>G:p.I124V	44.68	NM_033180
NES	O4	1	156642746	156642748	In_Frame_Del	DEL	GAG	GAG	-	NES:NM_006617:exon4:c.1232_1234delCTC:p.P411del	43.75	NM_006617
PTPRC	O4	1	198672442	198672442	Missense_Mutation	SNP	C	C	G	PTPRC:NM_002838:exon7:c.599C>G:p.A200G	18.31	NM_002838
ELF3	O4	1	201983002	201983003	Frame_Shift_Ins	INS	-	-	C	ELF3:NM_004433:exon8:c.853dupC:p.H285fs	20.69	NM_004433
PITPNM2	O4	12	123485694	123485694	Missense_Mutation	SNP	C	C	G	PITPNM2:NM_020845:exon8:c.1060G>C:p.D354H	39.34	NM_020845
SMYD2	O4	1	214504326	214504326	Missense_Mutation	SNP	G	G	A	SMYD2:NM_020197:exon9:c.850G>A:p.D284N	38.95	NM_020197
CAPZA3	O4	12	18891215	18891215	Missense_Mutation	SNP	G	G	A	CAPZA3:NM_033328:exon1:c.13G>A:p.V5M	41.18	NM_033328
CACNA2D4	O4	12	1993428	1993428	Nonsense_Mutation	SNP	C	C	T	CACNA2D4:NM_172364:exon12:c.1332G>A:p.W444*	28.3	NM_172364
NPAS3	O4	14	34269079	34269079	Missense_Mutation	SNP	C	C	A	NPAS3:NM_001164749:exon12:c.1566C>A:p.N522K	24.16	NM_001164749
ZYG11B	O4	1	53282301	53282301	Missense_Mutation	SNP	C	C	T	ZYG11B:NM_024646:exon13:c.2044C>T:p.P682S	25.86	NM_024646
MEFV	O4	16	3306524	3306524	Missense_Mutation	SNP	C	C	T	MEFV:NM_000243:exon1:c.64G>A:p.E22K	24	NM_000243
SLC2A7	O4	1	9073610	9073610	Missense_Mutation	SNP	C	C	T	SLC2A7:NM_207420:exon8:c.988G>A:p.V330I	28.21	NM_207420
FCHO1	O4	19	17875219	17875219	Missense_Mutation	SNP	C	C	T	FCHO1:NM_001161357:exon6:c.155C>T:p.A52V	34.62	NM_001161357
GMIP	O4	19	19747825	19747825	Missense_Mutation	SNP	T	T	C	GMIP:NM_016573:exon12:c.1138A>G:p.I380V	65	NM_016573
MADCAM1	O4	19	501719	501719	Missense_Mutation	SNP	T	T	C	MADCAM1:NM_130760:exon4:c.718T>C:p.S240P	65.79	NM_130760
ZNF446	O4	19	58991828	58991828	Missense_Mutation	SNP	C	C	T	ZNF446:NM_017908:exon7:c.1088C>T:p.P363L	43.93	NM_017908
TGM6	O4	20	2384368	2384368	Missense_Mutation	SNP	G	G	A	TGM6:NM_198994:exon9:c.1235G>A:p.R412H	54.35	NM_198994
FAM83D	O4	20	37555158	37555158	Missense_Mutation	SNP	A	A	G	FAM83D:NM_030919:exon1:c.73A>G:p.T25A	47.37	NM_030919
RIPOR3	O4	20	49219025	49219025	Missense_Mutation	SNP	G	G	A	RIPOR3:NM_001290268:exon13:c.1243C>T:p.P415S	33.33	NM_001290268
NEB	O4	2	152520339	152520339	Missense_Mutation	SNP	C	C	A	NEB:NM_001271208:exon45:c.5486G>T:p.G1829V	13.43	NM_001271208
GIGYF2	O4	2	233660881	233660881	Missense_Mutation	SNP	A	A	G	GIGYF2:NM_001103147:exon16:c.1652A>G:p.H551R	46.07	NM_001103147
SYCE3	O4	22	50994763	50994763	Missense_Mutation	SNP	G	G	T	SYCE3:NM_001123225:exon2:c.72C>A:p.D24E	25.23	NM_001123225
BIRC6	O4	2	32823018	32823018	Nonsense_Mutation	SNP	C	C	T	BIRC6:NM_016252:exon69:c.13813C>T:p.R4605*	25.71	NM_016252
POLE4	O4	2	75185873	75185873	Missense_Mutation	SNP	G	G	A	POLE4:NM_019896:exon1:c.67G>A:p.A23T	28.57	NM_019896
CTNNA2	O4	2	80782889	80782889	Missense_Mutation	SNP	C	C	T	CTNNA2:NM_001282597:exon12:c.1612C>T:p.R538W	24.49	NM_001282597
NLGN1	O4	3	173998554	173998554	Missense_Mutation	SNP	C	C	T	NLGN1:NM_014932:exon7:c.1933C>T:p.P645S	47.4	NM_014932
FBXW7	O4	4	153258998	153258998	Nonsense_Mutation	SNP	C	C	A	FBXW7:NM_033632:exon5:c.817G>T:p.E273*	25	NM_033632
TMPRSS11F	O4	4	68930524	68930524	Missense_Mutation	SNP	C	C	A	TMPRSS11F:NM_207407:exon8:c.894G>T:p.Q298H	12.5	NM_207407
CWC27	O4	5	64097145	64097145	Missense_Mutation	SNP	C	C	G	CWC27:NM_005869:exon9:c.766C>G:p.P256A	23.81	NM_005869
TAGAP	O4	6	159457709	159457709	Missense_Mutation	SNP	G	G	A	TAGAP:NM_054114:exon10:c.1346C>T:p.S449L	30.26	NM_054114
FLNC	O4	7	128490905	128490905	Missense_Mutation	SNP	A	A	T	FLNC:NM_001458:exon33:c.5447A>T:p.D1816V	19.4	NM_001458
ETV1	O4	7	13940436	13940436	Missense_Mutation	SNP	T	T	C	ETV1:NM_004956:exon13:c.1139A>G:p.N380S	44.78	NM_004956
KEL	O4	7	142658528	142658528	Missense_Mutation	SNP	C	C	T	KEL:NM_000420:exon3:c.142G>A:p.V48M	60.61	NM_000420
MIOS	O4	7	7613204	7613204	Missense_Mutation	SNP	A	A	C	MIOS:NM_019005:exon4:c.1098A>C:p.L366F	32.14	NM_019005
PCLO	O4	7	82784832	82784832	Missense_Mutation	SNP	C	C	G	PCLO:NM_033026:exon2:c.1125G>C:p.Q375H	47.27	NM_033026
ODF1	O4	8	103573006	103573006	Missense_Mutation	SNP	G	G	A	ODF1:NM_024410:exon2:c.647G>A:p.S216N	59.69	NM_024410
C9orf84	O4	9	114500617	114500617	Missense_Mutation	SNP	A	A	G	C9orf84:NM_173521:exon10:c.1168T>C:p.S390P	40.74	NM_173521
TUB	P1	11	8122144	8122144	Missense_Mutation	SNP	G	G	A	TUB:NM_003320:exon11:c.1376G>A:p.R459H	50	NM_003320
ABCC12	P1	16	48149472	48149472	Missense_Mutation	SNP	G	G	A	ABCC12:NM_033226:exon13:c.1843C>T:p.R615C	18.1	NM_033226
GJC1	P1	17	42882687	42882687	Missense_Mutation	SNP	G	G	A	GJC1:NM_005497:exon3:c.499C>T:p.R167W	31.82	NM_005497
TP53	P1	17	7578203	7578203	Missense_Mutation	SNP	C	C	T	TP53:NM_000546:exon6:c.646G>A:p.V216M	56.1	NM_000546
SMAD4	P1	18	48593466	48593466	Missense_Mutation	SNP	C	C	T	SMAD4:NM_005359:exon10:c.1217C>T:p.A406V	43.24	NM_005359
NRXN1	P1	2	50850603	50850603	Missense_Mutation	SNP	G	G	T	NRXN1:NM_001135659:exon7:c.1082C>A:p.A361D	7.91	NM_001135659
PRR23C	P1	3	138762733	138762733	Missense_Mutation	SNP	G	G	A	PRR23C:NM_001134657:exon1:c.730C>T:p.R244C	29.06	NM_001134657
APC	P1	5	112175273	112175273	Nonsense_Mutation	SNP	C	C	T	APC:NM_000038:exon16:c.3982C>T:p.Q1328*	27.59	NM_000038
CFB	P1	6	31915244	31915244	Missense_Mutation	SNP	C	C	T	CFB:NM_001710:exon4:c.604C>T:p.R202W	39.68	NM_001710
XPC	P1	3	14219966	14219968	In_Frame_Del	DEL	CCT	CCT	-	XPC:NM_004628:exon1:c.101_103delAGG:p.E34del	14.29	NM_004628
PRDM8	P1	4	81123250	81123252	In_Frame_Del	DEL	CAG	CAG	-	PRDM8:NM_020226:exon10:c.649_651delCAG:p.Q217del	11.25	NM_020226
PODXL	P1	7	131241030	131241035	In_Frame_Del	DEL	GGCGAC	GGCGAC	-	PODXL:NM_001018111:exon1:c.84_89delGTCGCC:p.S29_P30del	50	NM_001018111
ITIH2	P1	10	7773844	7773844	Missense_Mutation	SNP	C	C	T	ITIH2:NM_002216:exon13:c.1532C>T:p.T511M	26.79	NM_002216
SLC6A5	P1	11	20622978	20622978	Missense_Mutation	SNP	G	G	A	SLC6A5:NM_004211:exon2:c.307G>A:p.A103T	15.38	NM_004211
FLG	P1	1	152282560	152282560	Missense_Mutation	SNP	T	T	C	FLG:NM_002016:exon3:c.4802A>G:p.E1601G	19.51	NM_002016
MS4A8	P1	11	60482518	60482518	Missense_Mutation	SNP	G	G	A	MS4A8:NM_031457:exon6:c.559G>A:p.V187M	25.71	NM_031457
SLC9C2	P1	1	173493208	173493208	Frame_Shift_Ins	DEL	A	A	-	SLC9C2:NM_178527:exon21:c.2540delT:p.L847fs	30.36	NM_178527
OR2AT4	P1	11	74800712	74800712	Missense_Mutation	SNP	A	A	T	OR2AT4:NM_001005285:exon1:c.47T>A:p.F16Y	11.11	NM_001005285
KLRC4	P1	12	10560957	10560957	Missense_Mutation	SNP	T	T	C	KLRC4:NM_013431:exon3:c.311A>G:p.N104S	46.15	NM_013431
SLCO1B3	P1	12	21028266	21028267	Frame_Shift_Ins	INS	-	-	T	SLCO1B3:NM_019844:exon9:c.833dupT:p.P280fs	27.81	NM_019844
CCDC91	P1	12	28605426	28605426	Missense_Mutation	SNP	G	G	A	CCDC91:NM_018318:exon10:c.940G>A:p.V314M	49.06	NM_018318
RYR2	P1	1	237947838	237947838	Missense_Mutation	SNP	G	G	A	RYR2:NM_001035:exon90:c.12826G>A:p.V4276M	34.44	NM_001035
KCNA5	P1	12	5154893	5154893	Missense_Mutation	SNP	C	C	T	KCNA5:NM_002234:exon1:c.1580C>T:p.T527M	10.99	NM_002234
KRT2	P1	12	53045603	53045604	In_Frame_Ins	INS	-	-	AAGCCGCTGCCTCCTCCA	KRT2:NM_000423:exon1:c.323_324insTGGAGGAGGCAGCGGCTT:p.F108_S109insGGGSGF	10.26	NM_000423
KRT1	P1	12	53069243	53069243	Missense_Mutation	SNP	T	T	C	KRT1:NM_006121:exon9:c.1669A>G:p.S557G	22.33	NM_006121
PTPRQ	P1	12	80838175	80838175	Missense_Mutation	SNP	C	C	T	PTPRQ:NM_001145026:exon1:c.50C>T:p.T17I	39.02	NM_001145026
ZMYM6	P1	1	35453270	35453273	Frame_Shift_Ins	DEL	AAGA	AAGA	-	ZMYM6:NM_007167:exon16:c.3410_3413delTCTT:p.F1137fs	32.26	NM_007167
OR4N2	P1	14	20296298	20296298	Missense_Mutation	SNP	G	G	A	OR4N2:NM_001004723:exon2:c.691G>A:p.E231K	36.24	NM_001004723
C14orf39	P1	14	60903757	60903757	Missense_Mutation	SNP	G	G	A	C14orf39:NM_174978:exon18:c.1570C>T:p.L524F	47.54	NM_174978
C14orf39	P1	14	60932752	60932752	Missense_Mutation	SNP	G	G	A	C14orf39:NM_174978:exon11:c.917C>T:p.A306V	65.63	NM_174978
SNW1	P1	14	78189588	78189588	Missense_Mutation	SNP	G	G	A	SNW1:NM_001318844:exon11:c.1066C>T:p.R356W	45	NM_001318844
BEND5	P1	1	49224895	49224895	Missense_Mutation	SNP	C	C	T	BEND5:NM_024603:exon3:c.422G>A:p.R141Q	28.18	NM_024603
ADAMTS7	P1	15	79058622	79058622	Missense_Mutation	SNP	G	G	A	ADAMTS7:NM_014272:exon19:c.3631C>T:p.R1211W	42.05	NM_014272
CACNA1H	P1	16	1259312	1259312	Missense_Mutation	SNP	G	G	A	CACNA1H:NM_021098:exon17:c.3644G>A:p.R1215H	47.78	NM_021098
PDILT	P1	16	20410533	20410533	Missense_Mutation	SNP	G	G	T	PDILT:NM_174924:exon2:c.90C>A:p.S30R	13.54	NM_174924
ARHGAP17	P1	16	24942383	24942383	Missense_Mutation	SNP	C	C	A	ARHGAP17:NM_001006634:exon19:c.2237G>T:p.G746V	22.93	NM_001006634
COG8	P1	16	69370591	69370591	Missense_Mutation	SNP	C	C	A	COG8:NM_032382:exon2:c.402G>T:p.E134D	56.45	NM_032382
GPR157	P1	1	9164626	9164626	Missense_Mutation	SNP	C	C	T	GPR157:NM_024980:exon4:c.860G>A:p.R287Q	17.07	NM_024980
IL12RB1	P1	19	18188352	18188352	Missense_Mutation	SNP	G	G	A	IL12RB1:NM_005535:exon5:c.523C>T:p.R175W	35.48	NM_005535
CSNK1G2	P1	19	1978973	1978973	Missense_Mutation	SNP	A	A	T	CSNK1G2:NM_001319:exon6:c.563A>T:p.D188V	16.57	NM_001319
ZNF682	P1	19	20116980	20116980	Missense_Mutation	SNP	T	T	C	ZNF682:NM_033196:exon4:c.1331A>G:p.K444R	28	NM_033196
ZNF235	P1	19	44792897	44792897	Missense_Mutation	SNP	T	T	C	ZNF235:NM_004234:exon5:c.691A>G:p.K231E	37.07	NM_004234
APOC2	P1	19	45452463	45452470	Frame_Shift_Ins	DEL	TTTTTACT	TTTTTACT	-	APOC2:NM_000483:exon4:c.263_270delTTTTTACT:p.I88fs	22.22	NM_000483
SHANK1	P1	19	51219930	51219930	Missense_Mutation	SNP	G	G	A	SHANK1:NM_016148:exon1:c.247C>T:p.H83Y	54.55	NM_016148

DENND1C	P1	19	6477251	6477251	Frame_Shift_Ins	DEL	G	G	-	DENND1C:NM_024898:exon8:c.491delC:p.P164fs	11.67	NM_024898
RALGAPA2	P1	20	20571844	20571844	Missense_Mutation	SNP	G	G	A	RALGAPA2:NM_020343:exon17:c.2318C>T:p.P773L	25.88	NM_020343
MCM3AP	P1	21	47695153	47695153	Missense_Mutation	SNP	G	G	A	MCM3AP:NM_003906:exon6:c.1945C>T:p.R649W	42.05	NM_003906
DNAH7	P1	2	196851900	196851900	Missense_Mutation	SNP	C	C	T	DNAH7:NM_018897:exon14:c.1644G>A:p.M548I	40.3	NM_018897
DNAH7	P1	2	196851911	196851911	Missense_Mutation	SNP	G	G	A	DNAH7:NM_018897:exon14:c.1633C>T:p.R545C	41.94	NM_018897
SPAG16	P1	2	214149289	214149289	Missense_Mutation	SNP	G	G	T	SPAG16:NM_024532:exon1:c.82G>T:p.D28Y	45.98	NM_024532
SRRD	P1	22	26879942	26879962	In_Frame_Del	DEL	GGAGGGAGGCGGCGCCCCGGG	GGAGGGAGGCGGCGCCCCGGG	-	SRRD:NM_001013694:exon1:c.90_110delGGAGGCGGCGCCCCGGGGGAG:p.E31_R37del	31.71	NM_001013694
VPS54	P1	2	64199317	64199317	Missense_Mutation	SNP	G	G	A	VPS54:NM_016516:exon4:c.440C>T:p.T147I	33.33	NM_016516
GCFC2	P1	2	75923413	75923413	Missense_Mutation	SNP	T	T	C	GCFC2:NM_003203:exon5:c.746A>G:p.N249S	51.61	NM_003203
GUCA1C	P1	3	108635036	108635036	Missense_Mutation	SNP	T	T	C	GUCA1C:NM_005459:exon3:c.380A>G:p.Q127R	19.54	NM_005459
PLA1A	P1	3	119338415	119338415	Missense_Mutation	SNP	G	G	T	PLA1A:NM_015900:exon8:c.928G>T:p.V310L	16.44	NM_015900
NPHP3	P1	3	132402305	132402305	Missense_Mutation	SNP	C	C	G	NPHP3:NM_153240:exon25:c.3634G>C:p.G1212R	30.77	NM_153240
C3orf33	P1	3	155520356	155520356	Missense_Mutation	SNP	C	C	T	C3orf33:NM_001308229:exon2:c.139G>A:p.A47T	44.74	NM_001308229
SKIL	P1	3	170110135	170110135	Missense_Mutation	SNP	A	A	G	SKIL:NM_005414:exon7:c.1985A>G:p.Q662R	30.77	NM_005414
ZNF385D	P1	3	21465505	21465505	Missense_Mutation	SNP	G	G	T	ZNF385D:NM_024697:exon7:c.904C>A:p.P302T	34.26	NM_024697
DOCK3	P1	3	51411972	51411972	Missense_Mutation	SNP	G	G	A	DOCK3:NM_004947:exon50:c.5366G>A:p.R1789H	28.13	NM_004947
CSN1S1	P1	4	70807771	70807771	Missense_Mutation	SNP	C	C	T	CSN1S1:NM_001890:exon13:c.350C>T:p.A117V	39.74	NM_001890
APC	P1	5	112174885	112174886	Frame_Shift_Ins	DEL	AA	AA	-	APC:NM_000038:exon16:c.3595_3596delAA:p.K1199fs	25.52	NM_000038
SYNE1	P1	6	152644715	152644715	Missense_Mutation	SNP	C	C	T	SYNE1:NM_182961:exon82:c.15815G>A:p.R5272Q	33.33	NM_182961
HSPA1L	P1	6	31778156	31778156	Missense_Mutation	SNP	C	C	T	HSPA1L:NM_005527:exon2:c.1594G>A:p.E532K	11.56	NM_005527
KLF14	P1	7	130418281	130418281	Missense_Mutation	SNP	G	G	A	KLF14:NM_138693:exon1:c.580C>T:p.R194C	20.19	NM_138693
BAG4	P1	8	38066607	38066607	Missense_Mutation	SNP	G	G	A	BAG4:NM_004874:exon4:c.710G>A:p.R237Q	33.33	NM_004874
VCPIP1	P1	8	67547410	67547410	Missense_Mutation	SNP	G	G	C	VCPIP1:NM_025054:exon3:c.2995C>G:p.P999A	25.85	NM_025054
OTUD6B	P1	8	92097062	92097062	Missense_Mutation	SNP	G	G	A	OTUD6B:NM_016023:exon7:c.938G>A:p.R313Q	47.87	NM_016023
IFNA6	P1	9	21350544	21350544	Missense_Mutation	SNP	G	G	T	IFNA6:NM_021002:exon1:c.343C>A:p.Q115K	30.77	NM_021002
C9orf64	P1	9	86559744	86559744	Missense_Mutation	SNP	T	T	C	C9orf64:NM_032307:exon3:c.758A>G:p.K253R	31.46	NM_032307
KRAS	P2	12	25398284	25398284	Missense_Mutation	SNP	C	C	G	KRAS:NM_033360:exon2:c.35G>C:p.G12A	8.82	NM_033360
SLC2A14	P2	12	7982501	7982501	Missense_Mutation	SNP	C	C	T	SLC2A14:NM_001286237:exon4:c.488G>A:p.R163H	16.07	NM_001286237
CLSPN	P2	1	36217056	36217056	Missense_Mutation	SNP	C	C	T	CLSPN:NM_022111:exon10:c.1823G>A:p.R608Q	19.54	NM_022111
SCEL	P2	13	78133971	78133971	Missense_Mutation	SNP	G	G	A	SCEL:NM_144777:exon4:c.194G>A:p.R65Q	16.67	NM_144777
CHGB	P2	20	5903841	5903841	Missense_Mutation	SNP	G	G	A	CHGB:NM_001819:exon4:c.1051G>A:p.V351I	15.79	NM_001819
KLHL6	P2	3	183226065	183226065	Missense_Mutation	SNP	C	C	T	KLHL6:NM_130446:exon3:c.691G>A:p.E231K	13.82	NM_130446
APC	P2	5	112175216	112175216	Nonsense_Mutation	SNP	G	G	T	APC:NM_000038:exon16:c.3925G>T:p.E1309*	74.81	NM_000038
ADAMTS16	P2	5	5262847	5262847	Nonsense_Mutation	SNP	C	C	T	ADAMTS16:NM_139056:exon18:c.2740C>T:p.R914*	31.17	NM_139056
ABCA13	P2	7	48314760	48314760	Missense_Mutation	SNP	C	C	T	ABCA13:NM_152701:exon17:c.5497C>T:p.R1833W	32.8	NM_152701
COL15A1	P2	9	101802807	101802807	Missense_Mutation	SNP	C	C	T	COL15A1:NM_001855:exon23:c.2480C>T:p.P827L	9.59	NM_001855
PAPPA	P2	9	118950362	118950362	Missense_Mutation	SNP	G	G	A	PAPPA:NM_002581:exon2:c.1345G>A:p.V449M	12.9	NM_002581
GPR50	P2	X	150349071	150349071	Missense_Mutation	SNP	G	G	A	GPR50:NM_004224:exon2:c.1016G>A:p.R339H	10.69	NM_004224
FOXJ2	P2	12	8200558	8200560	In_Frame_Del	DEL	CAG	CAG	-	FOXJ2:NM_018416:exon7:c.917_919delAGC:p.Q306del	16.07	NM_018416
CHAT	P2	10	50863221	50863221	Missense_Mutation	SNP	C	C	T	CHAT:NM_020549:exon12:c.1715C>T:p.S572L	22.22	NM_020549
PLCE1	P2	10	95995696	95995696	Missense_Mutation	SNP	G	G	T	PLCE1:NM_016341:exon7:c.2239G>T:p.D747Y	11.49	NM_016341
ANO5	P2	11	22239826	22239826	Missense_Mutation	SNP	G	G	A	ANO5:NM_213599:exon4:c.173G>A:p.R58Q	35.42	NM_213599
PSMD13	P2	11	251559	251559	Missense_Mutation	SNP	G	G	C	PSMD13:NM_175932:exon9:c.857G>C:p.R286P	10.47	NM_175932
OR4A16	P2	11	55111584	55111584	Missense_Mutation	SNP	A	A	T	OR4A16:NM_001005274:exon1:c.908A>T:p.K303M	55.88	NM_001005274
UBQLN3	P2	11	5529465	5529465	Missense_Mutation	SNP	G	G	T	UBQLN3:NM_017481:exon2:c.1324C>A:p.P442T	15.25	NM_017481
SPTA1	P2	1	158584091	158584091	Missense_Mutation	SNP	A	A	G	SPTA1:NM_003126:exon49:c.6794T>C:p.I2265T	36.84	NM_003126
PRG4	P2	1	186273994	186273994	Missense_Mutation	SNP	C	C	T	PRG4:NM_005807:exon6:c.538C>T:p.R180W	57.14	NM_005807
TMCC2	P2	1	205241235	205241235	Missense_Mutation	SNP	G	G	A	TMCC2:NM_014858:exon5:c.2113G>A:p.V705M	12.61	NM_014858
PIK3C2G	P2	12	18435399	18435401	In_Frame_Del	DEL	CCC	CCC	-	PIK3C2G:NM_001288772:exon2:c.385_387delCCC:p.P129del	35.94	NM_001288772
PIK3C2G	P2	12	18435452	18435452	Missense_Mutation	SNP	C	C	T	PIK3C2G:NM_001288772:exon2:c.437C>T:p.P146L	38.6	NM_001288772
MRPS35	P2	12	27867727	27867727	Missense_Mutation	SNP	G	G	A	MRPS35:NM_021821:exon2:c.127G>A:p.G43R	36.07	NM_021821
MBD6	P2	12	57918525	57918525	Missense_Mutation	SNP	T	T	C	MBD6:NM_052897:exon4:c.136T>C:p.S46P	29.31	NM_052897
PTPN6	P2	12	7064899	7064899	Missense_Mutation	SNP	G	G	T	PTPN6:NM_080549:exon7:c.824G>T:p.R275L	22.92	NM_080549
WNK1	P2	12	970483	970483	Missense_Mutation	SNP	A	A	G	WNK1:NM_213655:exon7:c.1925A>G:p.Y642C	12.07	NM_213655
CCDC168	P2	13	103397057	103397057	Nonsense_Mutation	SNP	G	G	T	CCDC168:NM_001146197:exon4:c.5990C>A:p.S1997*	12.23	NM_001146197
SETDB2	P2	13	50057097	50057097	Missense_Mutation	SNP	G	G	A	SETDB2:NM_031915:exon10:c.1417G>A:p.V473M	56.82	NM_031915
TP73	P2	1	3647535	3647535	Missense_Mutation	SNP	G	G	A	TP73:NM_005427:exon12:c.1390G>A:p.G464S	16.79	NM_005427
OXGR1	P2	13	97639622	97639622	Missense_Mutation	SNP	C	C	T	OXGR1:NM_080818:exon4:c.392G>A:p.R131H	29.55	NM_080818
PLD4	P2	14	105398401	105398401	Missense_Mutation	SNP	G	G	A	PLD4:NM_001308174:exon9:c.1132G>A:p.V378M	15.79	NM_001308174
CHGA	P2	14	93399045	93399045	Missense_Mutation	SNP	C	C	G	CHGA:NM_001275:exon7:c.1139C>G:p.A380G	23.87	NM_001275
SCNN1B	P2	16	23391982	23391982	Missense_Mutation	SNP	G	G	C	SCNN1B:NM_000336:exon13:c.1783G>C:p.A595P	10.13	NM_000336
MYO15A	P2	17	18024806	18024806	Missense_Mutation	SNP	G	G	A	MYO15A:NM_016239:exon2:c.2692G>A:p.G898R	13.51	NM_016239
UTP6	P2	17	30222002	30222002	Missense_Mutation	SNP	T	T	C	UTP6:NM_018428:exon3:c.206A>G:p.Q69R	65	NM_018428
ARRB2	P2	17	4619845	4619845	Missense_Mutation	SNP	G	G	A	ARRB2:NM_001257328:exon5:c.299G>A:p.R100H	12.23	NM_001257328
AXIN2	P2	17	63554666	63554666	Missense_Mutation	SNP	G	G	A	AXIN2:NM_004655:exon2:c.73C>T:p.P25S	28.43	NM_004655
ZNF521	P2	18	22806051	22806051	Missense_Mutation	SNP	C	C	A	ZNF521:NM_015461:exon4:c.1831G>T:p.A611S	20.31	NM_015461
CBLN2	P2	18	70209101	70209101	Missense_Mutation	SNP	G	G	A	CBLN2:NM_182511:exon3:c.295C>T:p.R99W	14.42	NM_182511
OR1I1	P2	19	15198488	15198488	Missense_Mutation	SNP	T	T	G	OR1I1:NM_001004713:exon1:c.612T>G:p.I204M	13.33	NM_001004713
SCAF1	P2	19	50155362	50155367	In_Frame_Del	DEL	CCGCTC	CCGCTC	-	SCAF1:NM_021228:exon7:c.1741_1746delCGCTCC:p.R581_S582del	41.18	NM_021228
NLRP2	P2	19	55494753	55494753	Missense_Mutation	SNP	G	G	A	NLRP2:NM_017852:exon6:c.1687G>A:p.E563K	12.5	NM_017852
ZSCAN5B	P2	19	56703269	56703269	Missense_Mutation	SNP	G	G	A	ZSCAN5B:NM_001080456:exon2:c.538C>T:p.R180C	25.51	NM_001080456
PEG3	P2	19	57326319	57326319	Missense_Mutation	SNP	T	T	C	PEG3:NM_006210:exon10:c.3491A>G:p.E1164G	16.67	NM_006210
ROCK2	P2	2	11359120	11359120	Missense_Mutation	SNP	G	G	T	ROCK2:NM_004850:exon10:c.1292C>A:p.T431N	50	NM_004850
RBM11	P2	21	15596772	15596772	Missense_Mutation	SNP	T	T	G	RBM11:NM_001320602:exon4:c.346T>G:p.L116V	47.37	NM_001320602
NMI	P2	2	152139416	152139416	Missense_Mutation	SNP	G	G	A	NMI:NM_004688:exon2:c.47C>T:p.S16L	33.33	NM_004688
GRB14	P2	2	165365287	165365288	Frame_Shift_Ins	INS	-	-	T	GRB14:NM_004490:exon7:c.891dupA:p.H298fs	11.94	NM_004490
TIMP4	P2	3	12198364	12198364	Missense_Mutation	SNP	C	C	T	TIMP4:NM_003256:exon3:c.308G>A:p.G103D	25.93	NM_003256
ATR	P2	3	142274739	142274740	Frame_Shift_Ins	INS	-	-	T	ATR:NM_001184:exon10:c.2320dupA:p.I774fs	18.42	NM_001184
ZBBX	P2	3	167051769	167051769	Missense_Mutation	SNP	T	T	C	ZBBX:NM_001199201:exon10:c.533A>G:p.K178R	41.94	NM_001199201
TNK2	P2	3	195609039	195609039	Missense_Mutation	SNP	T	T	C	TNK2:NM_001010938:exon6:c.959A>G:p.N320S	18.42	NM_001010938
OR5H6	P2	3	97983711	97983711	Missense_Mutation	SNP	T	T	C	OR5H6:NM_001005479:exon1:c.583T>C:p.C195R	69.23	NM_001005479
ENPEP	P2	4	111397731	111397731	Missense_Mutation	SNP	C	C	T	ENPEP:NM_001977:exon1:c.161C>T:p.A54V	10.34	NM_001977

CCDC110	P2	4	186380243	186380243	Missense_Mutation	SNP	A	A	C	CCDC110:NM_152775:exon6:c.1498T>G:p.Y500D	63.33	NM_152775
KLKB1	P2	4	187178473	187178473	Missense_Mutation	SNP	G	G	A	KLKB1:NM_000892:exon14:c.1679G>A:p.R560Q	37.93	NM_000892
SORCS2	P2	4	7728561	7728561	Missense_Mutation	SNP	G	G	A	SORCS2:NM_020777:exon21:c.2800G>A:p.V934M	15.15	NM_020777
FRAS1	P2	4	79367890	79367890	Missense_Mutation	SNP	G	G	A	FRAS1:NM_025074:exon43:c.5866G>A:p.D1956N	19.74	NM_025074
SPINK5	P2	5	147499645	147499645	Missense_Mutation	SNP	G	G	A	SPINK5:NM_006846:exon25:c.2387G>A:p.R796Q	32.47	NM_006846
ADAMTS6	P2	5	64756126	64756126	Missense_Mutation	SNP	C	C	T	ADAMTS6:NM_197941:exon4:c.502G>A:p.E168K	15.87	NM_197941
POC5	P2	5	75001582	75001582	Missense_Mutation	SNP	A	A	G	POC5:NM_001099271:exon4:c.254T>C:p.I85T	60.87	NM_001099271
IQGAP2	P2	5	75927738	75927738	Missense_Mutation	SNP	A	A	T	IQGAP2:NM_006633:exon15:c.1667A>T:p.D556V	16.52	NM_006633
ZBTB2	P2	6	151687636	151687636	Missense_Mutation	SNP	G	G	A	ZBTB2:NM_020861:exon3:c.565C>T:p.R189W	19.63	NM_020861
RSPH3	P2	6	159399348	159399348	Missense_Mutation	SNP	A	A	G	RSPH3:NM_031924:exon7:c.1316T>C:p.M439T	36.84	NM_031924
IMPG1	P2	6	76631823	76631823	Missense_Mutation	SNP	C	C	T	IMPG1:NM_001563:exon17:c.2377G>A:p.D793N	41.67	NM_001563
CTTNBP2	P2	7	117450845	117450845	Missense_Mutation	SNP	C	C	T	CTTNBP2:NM_033427:exon3:c.388G>A:p.A130T	43.59	NM_033427
WEE2	P2	7	141429437	141429437	Missense_Mutation	SNP	G	G	C	WEE2:NM_001105558:exon11:c.1642G>C:p.V548L	10.26	NM_001105558
PKD1L1	P2	7	47917119	47917119	Missense_Mutation	SNP	C	C	T	PKD1L1:NM_138295:exon22:c.3631G>A:p.V1211I	13.1	NM_138295
GUSB	P2	7	65441045	65441045	Missense_Mutation	SNP	G	G	A	GUSB:NM_000181:exon5:c.869C>T:p.P290L	10.34	NM_000181
PUF60	P2	8	144899294	144899294	Missense_Mutation	SNP	G	G	C	PUF60:NM_078480:exon11:c.1166C>G:p.P389R	27.84	NM_078480
FOCAD	P2	9	20819889	20819889	Missense_Mutation	SNP	G	G	T	FOCAD:NM_017794:exon14:c.1550G>T:p.G517V	42.86	NM_017794
RORB	P2	9	77257699	77257699	Missense_Mutation	SNP	A	A	G	RORB:NM_006914:exon4:c.605A>G:p.Q202R	30.34	NM_006914
FOXR2	P2	X	55650629	55650629	Missense_Mutation	SNP	C	C	A	FOXR2:NM_198451:exon1:c.485C>A:p.P162Q	18.87	NM_198451
ELMOD1	P3	11	107502336	107502336	Missense_Mutation	SNP	G	G	A	ELMOD1:NM_018712:exon5:c.223G>A:p.D75N	10.67	NM_018712
WSCD2	P3	12	108603974	108603974	Missense_Mutation	SNP	G	G	A	WSCD2:NM_014653:exon4:c.574G>A:p.E192K	5.45	NM_014653
OBSCN	P3	1	228444454	228444454	Missense_Mutation	SNP	C	C	T	OBSCN:NM_001271223:exon16:c.4688C>T:p.T1563M	32.56	NM_001271223
SV2B	P3	15	91769716	91769716	Missense_Mutation	SNP	C	C	T	SV2B:NM_014848:exon3:c.223C>T:p.R75W	5.69	NM_014848
C16orf78	P3	16	49430376	49430376	Missense_Mutation	SNP	G	G	A	C16orf78:NM_144602:exon4:c.437G>A:p.R146Q	14	NM_144602
EDC4	P3	16	67912294	67912294	Missense_Mutation	SNP	C	C	T	EDC4:NM_014329:exon9:c.1036C>T:p.R346W	15.12	NM_014329
PSG9	P3	19	43762546	43762546	Missense_Mutation	SNP	C	C	T	PSG9:NM_002784:exon5:c.1051G>A:p.D351N	13.69	NM_002784
NR1H2	P3	19	50882412	50882412	Missense_Mutation	SNP	G	G	A	NR1H2:NM_007121:exon7:c.901G>A:p.A301T	12.9	NM_007121
SMARCB1	P3	22	24133967	24133967	Nonsense_Mutation	SNP	C	C	T	SMARCB1:NM_003073:exon2:c.118C>T:p.R40*	13.33	NM_003073
SULT6B1	P3	2	37406711	37406711	Missense_Mutation	SNP	C	C	T	SULT6B1:NM_001032377:exon4:c.305G>A:p.R102Q	12.24	NM_001032377
APC	P3	5	112173917	112173917	Nonsense_Mutation	SNP	C	C	T	APC:NM_000038:exon16:c.2626C>T:p.R876*	26.52	NM_000038
APC	P3	5	112175618	112175618	Frame_Shift_Ins	DEL	C	C	-	APC:NM_000038:exon16:c.4328delC:p.P1443fs	39.16	NM_000038
PITRM1	P3	10	3208567	3208568	Frame_Shift_Ins	INS	-	-	GCACGCTAGGGAAGAGAGAGGAATG	PITRM1:NM_001242307:exon4:c.271_272insCATTCCTCTCTCTTCCCTAGCGTGC:p.Q91fs	41.05	NM_001242307
LDB3	P3	10	88451678	88451678	Missense_Mutation	SNP	G	G	A	LDB3:NM_007078:exon5:c.715G>A:p.V239I	13.98	NM_007078
PKLR	P3	1	155269931	155269931	Missense_Mutation	SNP	C	C	T	PKLR:NM_000298:exon2:c.241G>A:p.E81K	11.11	NM_000298
TSKU	P3	11	76507688	76507688	Missense_Mutation	SNP	G	G	A	TSKU:NM_001318477:exon2:c.1070G>A:p.R357Q	18.46	NM_001318477
TENM4	P3	11	78498012	78498012	Missense_Mutation	SNP	C	C	T	TENM4:NM_001098816:exon16:c.2296G>A:p.E766K	25.53	NM_001098816
TENM4	P3	11	78565286	78565286	Missense_Mutation	SNP	C	C	T	TENM4:NM_001098816:exon12:c.1544G>A:p.R515H	15.38	NM_001098816
LAMC1	P3	1	183105592	183105592	Nonsense_Mutation	SNP	C	C	T	LAMC1:NM_002293:exon25:c.4186C>T:p.Q1396*	19.35	NM_002293
IKBKE	P3	1	206669465	206669465	Missense_Mutation	SNP	C	C	T	IKBKE:NM_014002:exon22:c.2138C>T:p.P713L	45.83	NM_014002
KNTC1	P3	12	123057743	123057743	Missense_Mutation	SNP	C	C	T	KNTC1:NM_014708:exon26:c.2194C>T:p.L732F	21.17	NM_014708
SOX1	P3	13	112723111	112723112	In_Frame_Ins	INS	-	-	GGGCGT	SOX1:NM_005986:exon1:c.1141_1146dupGGCGTG:p.G381_V382dup	42.42	NM_005986
LRP8	P3	1	53746310	53746310	Missense_Mutation	SNP	C	C	T	LRP8:NM_004631:exon4:c.445G>A:p.D149N	13.91	NM_004631
C15orf39	P3	15	75499490	75499490	Missense_Mutation	SNP	C	C	G	C15orf39:NM_015492:exon2:c.1101C>G:p.C367W	28.57	NM_015492
ISL2	P3	15	76634135	76634135	Missense_Mutation	SNP	G	G	T	ISL2:NM_145805:exon6:c.1039G>T:p.D347Y	13.64	NM_145805
TACSTD2	P3	1	59042494	59042494	Missense_Mutation	SNP	C	C	T	TACSTD2:NM_002353:exon1:c.335G>A:p.G112D	13.25	NM_002353
SSTR5	P3	16	1129733	1129733	Missense_Mutation	SNP	G	G	A	SSTR5:NM_001053:exon1:c.865G>A:p.V289M	11.28	NM_001053
TXNDC11	P3	16	11836508	11836508	Missense_Mutation	SNP	G	G	A	TXNDC11:NM_001303447:exon1:c.79C>T:p.P27S	46.67	NM_001303447
DNAH3	P3	16	20990740	20990740	Missense_Mutation	SNP	G	G	A	DNAH3:NM_017539:exon50:c.7988C>T:p.T2663M	38.24	NM_017539
BICDL2	P3	16	3079685	3079685	Missense_Mutation	SNP	T	T	C	BICDL2:NM_001103175:exon5:c.818A>G:p.Q273R	60	NM_001103175
RBFOX1	P3	16	7703915	7703915	Missense_Mutation	SNP	G	G	A	RBFOX1:NM_001308117:exon12:c.985G>A:p.A329T	22.22	NM_001308117
HASPIN	P3	17	3628656	3628656	Missense_Mutation	SNP	G	G	A	HASPIN:NM_031965:exon1:c.1427G>A:p.S476N	13.74	NM_031965
TBX21	P3	17	45822289	45822289	Missense_Mutation	SNP	G	G	C	TBX21:NM_013351:exon6:c.1165G>C:p.A389P	11.76	NM_013351
UTP18	P3	17	49357831	49357831	Missense_Mutation	SNP	G	G	A	UTP18:NM_016001:exon9:c.1195G>A:p.A399T	17.54	NM_016001
COG1	P3	17	71197890	71197890	Missense_Mutation	SNP	C	C	A	COG1:NM_018714:exon7:c.1924C>A:p.P642T	41.35	NM_018714
KCNG2	P3	18	77623854	77623854	Missense_Mutation	SNP	G	G	A	KCNG2:NM_012283:exon1:c.187G>A:p.D63N	14.85	NM_012283
PAK4	P3	19	39663643	39663643	Missense_Mutation	SNP	C	C	A	PAK4:NM_005884:exon4:c.290C>A:p.S97Y	23.88	NM_005884
LRFN1	P3	19	39805333	39805333	Missense_Mutation	SNP	A	A	T	LRFN1:NM_020862:exon1:c.644T>A:p.L215Q	21.43	NM_020862
SPTBN4	P3	19	41018705	41018705	Missense_Mutation	SNP	C	C	T	SPTBN4:NM_020971:exon14:c.2009C>T:p.A670V	42.22	NM_020971
MADCAM1	P3	19	501701	501701	Missense_Mutation	SNP	G	G	A	MADCAM1:NM_130760:exon4:c.700G>A:p.D234N	48.65	NM_130760
MADCAM1	P3	19	501719	501719	Missense_Mutation	SNP	T	T	C	MADCAM1:NM_130760:exon4:c.718T>C:p.S240P	67.5	NM_130760
R3HDM4	P3	19	899617	899617	Missense_Mutation	SNP	C	C	T	R3HDM4:NM_138774:exon6:c.631G>A:p.A211T	12.68	NM_138774
SAP130	P3	2	128773988	128773988	Missense_Mutation	SNP	G	G	A	SAP130:NM_001145928:exon4:c.560C>T:p.P187L	31.09	NM_001145928
URB1	P3	21	33694176	33694176	Missense_Mutation	SNP	G	G	A	URB1:NM_014825:exon34:c.5419C>T:p.R1807W	30.85	NM_014825
NEB	P3	2	152420351	152420351	Missense_Mutation	SNP	G	G	T	NEB:NM_001271208:exon118:c.18565C>A:p.L6189M	18.75	NM_001271208
SCARF2	P3	22	20780026	20780026	Missense_Mutation	COMPLEX	GCGG	GCGG	CGCGGC	SCARF2:NM_153334:exon12:c.2247_2250delCCGCinsGCCGCG:p.R750delinsP?	11.54	NM_153334
P2RX6	P3	22	21377288	21377288	Nonsense_Mutation	SNP	G	G	A	P2RX6:NM_005446:exon5:c.521G>A:p.W174*	32.61	NM_005446
ZFP36L2	P3	2	43451955	43451955	Frame_Shift_Ins	COMPLEX	CAGCC	CAGCC	GCT	ZFP36L2:NM_006887:exon2:c.984_988delGGCTGinsAGC:p.A330fs	26.32	NM_006887
TMEM247	P3	2	46707851	46707851	Missense_Mutation	SNP	G	G	A	TMEM247:NM_001145051:exon2:c.425G>A:p.R142Q	59.84	NM_001145051
CCDC80	P3	3	112358175	112358175	Missense_Mutation	SNP	G	G	A	CCDC80:NM_199511:exon2:c.578C>T:p.A193V	39.55	NM_199511
WNT5A	P3	3	55504410	55504410	Missense_Mutation	SNP	C	C	T	WNT5A:NM_003392:exon5:c.853G>A:p.V285I	37.97	NM_003392
BHMT2	P3	5	78384318	78384318	Missense_Mutation	SNP	C	C	A	BHMT2:NM_017614:exon8:c.1013C>A:p.A338D	13.33	NM_017614
IP6K3	P3	6	33695880	33695880	Missense_Mutation	SNP	G	G	A	IP6K3:NM_054111:exon3:c.397C>T:p.R133C	15.56	NM_054111
UHRF1BP1	P3	6	34826040	34826040	Missense_Mutation	SNP	G	G	A	UHRF1BP1:NM_017754:exon14:c.1907G>A:p.R636Q	14.29	NM_017754
RCAN2	P3	6	46190991	46190991	Missense_Mutation	SNP	C	C	T	RCAN2:NM_001251973:exon5:c.619G>A:p.V207I	22.79	NM_001251973
WDR86	P3	7	151082198	151082198	Missense_Mutation	SNP	C	C	T	WDR86:NM_001284260:exon4:c.902G>A:p.R301H	32.39	NM_001284260
ABCA13	P3	7	48273718	48273718	Missense_Mutation	SNP	G	G	T	ABCA13:NM_152701:exon8:c.867G>T:p.Q289H	12.5	NM_152701
GSDMD	P3	8	144641557	144641557	Missense_Mutation	SNP	C	C	G	GSDMD:NM_024736:exon2:c.52C>G:p.H18D	13.79	NM_024736
SCRIB	P3	8	144887461	144887461	Missense_Mutation	SNP	C	C	T	SCRIB:NM_182706:exon19:c.2491G>A:p.E831K	11.46	NM_182706
CSMD1	P3	8	2857534	2857534	Missense_Mutation	SNP	C	C	T	CSMD1:NM_033225:exon53:c.8149G>A:p.V2717M	15.85	NM_033225
STAB2	P4	12	104105290	104105290	Missense_Mutation	SNP	G	G	A	STAB2:NM_017564:exon40:c.4330G>A:p.A1444T	35.09	NM_017564
GJA4	P4	1	35260514	35260514	Missense_Mutation	SNP	C	C	T	GJA4:NM_002060:exon2:c.700C>T:p.R234C	17.95	NM_002060

SLC6A11	P4	3	10885924	10885924	Missense_Mutation	SNP	G	G	A	SLC6A11:NM_014229:exon5:c.649G>A:p.G217R	22.33	NM_014229
FBXW7	P4	4	153245447	153245447	Missense_Mutation	SNP	A	A	G	FBXW7:NM_033632:exon11:c.1744T>C:p.S582P	6	NM_033632
APC	P4	5	112173917	112173917	Nonsense_Mutation	SNP	C	C	T	APC:NM_000038:exon16:c.2626C>T:p.R876*	29.85	NM_000038
APC	P4	5	112175639	112175639	Nonsense_Mutation	SNP	C	C	T	APC:NM_000038:exon16:c.4348C>T:p.R1450*	29.41	NM_000038
CRTAC1	P4	10	99696056	99696056	Missense_Mutation	SNP	C	C	T	CRTAC1:NM_018058:exon3:c.292G>A:p.D98N	17.02	NM_018058
CNTN5	P4	11	100061886	100061886	Missense_Mutation	SNP	C	C	T	CNTN5:NM_014361:exon14:c.1609C>T:p.R537W	30.09	NM_014361
KDELC2	P4	11	108350085	108350085	Missense_Mutation	SNP	A	A	C	KDELC2:NM_153705:exon6:c.1236T>G:p.H412Q	19.3	NM_153705
ARHGEF12	P4	11	120351125	120351125	Missense_Mutation	SNP	A	A	G	ARHGEF12:NM_015313:exon38:c.4223A>G:p.N1408S	40.71	NM_015313
IVL	P4	1	152883680	152883680	Missense_Mutation	SNP	A	A	T	IVL:NM_005547:exon2:c.1407A>T:p.Q469H	33.59	NM_005547
OR51B2	P4	11	5345158	5345158	Missense_Mutation	SNP	T	T	C	OR51B2:NM_033180:exon1:c.370A>G:p.I124V	32.24	NM_033180
NES	P4	1	156642746	156642748	In_Frame_Del	DEL	GAG	GAG	-	NES:NM_006617:exon4:c.1232_1234delCTC:p.P411del	29.21	NM_006617
NME7	P4	1	169293692	169293692	Missense_Mutation	SNP	T	T	C	NME7:NM_013330:exon2:c.50A>G:p.N17S	29.46	NM_013330
PITPNM2	P4	12	123485694	123485694	Missense_Mutation	SNP	C	C	G	PITPNM2:NM_020845:exon8:c.1060G>C:p.D354H	27.94	NM_020845
SMYD2	P4	1	214504326	214504326	Missense_Mutation	SNP	G	G	A	SMYD2:NM_020197:exon9:c.850G>A:p.D284N	35	NM_020197
CAPZA3	P4	12	18891215	18891215	Missense_Mutation	SNP	G	G	A	CAPZA3:NM_033328:exon1:c.13G>A:p.V5M	39.08	NM_033328
CACNA2D4	P4	12	1993428	1993428	Nonsense_Mutation	SNP	C	C	T	CACNA2D4:NM_172364:exon12:c.1332G>A:p.W444*	32.94	NM_172364
HLX	P4	1	221053600	221053600	Missense_Mutation	SNP	A	A	C	HLX:NM_021958:exon1:c.401A>C:p.Q134P	28.04	NM_021958
EPB42	P4	15	43512998	43512998	Missense_Mutation	SNP	C	C	T	EPB42:NM_000119:exon1:c.26G>A:p.R9H	35.85	NM_000119
CLUH	P4	17	2595897	2595897	Missense_Mutation	SNP	C	C	T	CLUH:NM_015229:exon21:c.3289G>A:p.G1097R	34.04	NM_015229
SARM1	P4	17	26699196	26699196	Frame_Shift_Ins	COMPLEX	CGGGCCCGCGA	CGGGCCCGCGA	GCGGGCCCCGAG	SARM1:NM_015077:exon2:c.143_150delCGGGCCCGCGAinsGCGGGCCCCGAG:p.P48fs	15.38	NM_015077
TMEM99	P4	17	38991247	38991247	Missense_Mutation	SNP	A	A	C	TMEM99:NM_145274:exon3:c.479A>C:p.H160P	26.25	NM_145274
DNAH17	P4	17	76451776	76451776	Missense_Mutation	SNP	G	G	A	DNAH17:NM_173628:exon63:c.10120C>T:p.R3374W	24.42	NM_173628
ZNF878	P4	19	12154799	12154799	Missense_Mutation	SNP	G	G	C	ZNF878:NM_001080404:exon4:c.1417C>G:p.H473D	45.45	NM_001080404
FCHO1	P4	19	17875219	17875219	Missense_Mutation	SNP	C	C	T	FCHO1:NM_001161357:exon6:c.155C>T:p.A52V	26.44	NM_001161357
GMIP	P4	19	19747825	19747825	Missense_Mutation	SNP	T	T	C	GMIP:NM_016573:exon12:c.1138A>G:p.I380V	45.28	NM_016573
TJP3	P4	19	3746010	3746010	Missense_Mutation	SNP	G	G	T	TJP3:NM_001267561:exon16:c.1968G>T:p.E656D	28.57	NM_001267561
ZNF446	P4	19	58991828	58991828	Missense_Mutation	SNP	C	C	T	ZNF446:NM_017908:exon7:c.1088C>T:p.P363L	41.98	NM_017908
TGM6	P4	20	2384368	2384368	Missense_Mutation	SNP	G	G	A	TGM6:NM_198994:exon9:c.1235G>A:p.R412H	29.63	NM_198994
FAM83D	P4	20	37555158	37555158	Missense_Mutation	SNP	A	A	G	FAM83D:NM_030919:exon1:c.73A>G:p.T25A	32.5	NM_030919
RIPOR3	P4	20	49219025	49219025	Missense_Mutation	SNP	G	G	A	RIPOR3:NM_001290268:exon13:c.1243C>T:p.P415S	30.38	NM_001290268
FAM171B	P4	2	187559029	187559030	In_Frame_Ins	INS	-	-	CAG	FAM171B:NM_177454:exon1:c.147_149dupGCA:p.Q50dup	17.65	NM_177454
HIC2	P4	22	21800776	21800776	Missense_Mutation	SNP	G	G	A	HIC2:NM_015094:exon3:c.1592G>A:p.R531Q	24.81	NM_015094
GIGYF2	P4	2	233660881	233660881	Missense_Mutation	SNP	A	A	G	GIGYF2:NM_001103147:exon16:c.1652A>G:p.H551R	32.14	NM_001103147
DRC1	P4	2	26676222	26676222	Missense_Mutation	SNP	C	C	T	DRC1:NM_145038:exon14:c.1724C>T:p.A575V	37.5	NM_145038
SLC25A36	P4	3	140678306	140678306	Missense_Mutation	SNP	G	G	T	SLC25A36:NM_001104647:exon3:c.208G>T:p.V70L	33.33	NM_001104647
NLGN1	P4	3	173998554	173998554	Missense_Mutation	SNP	C	C	T	NLGN1:NM_014932:exon7:c.1933C>T:p.P645S	35.67	NM_014932
ARAP3	P4	5	141052442	141052442	Missense_Mutation	SNP	G	G	A	ARAP3:NM_022481:exon8:c.1144C>T:p.R382C	31.25	NM_022481
IRF5	P4	7	128587344	128587373	In_Frame_Del	DEL	GGCCGCCCACTCTGCAGCCGCCCACTCTGC	GGCCGCCCACTCTGCAGCCGCCCACTCTGC	-	IRF5:NM_001098629:exon6:c.549_578delCACTCTGCAGCCGCCCACTCTGCGGCCGCC:p.T184_P193d	28.95	NM_001098629
ETV1	P4	7	13940436	13940436	Missense_Mutation	SNP	T	T	C	ETV1:NM_004956:exon13:c.1139A>G:p.N380S	35.04	NM_004956
KEL	P4	7	142658528	142658528	Missense_Mutation	SNP	C	C	T	KEL:NM_000420:exon3:c.142G>A:p.V48M	25.58	NM_000420
PCLO	P4	7	82784832	82784832	Missense_Mutation	SNP	C	C	G	PCLO:NM_033026:exon2:c.1125G>C:p.Q375H	53.62	NM_033026
ODF1	P4	8	103573006	103573006	Missense_Mutation	SNP	G	G	A	ODF1:NM_024410:exon2:c.647G>A:p.S216N	52.42	NM_024410
C9orf84	P4	9	114500617	114500617	Missense_Mutation	SNP	A	A	G	C9orf84:NM_173521:exon10:c.1168T>C:p.S390P	40	NM_173521
GBA2	P4	9	35738334	35738346	Frame_Shift_Ins	DEL	CAGCCACAGCTGC	CAGCCACAGCTGC	-	GBA2:NM_020944:exon14:c.2080_2092delGCAGCTGTGGCTG:p.A69	19.53	NM_020944
IGSF1	P4	X	130410160	130410160	Missense_Mutation	SNP	C	C	A	IGSF1:NM_001170961:exon15:c.2686G>T:p.V896L	33.33	NM_001170961
AMER1	P4	X	63412647	63412648	Frame_Shift_Ins	INS	-	-	A	AMER1:NM_152424:exon2:c.519dupT:p.S174fs	65.15	NM_152424
